# 20-year follow-up study of Danish HHT patients—survival and causes of death

**DOI:** 10.1186/s13023-016-0533-9

**Published:** 2016-11-22

**Authors:** Anette Kjeldsen, Katrine Saldern Aagaard, Pernille Mathiesen Tørring, Sören Möller, Anders Green

**Affiliations:** 1 HHT-center OUH, Department of Otorhinolaryngology, Odense, Denmark; 2 Clinical Institute, University of Southern Denmark, Odense, Denmark; 3 Department of Clinical Genetics, Odense, Denmark; 4 OPEN, Odense Patient data Explorative Network, Odense University Hospital, Odense, Denmark; 5 Department of Clinical Research, University of Southern Denmark, Odense, Denmark

**Keywords:** Telangiectasia Haemorrhagic Hereditary, HHT, Survival, Cancer, Causes of death

## Abstract

**Background:**

Hereditary Haemorrhagic Telangiectasia (HHT) is a dominantly inheritable disorder, with a wide variety of clinical manifestations due to presence of multiple arteriovenous manifestations. The most common mutations are found in HHT1 (*ENG*) and HHT2 (*ACVRL1*) patients, causing alterations in the TGF-β pathway which is responsible for angiogenesis. Modulations of angiogenesis may influence cancer rates. The objective of the study was to evaluate 20-year survival according to HHT subtype, as well as to evaluate differences in causes of death comparing HHT patients and controls. We also wanted to investigate whether cancer morbidity among HHT patients differs from that among controls.

**Results:**

We included all HHT patients in the County of Fyn, Denmark, prevalent as of January 1st 1995 in total 73 HHT patients. In addition three age- and sex- matched controls per HHT patient were evaluated, in total 218 controls (one was lost due to registration failure). The controls were defined at start of follow-up in 1995. Information on lifestyle factors was not available. A total of 32 (44%) HHT patients and 97 (44%) controls passed away during follow-up. The survival curves were evenly distributed showing similar survival rates in the two groups. Cancer diagnoses had been registered in the follow-up period in 4 (5%) HHT patients and in 38 (17%) controls.

**Conclusion:**

The mortality was not increased among Danish HHT patients compared to controls. This study is based on a clinical unselected series of HHT patients with the whole spectrum of severity, independent of need for medical care. Our data also suggest that HHT patients to a lesser degree than the background population are affected by cancer.

**Electronic supplementary material:**

The online version of this article (doi:10.1186/s13023-016-0533-9) contains supplementary material, which is available to authorized users.

## Background

Hereditary Hemorrhagic Telangiectasia (HHT), also known as Osler-Weber-Rendu disease, is an autosomal dominant disorder characterized by presence of multiple arteriovenous malformations (AVMs) leading to wide variety of clinical manifestations [1, 2]. Case reports on fatal outcome in severe HHT cases have been reported [3, 4]. However, studies on survival in the overall HHT population have been sparse [5, 6]. Studies indicate that, at least in untreated HHT populations, an increased mortality seems to be present [7]. We have previously studied the mortality among HHT patients and controls in the county of Fyn [1, 8], and we identified a slightly increased mortality in a subgroup of patients. Studies of mortality are important to be able to advise patients on prognosis, and to gain information regarding the nature of the disease.

### Clinical manifestations of HHT

**Fig. 1:**
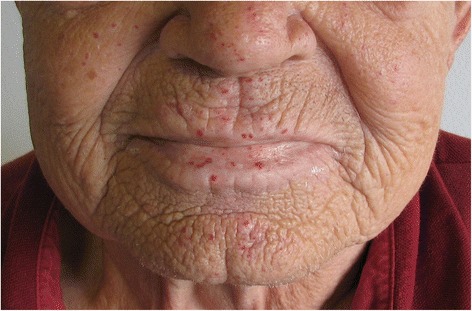
Typical telangiectatic lesion in an HHT patient

The most common clinical manifestation of HHT is spontaneous and recurrent epistaxis, usually with debut in childhood, ultimately affecting around 95% of all HHT patients. The epistaxis severity differs considerably between individual patients. With age HHT patients develop characteristic red spots (Fig. 1 (telangiectatic lesions)). Gastrointestinal AVM (GI-AVM) may cause gastrointestinal bleeding in 25% of HHT patients [2, 9] who often need blood transfusions. Pulmonary arteriovenous malformations (PAVMs) are present in around 12–50% depending on the genetic subtype [10–12]. PAVMs may cause serious neurological symptoms like cerebral abscess (CA) [13–15] or stroke, due to paradoxical embolism [10, 16]. Patients with PAVMs are therefore recommended treated with embolization, whenever possible [17]. Other neurological symptoms may be caused by cerebral arteriovenous malformations (CAVMs), which are present in at least 10% of patients [18]. Hepatic arteriovenous malformations (HAVMs) are quite common, but rarely symptomatic [2, 19]. Clinical reports show an apparent increased occurrence of bacterial infection [13–15], and an apparent increased occurrence of thromboembolic events, and certainly an increased occurrence of non-traumatic haemorrhage [2, 5].

### Genetics of HHT

Genetic heterogeneity has been demonstrated with identification of five loci to date, of which HHT1, HHT2 and JP-HHT are the most well described. HHT1 is caused by mutations in Endoglin (*ENG*) (OMIM 131195) and HHT2 by mutations in *ACVRL1* (Activin A receptor, type II-like 1) (OMIM 601284). The HHT1 and HHT2 phenotypes share the amount of telangiectatic lesions, epistaxis and GI bleeding evenly. But HHT1 patients have a high prevalence of PAVMs while HHT2 have a higher prevalence of HAVMs. A phenotype consisting of HHT and Juvenile Polyposis Syndrome (JP-HHT) was described in 2004 and is due to mutations in *SMAD4* (OMIM 600993) [20].

HHT is a clinical diagnosis, according to the Curaçao criteria [21]. In approximately 85% of the HHT patients a mutation in either *ENG* or *ACVRL1* can be identified at mutation analysis, and 2–3% of patients have a mutation in *SMAD4* [12].

### Cancer and HHT

Mutations in the three HHT causing genes cause alterations in the TGF-β pathway which is responsible for angiogenesis. Disturbance of angiogenesis causes development of telangiectatic lesions, and one may speculate if disturbances of angiogenesis also alter the occurrence of cancer. Molecular biology studies have indicated that at least regarding Endoglin (HHT1) reduced levels may reflect reduced tumour angiogenesis [22]. In the past years there have been some indications that HHT patients may have superior cancer-survival [23] and may even have a lower risk of cancer [24]. On the other hand, recently the occurrence of cancer was reported equal to controls in a large study by Duarte et al. [25].

### Epidemiology of HHT

HHT occurs with wide ethnic and geographic distribution. The reported Danish prevalence is 1/6500 [1] which is slightly higher compared to other European countries. However, during the last 20 years increasing prevalence has been reported from many HHT-center’s [26, 27]. The higher prevalence is thought to be due to increased awareness and inclusion of the subset of patients with minor symptoms rather than truly increasing incidence.

### Aim

To estimate survival in our group of HHT patients and compare the survival with age- and sex-matched controls.

To see if there are marked differences in causes of death between the group of HHT patients and in the group of controls.

To investigate cancer prevalence among HHT patient, and compare the differences in cancer prevalence between HHT patients and controls.

## Methods

The identification of HHT patients and controls has been described previously [8, 28]. Basically all known HHT patients and all first-degree relatives of HHT patients living in the County of Fyn were invited for clinical examination for signs of HHT. Hereby, we identified all HHT patients who fulfilled the clinical criteria of HHT, regardless of their need for medical care. A total of 73 HHT patients who fulfilled the clinical criteria of HHT 1.1.1995 and lived in the County of Fyn were included in the follow-up study. The controls consisted of 218 inhabitants in the County of Fyn the 1.1.1995; they were age- and sex-matched. Three controls were chosen randomly for each HHT patient when the HHT cohort was identified in 1995, one control was lost due to registration failure. As the controls were not contacted and the HHT patients (cases) were only seen at entry and if they later were in need of medical care at the HHT-center. Data concerning smoking habits, lifestyle and comorbidity were not available.

In 2015 we performed a register search in the Danish Population Registry using the civil registration number of the HHT cases and of the controls. By this method all deaths and causes of death among patients and controls as well as emigrations in the study period were recorded. Causes of death were retrieved from the Danish Cause of Death Registry.

In order to further evaluate the causes of death among patients and controls, a thorough assessment of the ICD-10 classifications was performed and all diagnoses were, from a clinical point of view, categorized as HHT- or not HHT-related. HHT related diagnoses were based on the reported increased occurrence of bacterial infections, risk of thromboembolic events, and hemorrhages among HHT patients [2, 14, 29]. The HHT-related diagnoses were grouped as seen in Additional files 1: Table S2. Overview, and Additional files 2: Table S3 including all diagnosis and all codes.

Regarding cancer, first event of cancer was recorded both in the HHT group and in the control group. These data were retrieved from the Danish Patient Registry. The results where validated by extracting data from the Danish Cancer Registry. Non-melanoma skin cancers were omitted from the analysis, as this diagnostic group are less likely to be complete and accurate.

### Setting

The Danish HHT-center was established at Odense University Hospital in 1995 on the basis of several years with ongoing epidemiological studies in the County of Fyn [30]. Since 1.1.1995 all patients seen at the Danish HHT-center have been clinically evaluated concerning manifestations of HHT. All participants have been offered genetic testing. The HHT-center receives referral patients with HHT from all parts of Denmark. However, concerning the County of Fyn, we have been able to include and follow all patients from Fyn.

The County of Fyn, Denmark, is a geographical well defined area with access to Odense University Hospital. All inhabitants in Denmark are served by a free public health care system, in which all hospital contacts are registered. Data concerning prevalence of HHT disease has previously been published [1, 31].

### Clinical evaluation of patients

The HHT patients and their first degree relatives were at inclusion evaluated regarding symptoms and signs of HHT. They were interviewed regarding epistaxis and other HHT manifestations, and they underwent a clinical examination looking for telangiectatic lesions with focus on characteristic sites, being nasal mucosa, oral mucosa, lips, skin of face, conjunctiva, and fingertips. Including the first degree relatives in the HHT screening, allowed us to identify HHT cases with very few symptoms. Only HHT cases with definite HHT were included in the follow-up. The inclusion criteria at that time were: presence of multiple, at least 15, telangiectatic lesions and either a positive family history or recurrent episodes of bleeding [28]. In all HHT cases a history concerning neurological symptoms were taken and screening for PAVM was offered in order to perform embolization if necessary. Screening for CAVM was only offered if the patient had experienced neurological symptoms or deficits. Screening for HAVM was not performed routinely. In all HHT families genetic counselling and mutation diagnostics were offered. The controls were not clinical evaluated, but only identified in a register search, having the same age and sex as the included HHT patients and being alive and having address in the County of Fyn 1.1.1995.

### Mutation analysis

Genomic DNA was isolated from peripheral leukocytes. All exons and exon-intron boundaries of *ENG* (RefSeq: NM_001114753.1), *ACVRL1* (RefSeq: NM_000020.2), and *SMAD4* (RefSeq: NM_005359.5) were analysed by bi-directional sequencing and Multiplex Ligation-dependent Probe Amplification (MLPA) analysis, as described previously [12]. Before 2007, our laboratory performed DGGE (denaturing gradient gel electrophoresis) and DHPLC (Denaturing High Pressure Liquid Chromatography) analysis of *ENG* and *ACVRL1*, with sequencing of relevant exons as described in detail in other papers [8, 32]. All samples, without an identified pathogenic mutation by this approach, were re-evaluated in 2010–2012 with Sanger sequencing and MLPA analyses of the three HHT causative genes [12]. None of the patients had mutations in *SMAD4*.

### Danish population registries as data sources

Denmark has an array of nationwide registries which are continuously updated and provide excellent opportunities for register-based research. Danish nationwide registries can be accurately linked at the individual level using the unique civil registration numbers assigned to all Danish residents at birth or upon immigration [33]. Furthermore, the highly organized structure of the tax-supported Danish Health Service [34], which provides free of charge health care to all citizens of the country regardless of income, provides a favorable setting for the type of studies included in this paper. To conduct the studies presented in this manuscript we collected data from four nationwide registries, all containing prospectively collected data.

The Danish Civil Registration System (Person Registry) was established in 1968 and contains continuously updated information on vital status, migration and residency. The registry is based on the civil registration number [33]. Data in this registry was updated in the full research period.

The Danish Cancer Registry (Cancer Registry) was founded in 1942. It is mandatory to report incident cases of cancer and selected tumors to the Cancer Registry. Cancer diagnoses are recorded according to the International Classification of Diseases, version 10 (ICD-10) [35]. We used data from this registry updated until 1 January 2013.

The Danish Register of Causes of Death covers all deaths among citizens dying in Denmark since 1875. The register is computerized and it includes all the diagnoses recorded at the death certificate as the evaluated cause of death. Since 1994 only diagnoses according to ICD-10 classification have been used [36]. We used data from this registry updated until 1 January 2014.

The Danish Patient Registry includes information on all procedures and treatments, together with all associated diagnoses, for all admissions and outpatient contacts (including visit to emergency rooms) managed at Danish hospitals (public as well as private). We used data from this registry updated as of 31 December 2014.

### Statistics

We conducted survival analysis with follow-up as time scale. Kaplan-Meier curves with 95% confidence bands were calculated for HHT cases versus controls. We used the same populations which were evaluated for 90-month mortality [8]. Furthermore, hazard ratios between cases and controls and between subtypes were estimated with Cox regression, adjusting for sex and age at start of follow-up and checking fulfillment of the proportional hazards assumption. Using knowledge of cancer diagnosis we also performed Kaplan-Meier survival curves regarding cancer-free survival. Concerning causes of death we used descriptive statistics comparing causes of death in the group of HHT patients and controls.

## Results

All 73 HHT patients were followed for 20 years, as well as 218 of 219 controls (one control was lost due to registration failure). Phenotype and genotype characteristics of the HHT patients are shown in Table 1.

**Table 1:** Pheno- and genotype distribution among HHT patients

	All HHT patients	Male	Female
Number	73	34	39
Age 1 January 1995 (range)	53.8 (14–88)	51.5 (18–88)	55.5 (14–87)
PAVM diagnosed at CT or Pulmonary angiography	23	9	14
HHT1	38	18	20
HHT2	24	14	10
JP-HHT	0	0	0
Unknown type	11	2	9
Died in the follow-up period	32	13	19
HHT stated as cause of death	6	3	3
Cancer stated as cause of death	3	1	2
Other HHT related diagnosis stated as cause of death	10	6	4

*Abbreviations*: *PAVM* Pulmonary arteriovenous malformations, *HHT1* Hereditary Hemorrhagic Telangiectasia type 1, *HHT2* Hereditary Hemorrhagic Telangiectasia type 2, *HHT- JP* Hereditary Hemorrhagic Telangiectasia and Juvenile Polyposis

**Fig. 2:**
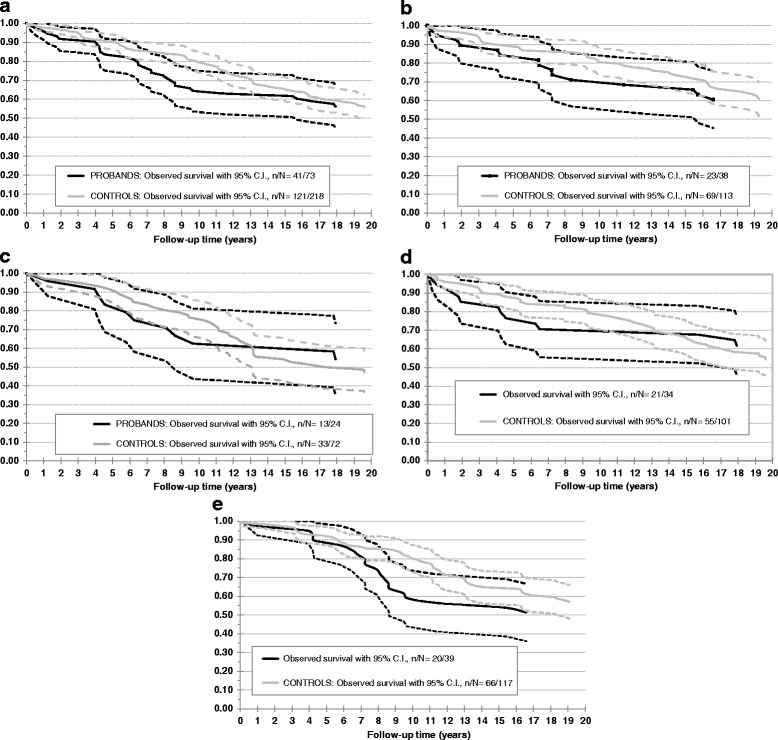
Crude Survival: **a** All HHT patients and controls **b** HHT1 patients and controls **c** HHT2 patients and controls. **d** Female HHT patients and controls, **e** Male HHT patients and controls

Crude survival showed that 41 (56%) HHT patients and 121 (56%) controls survived the study period. Survival curves are shown in (Fig. 2 a–e). We expected to identify a higher mortality among HHT patients than among controls, but there was no significant increased mortality, and the curves evened out during the full follow-up period. Neither a logrank test (*p* = 0.10) nor a Cox regression adjusting for age and sex (HR = 1.27, *p* = 0.24) found any significant differences in survival between cases and controls. Subdividing according to the genetic HHT subtype (Fig. 2b and c) did not reveal increased mortality among either HHT1 or HHT2 patients and neither a logrank test (*p* = 0.17) nor Cox regression adjusted for age and sex (HR = 0.46, *p* = 0.066) found any significant differences in survival between HHT1 and HHT2. Subdividing according to sex (Fig. 2d and e) did not reveal increased mortality in either sex. The number of patients with cancer was too small to make any significant change in the survival curves (cancer survival curves not shown).

According to the Danish Patient Registry only 4 (5.5%) of HHT patients were diagnosed with cancer during the study period compared to 38 (17.4%) of the controls (*p* = 0.012 using Fisher’s exact test). These results were validated using the Cancer Registry. Here we found the same 4 HHT patients with a cancer diagnosis but we only identified 32 of the 38 controls diagnosed with cancer (14.6%). The difference in cancer prevalence between HHT patients and controls was still significant (*p* = 0.04 using Fisher’s exact test).

## Discussion

In this study we were able to include all HHT patients from a well-defined geographical area. Patients were included regardless of severity of disease as long as they fulfilled the diagnostic criteria of HHT. Even though this was done before the Curacao criteria were established, subsequent evaluation has demonstrated that all patients fulfilled the Curacao criteria [21]. The problem with referral bias was reduced substantially as HHT cases with few symptoms, diagnosed through screening of first degree relatives, also were included. However subclinical cases of HHT may have been missed for inclusion in the case group. A well-defined control group was identified using sex, birthday and County of Fyn address as matching. The control group was not clinical examined, and although the extremely low prevalence of HHT makes it very unlikely, subclinical cases of HHT in the control group cannot fully be excluded. In this respect it should be noted that during the 20 years follow-up period no diagnosis of HHT was registered in the control group.

The possibility of particular associations between cancer and HHT still needs further investigation. There is a risk of confounding in the present study. We lack information on smoking habits as well as lifestyle factors in both patients and controls, and it is possible that HHT patients due to their chronic disease adapt to a healthier life style in order to cope better with their HHT. The majority of cancer cases among controls were pulmonary, making possible differences in smoking habits of outmost importance. The explanation for difference in number of cancer cases among controls using the patient registry versus the cancer registry, is that the cancer registry was not updated in the period of 2013–15. A recent study [25] indicated that the incidence of cancer is the same in HHT patients compared to the background population although the same authors previously have found a better survival of cancer among HHT patients compared to controls. We speculate, as others [24], whether these important results reflect a change in the expression of genes belonging to the TGF-β pathway influencing angiogenesis which may reduce the risk of cancer and/or increase survival of cancer. Our results support this, but further studies of the angiogenesis among HHT patients are needed before we can reach a firm conclusion.

We have previously reported a slightly increased mortality among HHT patients in the County of Fyn1974-95 [1]. We further did observe a slight trend towards increased mortality in the County of Fyn 1995–2002 in the current study group after 90 months’ observation. With extended observation period the difference between HHT patients and controls disappeared. This may be a random effect but it may also be a beneficial consequence of the establishment of the HHT center. Although we have included all HHT patients in a geographical area independent of severity of disease, and thereby have tried to reduce selection bias, we cannot fully exclude that there may have been some young HHT patients dying of complication to the disease before they were diagnosed with HHT. Nevertheless, we did ask for information on siblings and children, when the HHT patients were included, and we have no suspicion that dying young has been common in the families. To fully omit the risk of selection bias, all newborns of HHT parents should be genetic tested and followed for a lifetime, comparing survival of siblings with and without HHT. This is, however, not realistic and we believe the present study gives the most current and relevant information for HHT patients and care takers on HHT survival. An explanation for the survival curves that evens out could be that the HHT patients may be protected against some of the diseases related to older age, e.g. cancer and heart disease, but this is speculative and may also be blurred by lifestyle differences.

Other authors have reported increased mortality among HHT patients. de Gussem et al. [7] reported a reduced life expectancy in a largely unscreened and untreated population of HHT patient (parents of HHT patients). Donaldsen et al. [5] in their register study identified a substantial risk of serious neurological and hemorrhagic complications and an increased mortality, using register data on patients registered with the HHT diagnosis at the GP in UK, thus excluding subclinical HHT cases without need of medical care. Further information on the degree of surveillance of HHT complications and the way of managing the HHT disease is not provided. Consequently, selection bias is likely as also stated by the authors [27]. Our population was ascertained using family investigations implying that all cases regardless of severity and need of medical care were identified and included. As expected for an autosomal dominant trait we found equal prevalence in men and woman, hence eliminating gender-related bias. All HHT patients underwent clinical examination by the same investigator when included in the study. Furthermore, all patients were informed about HHT and bleedings. Most importantly, the HHT- patients were offered screening and direct access to treatment for the most dangerous complication (PAVM) at the Odense University Hospital. When indicated the patients were also offered treatment for other individual manifestations, including laser therapy of the nose, iron treatment, endoscopic treatment of the GI tract, embolization of PAVM, prophylactic antibiotics, and blood transfusion. Taken together, this is assumed to reduce the risk of severe complications such as: cerebral abscess, severe bleeding, stroke, TIA and other HHT related complications.

## Conclusion

We have enrolled HHT patients at an HHT-center with highly skilled researchers and specialized treatment modalities. Among the 73 HHT patients who were followed for 20 years there was no increased mortality. This may be partly due to screening for and treatment of PAVMs, as well as information on and treatment of other HHT manifestations (epistaxis and GI-bleeding) at a dedicated HHT-center. Our data also suggests that the benefit of having HHT might be a reduced prevalence of cancer.

## Additional files

Additional file 1: Table S2. Online only. Grouping of ICD-10 classification according to HHT relevance Overview for details see Additional file 2: **Table S3.** online only. (DOCX 14 kb)
Additional file 2: Table S3. Online only full list of HHT relevans. (DOCX 114 kb)

## Abbreviation

ACVRL1: Activin A receptor type II-like 1
CA: Cerebral abscess
CAVM: Cerebral arteriovenous malformations
ENG: Endoglin
GI: Gastro intestinal
HAVM: Hepatic arteriovenous malformations
HHT: Hereditary hemorrhagic telangiectasia
JP-HHT: Juvenile polyposis syndrome and HHT
MLPA: Multiplex Ligation-dependent Probe Amplification
PAVM: Pulmonary arteriovenous malformations
