# Supplementary material for: 20-year follow-up study of Danish HHT patients—survival and causes of death
Source: Orphanet J Rare Dis. 2016 Nov 22;11:157. doi: 10.1186/s13023-016-0533-9 (PMC5120428; doi:10.1186/s13023-016-0533-9)
Supplement: Additional file 1: Table S2. — Online only. Grouping of ICD-10 classification according to HHT relevance Overview for details see Additional file 2: Table S3. online only. (DOCX 14 kb) [file 13023_2016_533_MOESM1_ESM.docx]

Table 2 Online only

Grouping of ICD-10 classification according to HHT relevance Overview for details see Table 3 online only

PAVMs: Pulmonary arterio-venous malformations. CNS: Central nervous system GI: gastro intestinal. HHT: Hereditary Hemorrhagic Telangiectasia.

| **Groups of Diagnoses** | **Subgroups of Diagnoses** |
| --- | --- |
| **Bacterial infections** | **Infections in the CNS** |
|  | **Infections in joints and bones** |
|  | **Infections in lower airways** |
|  | **Infections in wounds and skin** |
|  | **Sepsis** |
|  | **Remaining infections** |
| **Thromboembolic conditions** | **Thromboembolisms in the CNS** |
|  | **Remaining Thromboembolisms** |
| **Non-traumatic bleedings** | **Bleedings in the CNS** |
|  | **Bleedings in the GI tract** |
|  | **Epistaxis** |
|  | **Remaining bleedings** |
|  | **Possible bleedings** |
| **Other vascular conditions** | **Other vascular conditions in the CNS** |
|  | **Remaining other vascular conditions** |
| **PAVMs** |  |
| **Cancer** |  |
| **Primary HHT** |  |
