# Supplementary material for: 20-year follow-up study of Danish HHT patients—survival and causes of death
Source: Orphanet J Rare Dis. 2016 Nov 22;11:157. doi: 10.1186/s13023-016-0533-9 (PMC5120428; doi:10.1186/s13023-016-0533-9)
Supplement: Additional file 2: Table S3. — Online only full list of HHT relevans. (DOCX 114 kb) [file 13023_2016_533_MOESM2_ESM.docx]

Tabel 3 Online only full list of HHT relevans

| **ICD10_code** | **HHTGroups of Diagnoses** | **HHT Subgroups of Diagnoses** | **Code Text (Danish only)** |
| --- | --- | --- | --- |
| A00 | Bacterial infections | Remaining infections | Kolera |
| A000 | Bacterial infections | Remaining infections | Kolera forårsaget af vibrio cholerae (klassisk kolera) |
| A001 | Bacterial infections | Remaining infections | Kolera forårsaget af Vibrio cholerae eltor |
| A009 | Bacterial infections | Remaining infections | Kolera uden specifikation |
| A01 | Bacterial infections | Remaining infections | Tyfus og paratyfus |
| A010 | Bacterial infections | Remaining infections | Tyfus |
| A011 | Bacterial infections | Remaining infections | Paratyfus A |
| A012 | Bacterial infections | Remaining infections | Paratyfus B |
| A013 | Bacterial infections | Remaining infections | Paratyfus C |
| A014 | Bacterial infections | Remaining infections | Paratyfus uden specifikation |
| A02 | Bacterial infections | Remaining infections | Salmonella infektioner, andre |
| A020 | Bacterial infections | Remaining infections | Salmonella enteritis |
| A021 | Bacterial infections | Sepsis | Salmonella sepsis |
| A022 | Bacterial infections | Remaining infections | Salmonella infektion, lokaliseret |
| A022A | Bacterial infections | Remaining infections | Salmonella arthritis |
| A022B | Bacterial infections | Remaining infections | Salmonella infektion, renal tubulo-interstitiel |
| A022C | Bacterial infections | Infections in the CNS | Salmonella meningitis |
| A022D | Bacterial infections | Infections in joints and bones | Salmonella osteomyelitis |
| A022E | Bacterial infections | Infections in lower airways | Salmonella pneumoni |
| A028 | Bacterial infections | Remaining infections | Salmonella infektion, anden specificeret |
| A029 | Bacterial infections | Remaining infections | Salmonella infektion uden specifikation |
| A03 | Bacterial infections | Remaining infections | Shigellose |
| A030 | Bacterial infections | Remaining infections | Bacillær dysenteri forårsaget af Shigella dysenteriae |
| A031 | Bacterial infections | Remaining infections | Bacillær dysenteri forårsaget af Shigella Flexneri |
| A032 | Bacterial infections | Remaining infections | Bacillær dysenteri forårsaget af Shigella Boydi |
| A033 | Bacterial infections | Remaining infections | Bacillær dysenteri forårsaget af Shigella Sonnei |
| A038 | Bacterial infections | Remaining infections | Bacillær dysenteri, anden |
| A039 | Bacterial infections | Remaining infections | Bacillær dysenteri uden specifikation |
| A04 | Bacterial infections | Remaining infections | Tarminfektioner, andre bakterielle |
| A040 | Bacterial infections | Remaining infections | Enteritis forårsaget af enteropatogen E coli infektion |
| A041 | Bacterial infections | Remaining infections | Enteritis forårsaget af enterotoksisk E coli infektion |
| A042 | Bacterial infections | Remaining infections | Enteritis forårsaget af enteroinvasiv E coli infektion |
| A043 | Bacterial infections | Remaining infections | Enteritis forårsaget af enterohæmoragisk E coli infektion |
| A044 | Bacterial infections | Remaining infections | Escherichia coli, anden tarminfektion |
| A045 | Bacterial infections | Remaining infections | Campylobacter enteritis |
| A046 | Bacterial infections | Remaining infections | Enteritis forårsaget af Yersinia enterocolitica |
| A047 | Bacterial infections | Remaining infections | Enterocolitis forårsaget af Clostridium difficile |
| A048 | Bacterial infections | Remaining infections | Enteritis, anden specificeret bakteriel |
| A049 | Bacterial infections | Remaining infections | Enteritis forårsaget af bakterier uden specifikation |
| A05 | Bacterial infections | Remaining infections | Alimentære bakterielle intoksikationer, andre |
| A050 | Bacterial infections | Remaining infections | Alimentær stafylokok infektion |
| A051 | Bacterial infections | Remaining infections | Botulismus |
| A052 | Bacterial infections | Remaining infections | Alimentær intoksikation m Clostridium perfringens toksin |
| A053 | Bacterial infections | Remaining infections | Alimentær intoksikation m Vibrio parahaemolyticus |
| A054 | Bacterial infections | Remaining infections | Alimentær intoksikation m Bacillus cereus |
| A058 | Bacterial infections | Remaining infections | Alimentære bacillære intoksikationer, andre |
| A059 | Bacterial infections | Remaining infections | Alimentær bacillær intoksikation uden specifikation |
| A085 | Bacterial infections | Remaining infections | Infektion i tarm, anden specificeret |
| A09 | Bacterial infections | Remaining infections | Diarré og gastroenteritis af infektiøs oprindelse |
| A090 | Bacterial infections | Remaining infections | An. og uspec gastroenteritis og colitis af infektiøs oprind. |
| A099 | Bacterial infections | Remaining infections | Diarré og gastroenteritis af infektiøs oprindelse |
| A15 | Bacterial infections | Infections in lower airways | Tuberkulose i åndedrætsorganer, bakt og hist verificeret |
| A150 | Bacterial infections | Infections in lower airways | Lungetuberkulose, bakt verificeret u angivelse af metode |
| A150A | Bacterial infections | Infections in lower airways | Bronchiectasis tuberculosa, bakteriologisk verificeret |
| A150B | Bacterial infections | Infections in lower airways | Fibrosis pulmonis tuberculosa, bakteriologisk verificeret |
| A150C | Bacterial infections | Infections in lower airways | Pneumonia tuberculosa, bakteriologisk verificeret |
| A150D | Bacterial infections | Remaining infections | Pneumothorax tuberculosa, bakteriologisk verificeret |
| A151 | Bacterial infections | Infections in lower airways | Lungetuberkulose, verificeret ved dyrkning alene |
| A152 | Bacterial infections | Infections in lower airways | Lungetuberkulose, histologisk verificeret |
| A153 | Bacterial infections | Infections in lower airways | Lungetuberkulose, verificeret ved ikke angivet metode |
| A154 | Bacterial infections | Remaining infections | Tuberkulose i intratorakale lymfekirtler, bakt/hist verif |
| A154A | Bacterial infections | Remaining infections | Tuberculosis lymphonodorum hili, bakt og hist verificeret |
| A154B | Bacterial infections | Remaining infections | Tuberculosis lymphonodorum mediast, bakt og hist verificere |
| A154C | Bacterial infections | Remaining infections | Tuberculosis lymphonodorum tracheobronch, bakt og hist veri |
| A155 | Bacterial infections | Infections in lower airways | Tuberkulose i strube luftrør og bronkier, bakt/hist verif |
| A155A | Bacterial infections | Infections in lower airways | Tuberculosis bronchi, bakt og hist verificeret |
| A155B | Bacterial infections | Remaining infections | Tuberculosis glottidis, bakt og hist verificeret |
| A155C | Bacterial infections | Remaining infections | Tuberculosis laryngis, bakt og hist verificeret |
| A155D | Bacterial infections | Infections in lower airways | Tuberculosis tracheae, bakt og hist verificeret |
| A156 | Bacterial infections | Infections in lower airways | Tuberkulose lungehindebetændelse, bakt/hist verificeret |
| A156A | Bacterial infections | Infections in lower airways | Empyema pleurae tuberculosa, bakt og hist verificeret |
| A157 | Bacterial infections | Remaining infections | Prim tuberkulose i åndedrætsorg bakt eller hist verif |
| A158 | Bacterial infections | Remaining infections | Tuberkulose i andre åndedrætsorganer, bakt og hist verif |
| A158A | Bacterial infections | Remaining infections | Tuberculosis mediastini, bakt og hist verificeret |
| A158B | Bacterial infections | Remaining infections | Tuberculosis nasi, bakt og hist verificeret |
| A158C | Bacterial infections | Remaining infections | Tuberculosis nasopharyngei, bakt og hist verificeret |
| A159 | Bacterial infections | Remaining infections | Tuberkulose i åndedrætsorg u spec, bakt/hist verificeret |
| A16 | Bacterial infections | Remaining infections | Tuberkulose i åndedrætsorg ikke bakt eller hist verif |
| A160 | Bacterial infections | Infections in lower airways | Lungetuberkulose, bakt/hist negativ |
| A160A | Bacterial infections | Infections in lower airways | Bronchiectasia tuberculosa, bakt og hist negativ |
| A160B | Bacterial infections | Infections in lower airways | Fibrosis pulmonis tuberculosa, bakt og hist negativ |
| A160C | Bacterial infections | Infections in lower airways | Pneumonia tuberculosa, bakt og hist negativ |
| A161 | Bacterial infections | Infections in lower airways | Lungetuberkulose, bakt/hist undersøgelse ikke foretaget |
| A162 | Bacterial infections | Infections in lower airways | Lungetuberkulose u angivelse af bakt eller hist verif |
| A162A | Bacterial infections | Infections in lower airways | Fibrosis pulmonis tub u spec uden ang af specifikation |
| A162B | Bacterial infections | Infections in lower airways | Pneumonia tuberculosa u spec u ang af bakt ell hist verif |
| A162D | Bacterial infections | Infections in lower airways | Tuberculosis pulm u spec u ang af verifikationsmåde |
| A163 | Bacterial infections | Remaining infections | Tuberkulose intrator lymfekirt u ang af bakt/hist verif |
| A164 | Bacterial infections | Remaining infections | Tuberkulose i strube luftrør og bronk u bakt/hist verif |
| A164A | Bacterial infections | Remaining infections | Tuberculosis bronchi uden angivelse af verifikationmåde |
| A164B | Bacterial infections | Remaining infections | Tuberculosis glottidis uden angivelse af verifikationsmåde |
| A164C | Bacterial infections | Remaining infections | Tuberculosis laryngis uden angivelse af verifikationsmåde |
| A164D | Bacterial infections | Infections in lower airways | Tuberculosis tracheae uden angivelse af verifikationsmåde |
| A165 | Bacterial infections | Infections in lower airways | Tuberkulose lungehindebet u ang af bakt/hist verif |
| A165A | Bacterial infections | Infections in lower airways | Empyema pleurae tuberculosa uden ang af verifikationmåde |
| A167 | Bacterial infections | Remaining infections | Prim tuberkulose i åndedrætsorg uden bakt ell hist verif |
| A168 | Bacterial infections | Remaining infections | Anden tuberkulose i åndedrætsorg u bakt/hist verificeret |
| A168A | Bacterial infections | Remaining infections | Tuberculosis mediastini uden angivelse af verifikationsmåde |
| A168B | Bacterial infections | Remaining infections | Tuberculosis nasi uden angivelse af verifikationsmåde |
| A168C | Bacterial infections | Remaining infections | Tuberculosis sinus nasi uden angivelse af verifikationsmåde |
| A169 | Bacterial infections | Remaining infections | Tuberkulose i åndedrætsorg uden spec u bakt/hist verif |
| A17 | Bacterial infections | Remaining infections | Tuberkulose i nervesystemet |
| A170 | Bacterial infections | Infections in the CNS | Meningoencephalitis tuberculosa |
| A170A | Bacterial infections | Infections in the CNS | Tuberkuløs meningitis |
| A171 | Bacterial infections | Infections in the CNS | Tuberculoma meningum |
| A178 | Bacterial infections | Infections in the CNS | Tuberkulose i nervesystemet, anden |
| A178A | Bacterial infections | Infections in the CNS | Abscessus cerebri tuberculosus |
| A178B | Bacterial infections | Infections in the CNS | Neuritis tuberculosa |
| A178C | Bacterial infections | Infections in the CNS | Tuberculoma medullae spinalis |
| A178D | Bacterial infections | Infections in the CNS | Tuberculosis cerebri |
| A178E | Bacterial infections | Infections in the CNS | Tuberculosis medullae spinalis |
| A179 | Bacterial infections | Infections in the CNS | Tuberkulose i nervesystemet uden specifikation |
| A18 | Bacterial infections | Remaining infections | Tuberkulose i andre organer |
| A180 | Bacterial infections | Infections in joints and bones | Tuberkulose i knogler og led |
| A180A | Bacterial infections | Infections in joints and bones | Necrosis ossis tuberculosa |
| A180B | Bacterial infections | Infections in joints and bones | Ostitis tuberculosa |
| A180C | Bacterial infections | Remaining infections | Tendovaginitis tuberculosa |
| A180D | Bacterial infections | Infections in joints and bones | Tuberculosis articuli |
| A180E | Bacterial infections | Infections in joints and bones | Tuberculosis columnae vertebralis |
| A180F | Bacterial infections | Infections in joints and bones | Tuberculosis coxae |
| A180G | Bacterial infections | Infections in joints and bones | Tuberculosis genus |
| A181 | Bacterial infections | Remaining infections | Tuberkulose i urinveje og kønsorganer |
| A181A | Bacterial infections | Remaining infections | Tuberculosis cervicis uteri |
| A181B | Bacterial infections | Remaining infections | Tuberculosis organorum genitalium feminae |
| A181C | Bacterial infections | Remaining infections | Tuberculosis organorum genitalium viri |
| A181D | Bacterial infections | Remaining infections | Tuberculosis renis |
| A181E | Bacterial infections | Remaining infections | Tuberculosis ureteris |
| A181F | Bacterial infections | Remaining infections | Tuberculosis vesicae urinariae |
| A182 | Bacterial infections | Remaining infections | Tuberkulose i perifere lymfekirtler |
| A183 | Bacterial infections | Remaining infections | Tuberkulose i tarm bughinde og mesenteriale lymfekirtler |
| A183A | Bacterial infections | Remaining infections | Enteritis tuberculosa |
| A183B | Bacterial infections | Remaining infections | Tuberculosis intestini |
| A183C | Bacterial infections | Remaining infections | Tuberculosis lymphoglandulae mesenterii |
| A183D | Bacterial infections | Remaining infections | Tuberculosis mesenterii |
| A183E | Bacterial infections | Remaining infections | Tuberculosis peritonei |
| A183F | Bacterial infections | Remaining infections | Tuberkulose i retroperitoneale lymfeknuder |
| A183H | Bacterial infections | Remaining infections | Tuberkulose i mesenteriale lymfeknuder |
| A184 | Bacterial infections | Remaining infections | Tuberkulose i hud og underhud |
| A184B | Bacterial infections | Infections in wounds and skin | Lupus excedens |
| A184C | Bacterial infections | Infections in wounds and skin | Lupus vulgaris |
| A184D | Bacterial infections | Infections in wounds and skin | Scrofuloderma |
| A184E | Bacterial infections | Infections in wounds and skin | Tuberculosis cutis |
| A184F | Bacterial infections | Infections in wounds and skin | Tuberculosis lichenoides |
| A184G | Bacterial infections | Infections in wounds and skin | Tuberculosis subcutis |
| A184H | Bacterial infections | Infections in wounds and skin | Tuberculosis verrucosa cutis |
| A184I | Bacterial infections | Infections in wounds and skin | Erythema induratum (Bazin) |
| A184J | Bacterial infections | Infections in wounds and skin | Lichen scrofulosorum |
| A185 | Bacterial infections | Remaining infections | Tuberkulose i øje |
| A185A | Bacterial infections | Remaining infections | Chorioretinitis disseminata tuberculosa |
| A185B | Bacterial infections | Remaining infections | Iridocyclitis chronica tuberculosa |
| A185C | Bacterial infections | Remaining infections | Keratitis interstitialis tuberculosa |
| A185D | Bacterial infections | Remaining infections | Keratoconjunctivitis (phlyctaenulosa) tuberculosa |
| A186 | Bacterial infections | Remaining infections | Tuberkulose i øre |
| A187 | Bacterial infections | Remaining infections | Tuberkulose i binyre |
| A187A | Bacterial infections | Remaining infections | Morbus Addisoni tuberculosus |
| A188 | Bacterial infections | Remaining infections | Tuberkulose i andre specificerede organer |
| A188A | Bacterial infections | Remaining infections | Arteritis cerebralis tuberculosa |
| A188B | Bacterial infections | Remaining infections | Erythema induratum |
| A188C | Bacterial infections | Remaining infections | Tuberculosis endocardii |
| A188D | Bacterial infections | Remaining infections | Tuberculosis glandulae thyreoideae |
| A188E | Bacterial infections | Remaining infections | Tuberculosis lienis |
| A188F | Bacterial infections | Remaining infections | Tuberculosis myocardii |
| A188G | Bacterial infections | Remaining infections | Tuberculosis oesophagi |
| A188H | Bacterial infections | Remaining infections | Tuberculosis pericardii |
| A19 | Bacterial infections | Remaining infections | Miliær tuberkulose |
| A190 | Bacterial infections | Remaining infections | Akut miliær tuberkulose, enkelt lokalisation |
| A191 | Bacterial infections | Remaining infections | Akut miliær tuberkulose, flere lokalisationer |
| A192 | Bacterial infections | Remaining infections | Akut miliær tuberkulose uden specifikation |
| A198 | Bacterial infections | Remaining infections | Anden miliær tuberkulose |
| A199 | Bacterial infections | Remaining infections | Miliær tuberkulose uden specifikation |
| A27 | Bacterial infections | Remaining infections | Leptospirose |
| A270 | Bacterial infections | Remaining infections | Weil's sygdom |
| A278 | Bacterial infections | Remaining infections | Anden leptospirose |
| A278A | Bacterial infections | Remaining infections | Leptospirosis Australiae |
| A278B | Bacterial infections | Remaining infections | Leptospirosis Bataviae |
| A278C | Bacterial infections | Remaining infections | Leptospirosis pyrogenes |
| A278D | Bacterial infections | Remaining infections | Leptospirosis Sejrø, Saxkøbing |
| A279 | Bacterial infections | Remaining infections | Leptospirose uden specifikation |
| A28 | Bacterial infections | Remaining infections | Andre bakt dyreoverførte infek, ikke klassificeret ansted |
| A280 | Bacterial infections | Remaining infections | Pasteurellose |
| A281 | Bacterial infections | Remaining infections | Cat-scratch fever |
| A282 | Bacterial infections | Remaining infections | Yersinia infektion uden for tarmkanalen |
| A282A | Bacterial infections | Remaining infections | Arthritis Yersenia enterocolica |
| A282B | Bacterial infections | Remaining infections | Septicaemia Yersinia |
| A282C | Bacterial infections | Remaining infections | Yersinia eksantem |
| A288 | Bacterial infections | Remaining infections | Andre specificerede bakt dyreoverførte infek |
| A289 | Bacterial infections | Remaining infections | Bakteriel dyreoverført infek uden specifikation |
| A31 | Bacterial infections | Remaining infections | Andre mykobakterielle sygdomme |
| A310 | Bacterial infections | Remaining infections | Infektion m mycobacterium i lungerne |
| A310A | Bacterial infections | Remaining infections | Infectio mycobacterica avium pulmonis |
| A310B | Bacterial infections | Remaining infections | Infectio mycobacterica intracellularis (Bacillus Battey) |
| A310C | Bacterial infections | Remaining infections | Infectio mycobacterica kansasii pulmonis |
| A311 | Bacterial infections | Remaining infections | Infektion m mycobacterium i hud |
| A311A | Bacterial infections | Remaining infections | Infectio cutis mycobacterium marinum |
| A311B | Bacterial infections | Remaining infections | Infectio cutis mycobacterium ulcerans |
| A311C | Bacterial infections | Remaining infections | Ulcus Buruli |
| A318 | Bacterial infections | Remaining infections | Anden infek m mycobacterium |
| A319 | Bacterial infections | Remaining infections | Mycobacteriosis atypica uden specifikation |
| A32 | Bacterial infections | Remaining infections | Listeriose |
| A320 | Bacterial infections | Remaining infections | Listeriosis cutanea |
| A321 | Bacterial infections | Infections in the CNS | Meningitis og meningoencefalitis forårsaget af listeria |
| A321A | Bacterial infections | Infections in the CNS | Listeria meningitis |
| A321B | Bacterial infections | Infections in the CNS | Listeria meningoencephalitis |
| A327 | Bacterial infections | Sepsis | Listeria sepsis |
| A328 | Bacterial infections | Remaining infections | Anden Listeriose |
| A328A | Bacterial infections | Remaining infections | Okuloglandulær listeriosis |
| A329 | Bacterial infections | Remaining infections | Listeriose uden specifikation |
| A359 | Bacterial infections | Remaining infections | Anden stivkrampe |
| A36 | Bacterial infections | Remaining infections | Difteri |
| A360 | Bacterial infections | Remaining infections | Tonsillitis diphtherica |
| A360A | Bacterial infections | Remaining infections | Tonsildifteri |
| A361 | Bacterial infections | Remaining infections | Nasopharyngitis diphtherica |
| A361A | Bacterial infections | Remaining infections | Pharyngitis diphtherica |
| A362 | Bacterial infections | Remaining infections | Laryngitis diphtherica |
| A362A | Bacterial infections | Remaining infections | Laryngotracheitis diphtherica |
| A363 | Bacterial infections | Remaining infections | Diphtheria cutanea |
| A368 | Bacterial infections | Remaining infections | Anden difteri |
| A368A | Bacterial infections | Remaining infections | Conjunctivitis diphtherica |
| A368B | Bacterial infections | Remaining infections | Diphtheria nasalis |
| A368C | Bacterial infections | Remaining infections | Myocarditis diphtherica |
| A368D | Bacterial infections | Remaining infections | Polyneuritis diphtherica |
| A369 | Bacterial infections | Remaining infections | Difteri uden specifikation |
| A37 | Bacterial infections | Infections in lower airways | Kighoste |
| A370 | Bacterial infections | Infections in lower airways | Kighoste forårsaget af Bordetella pertussis |
| A371 | Bacterial infections | Infections in lower airways | Kighoste forårsaget af Bordetella parapertussis |
| A378 | Bacterial infections | Infections in lower airways | Kighoste forårsaget af anden spec Bordetella infek |
| A379 | Bacterial infections | Infections in lower airways | Kighoste uden specifikation |
| A38 | Bacterial infections | Remaining infections | Skarlagensfeber |
| A389 | Bacterial infections | Remaining infections | Skarlagensfeber |
| A39 | Bacterial infections | Remaining infections | Infektion m meningokokker |
| A390 | Bacterial infections | Infections in the CNS | Meningitis meningococcica |
| A391 | Bacterial infections | Remaining infections | Waterhouse-Friderichsen's syndrom |
| A392 | Bacterial infections | Remaining infections | Akut meningokokbakteriæmi |
| A392A | Bacterial infections | Sepsis | Meningokokseptikæmi |
| A393 | Bacterial infections | Remaining infections | Kronisk meningokokbakteriæmi |
| A394 | Bacterial infections | Remaining infections | Meningokokbakteriæmi uden specifikation |
| A395 | Bacterial infections | Remaining infections | Hjertesygdom forårsaget af meningokokker |
| A395A | Bacterial infections | Remaining infections | Carditis meningococcica |
| A395B | Bacterial infections | Remaining infections | Endocarditis meningococcica |
| A398 | Bacterial infections | Remaining infections | Anden meningokokinfektion |
| A398A | Bacterial infections | Remaining infections | Arthritis postmeningococcica |
| A398B | Bacterial infections | Remaining infections | Neuritis nervi optici meningococcica |
| A399 | Bacterial infections | Remaining infections | Meningokokinfektion uden specifikation |
| A40 | Bacterial infections | Sepsis | Blodforgiftning forårsaget af streptokokker |
| A400 | Bacterial infections | Sepsis | Septikæmi forårsaget af Streptococcus A |
| A401 | Bacterial infections | Sepsis | Septikæmi forårsaget af Streptococcus B |
| A402 | Bacterial infections | Sepsis | Septikæmi forårsaget af Streptococcus D |
| A403 | Bacterial infections | Sepsis | Septikæmi forårsaget af Streptococcus pneumoniae |
| A408 | Bacterial infections | Sepsis | Septikæmi forårsaget af anden streptokok |
| A409 | Bacterial infections | Sepsis | Septikæmi forårsaget af streptokokker uden specifikation |
| A41 | Bacterial infections | Sepsis | Anden blodforgiftning |
| A410 | Bacterial infections | Sepsis | Septikæmi forårsaget af Staphylococcus aureus |
| A411 | Bacterial infections | Sepsis | Septikæmi forårsaget af anden specificeret stafylokok |
| A411A | Bacterial infections | Sepsis | Septicaemia med coagulase-negative stafylokokker |
| A412 | Bacterial infections | Sepsis | Septikæmi forårsaget af stafylokokker uden specifikation |
| A413 | Bacterial infections | Sepsis | Septikæmi forårsaget af Haemophilus influenzae |
| A414 | Bacterial infections | Sepsis | Septikæmi forårsaget af anaërobe bakterier |
| A415 | Bacterial infections | Sepsis | Septikæmi forårsaget af andre gram-negative organismer |
| A418 | Bacterial infections | Sepsis | Anden septikæmi, som ikke kan klassificeret andetsteds |
| A419 | Bacterial infections | Sepsis | Septikæmi uden specifikation |
| A419A | Bacterial infections | Sepsis | Septisk shock |
| A419B | Bacterial infections | Sepsis | Urosepsis |
| A419C | Bacterial infections | Sepsis | Svær sepsis |
| A42 | Bacterial infections | Remaining infections | Strålesvamp |
| A420 | Bacterial infections | Infections in lower airways | Pulmonal aktinomykose |
| A421 | Bacterial infections | Remaining infections | Abdominal actinomykose |
| A422 | Bacterial infections | Remaining infections | Cervikofacial aktinomykose |
| A427 | Bacterial infections | Sepsis | Septikæmi forårsaget af aktinomycose |
| A428 | Bacterial infections | Remaining infections | Anden strålesvamp |
| A429 | Bacterial infections | Remaining infections | Strålesvamp uden specifikation |
| A43 | Bacterial infections | Remaining infections | Nokardiose |
| A430 | Bacterial infections | Infections in lower airways | Pulmonal nocardiose |
| A431 | Bacterial infections | Infections in wounds and skin | Kutan nocardiose |
| A438 | Bacterial infections | Remaining infections | Anden form for nocardiose |
| A439 | Bacterial infections | Remaining infections | Nokardiose |
| A44 | Bacterial infections | Remaining infections | Bartonellose |
| A440 | Bacterial infections | Remaining infections | Systemisk bartonellose |
| A441 | Bacterial infections | Infections in wounds and skin | Kutan og mukokutan bartonellose |
| A448 | Bacterial infections | Remaining infections | Anden form for bartonellose |
| A449 | Bacterial infections | Remaining infections | Bartonellose |
| A46 | Bacterial infections | Infections in wounds and skin | Rosen |
| A469 | Bacterial infections | Infections in wounds and skin | Erysipelas |
| A48 | Bacterial infections | Remaining infections | Andre bakterielle sygdomme, ikke klassificeret andetsteds |
| A480 | Bacterial infections | Remaining infections | Gasgangræn |
| A481 | Bacterial infections | Remaining infections | Legionærsygdom |
| A482 | Bacterial infections | Remaining infections | Legionærsygdom u lungebetændelse |
| A483 | Bacterial infections | Remaining infections | Toksisk shock syndrom |
| A488 | Bacterial infections | Remaining infections | Andre specificerede bakterielle sygdomme |
| A49 | Bacterial infections | Remaining infections | Bakteriel infektion u angivelse af lokalisation |
| A490 | Bacterial infections | Remaining infections | Stafylokokinfektion uden specifikation |
| A491 | Bacterial infections | Remaining infections | Streptokokinfektion uden specifikation |
| A491A | Bacterial infections | Remaining infections | Perianal streptokokinfektion |
| A492 | Bacterial infections | Remaining infections | Infektion m Haemophilus influenzae |
| A493 | Bacterial infections | Remaining infections | Mycoplasma infektion uden specifikation |
| A498 | Bacterial infections | Remaining infections | Andre bakterielle infektion m ukendt lokalisation |
| A499 | Bacterial infections | Remaining infections | Bakteriel infektion uden specifikation |
| A499A | Bacterial infections | Remaining infections | Bakteriæmi |
| A690 | Bacterial infections | Remaining infections | Mundbetændelse forårsaget af Spirokæte |
| A690A | Bacterial infections | Remaining infections | Cancrum oris |
| A690B | Bacterial infections | Remaining infections | Noma |
| A690C | Bacterial infections | Remaining infections | Stomatitis ulcerosa Vincent |
| A691 | Bacterial infections | Remaining infections | Vincent's infektioner, andre |
| A691A | Bacterial infections | Remaining infections | Angina ulcerosa Vincent |
| A691B | Bacterial infections | Remaining infections | Pharyngitis, spirokætebetinget |
| A691C | Bacterial infections | Remaining infections | Vincent's angina |
| A691E | Bacterial infections | Remaining infections | Stomatitis acuta necroticans ulcerativa |
| A691F | Bacterial infections | Remaining infections | Gingivitis Vincent |
| A691G | Bacterial infections | Remaining infections | Pharyngitis fusospirochaetalis |
| A692 | Bacterial infections | Remaining infections | Lyme's sygdom |
| A692A | Bacterial infections | Infections in wounds and skin | Borrelia acrodermatitis |
| A692B | Bacterial infections | Infections in joints and bones | Borrelia arthritis |
| A692C | Bacterial infections | Remaining infections | Borrelia carditis |
| A692D | Bacterial infections | Remaining infections | Borrelia polyradiculitis |
| A692E | Bacterial infections | Remaining infections | Borreliosis |
| A692F | Bacterial infections | Remaining infections | Erythema chronicum migrans (Borrelia) |
| A692G | Bacterial infections | Remaining infections | Polyradiculitis ved Borrelia infektion |
| A698 | Bacterial infections | Remaining infections | Spirokæte infektioner, andre specificerede |
| A699 | Bacterial infections | Remaining infections | Spirokæte infektion uden specifikation |
| A70 | Bacterial infections | Remaining infections | Chlamydia psittaci infektion |
| A709 | Bacterial infections | Remaining infections | Chlamydia psittaci infektion |
| A709A | Bacterial infections | Remaining infections | Ornithosis |
| A709B | Bacterial infections | Remaining infections | Psittacosis |
| A71 | Bacterial infections | Remaining infections | Ægyptisk øjensygdom |
| A710 | Bacterial infections | Remaining infections | Tidligt stadie af trakom |
| A711 | Bacterial infections | Remaining infections | Aktivt stadie af trakom |
| A719 | Bacterial infections | Remaining infections | Trachoma |
| A74 | Bacterial infections | Remaining infections | Chlamydia infektion, andre sygdomme forårsaget af |
| A740 | Bacterial infections | Remaining infections | Konjunktivitis forårsaget af Chlamydia infekt undt nyfødte |
| A748 | Bacterial infections | Remaining infections | Chlamydia infektion, anden |
| A748A | Bacterial infections | Remaining infections | Peritonitis Chlamydiae |
| A749 | Bacterial infections | Remaining infections | Chlamydia infektion uden specifikation |
| B083 | Bacterial infections | Remaining infections | Erythema infectiosum |
| B902A | Bacterial infections | Remaining infections | Sequelae tuberculosis articulorum |
| B95 | Bacterial infections | Remaining infections | Strepto- og stafylokokker som årsag til sygd i andre kap |
| B950 | Bacterial infections | Remaining infections | Streptokokker, gruppe A, som årsag til sygd i andre kap |
| B951 | Bacterial infections | Remaining infections | Streptokokker, gruppe B, som årsag til sygdom |
| B952 | Bacterial infections | Remaining infections | Streptokokker, gruppe D, som årsag til sygdom |
| B953 | Bacterial infections | Remaining infections | Pneumokokker som årsag til sygd klassificeret i andre kap |
| B954 | Bacterial infections | Remaining infections | Anden streptokok som årsag til sygdom |
| B955 | Bacterial infections | Remaining infections | Streptokokker u spec som årsag til sygd klass i andre kap |
| B956 | Bacterial infections | Remaining infections | Staphylococcus aureus som årsag til sygd klass i andre kap |
| B956A | Bacterial infections | Remaining infections | Staphylococcus aureus methicillin resistent (MRSA) |
| B957 | Bacterial infections | Remaining infections | Anden stafylokok som årsag til sygdom |
| B958 | Bacterial infections | Remaining infections | Stafylokokker u spec som årsag til sygd klass i andre kap |
| B96 | Bacterial infections | Remaining infections | Bakterier, andre som årsag til sygd klass i andre kap |
| B960 | Bacterial infections | Remaining infections | Mycoplasma pneum som årsag til sygd klass i andre kap |
| B961 | Bacterial infections | Remaining infections | Klebsiella pneumoniae som årsag til sygd klass i andre kap |
| B962 | Bacterial infections | Remaining infections | Escherichia coli som årsag til sygd klass i andre kap |
| B963 | Bacterial infections | Remaining infections | Haemophilus influenzae som årsag til sygd klass i andre kap |
| B964 | Bacterial infections | Remaining infections | Proteus som årsag til sygd klass i andre kap |
| B965 | Bacterial infections | Remaining infections | Pseudomonas som årsag til sygd klass i andre kap |
| B966 | Bacterial infections | Remaining infections | Bacillus fragilis som årsag til sygd klass i andre kap |
| B967 | Bacterial infections | Remaining infections | Clostridium perfr som årsag til sygd klass i andre kap |
| B968 | Bacterial infections | Remaining infections | Bakterier, andre som årsag til sygd klass i andre kap |
| B968A | Bacterial infections | Remaining infections | Helicobacter pylori som årsag til sygd klass i andre kap |
| B98 | Bacterial infections | Remaining infections | Andre infektiøse agentia som årsag til sygdom |
| B980 | Bacterial infections | Remaining infections | Helicobacter pylori som årsag til sygdom |
| B981 | Bacterial infections | Remaining infections | Vibrio vulnificus som årsag til sygdom |
| B99 | Bacterial infections | Remaining infections | Andre og ikke specificerede infektiøse sygdomme |
| B999 | Bacterial infections | Remaining infections | Andre og ikke specificerede infektiøse sygdomme |
| C00 | Cancer | Cancer | Kræft i læbe |
| C000 | Cancer | Cancer | Neopl mal labii oris superioris ext |
| C001 | Cancer | Cancer | Neopl mal labii oris inferioris ext |
| C002 | Cancer | Cancer | Neopl mal labii oris oris ext uden specifikation |
| C003 | Cancer | Cancer | Neopl mal labii oris superioris int |
| C004 | Cancer | Cancer | Neopl mal labii oris inferioris int |
| C005 | Cancer | Cancer | Neopl mal labii oris oris int uden specifikation |
| C006 | Cancer | Cancer | Neopl mal commissurae labiorum |
| C008 | Cancer | Cancer | Neopl mal labii oris oris overgribende flere områder |
| C009 | Cancer | Cancer | Neopl mal labii oris oris uden specifikation |
| C01 | Cancer | Cancer | Kræft i basis af tunge |
| C019 | Cancer | Cancer | Neopl mal baseos linguae |
| C02 | Cancer | Cancer | Kræft i andre dele af tunge eller uden specifikation |
| C020 | Cancer | Cancer | Neopl mal dorsale linguae forreste 2/3 |
| C021 | Cancer | Cancer | Neopl mal marginale et apicale linguae |
| C021A | Cancer | Cancer | Neopl mal linguae apicale |
| C021B | Cancer | Cancer | Neopl mal linguae marginale |
| C022 | Cancer | Cancer | Neopl mal ventrale linguae forreste 2/3 |
| C022A | Cancer | Cancer | Neopl mal linguae, frenulum |
| C023 | Cancer | Cancer | Neopl mal linguae forreste 2/3 u nærmere spec |
| C024 | Cancer | Cancer | Neopl mal tonsillae linguae |
| C028 | Cancer | Cancer | Neopl mal linguae overgribende flere regioner |
| C029 | Cancer | Cancer | Neopl mal linguae uden specifikation |
| C03 | Cancer | Cancer | Kræft i tandkød |
| C030 | Cancer | Cancer | Neopl mal gingivae maxillaris |
| C031 | Cancer | Cancer | Neopl mal gingivae mandibularis |
| C039 | Cancer | Cancer | Neopl mal gingivae uden specifikation |
| C04 | Cancer | Cancer | Kræft i mundhulens bund |
| C040 | Cancer | Cancer | Neopl mal baseos oris anterioris |
| C041 | Cancer | Cancer | Neopl mal baseos oris lateralis |
| C048 | Cancer | Cancer | Neopl mal baseos oris overgribende flere regioner |
| C049 | Cancer | Cancer | Neopl mal baseos oris uden specifikation |
| C05 | Cancer | Cancer | Kræft i gane |
| C050 | Cancer | Cancer | Neopl mal palati duri |
| C051 | Cancer | Cancer | Neopl mal palati mollis |
| C052 | Cancer | Cancer | Neopl mal uvulae |
| C058 | Cancer | Cancer | Neopl mal palati overgribende flere regioner |
| C059 | Cancer | Cancer | Neopl mal palati uden specifikation |
| C06 | Cancer | Cancer | Kræft i andre dele af mundhule eller uden specifikation |
| C060 | Cancer | Cancer | Neopl mal mucosae buccae oris |
| C061 | Cancer | Cancer | Neopl mal vestibuli oris |
| C062 | Cancer | Cancer | Neopl mal spatii retrodentalis oris |
| C068 | Cancer | Cancer | Neopl mal oris overgribende flere og ikke spec regioner |
| C069 | Cancer | Cancer | Neopl mal oris uden specifikation |
| C07 | Cancer | Cancer | Kræft i ørespytkirtel |
| C079 | Cancer | Cancer | Neopl mal glandulae parotidis |
| C08 | Cancer | Cancer | Kræft i andre af mundhulens spytkirtler |
| C080 | Cancer | Cancer | Neopl mal glandulae submandibularis |
| C081 | Cancer | Cancer | Neopl mal glandulae sublingualis |
| C088 | Cancer | Cancer | Neopl mal gland saliv maj oris overgrib flere spytkirtler |
| C089 | Cancer | Cancer | Neopl mal glandulae salivariae majoris oris uden specifik |
| C09 | Cancer | Cancer | Kræft i tonsil |
| C090 | Cancer | Cancer | Neopl mal fossae tonsillae |
| C090A | Cancer | Cancer | Neopl mal tonsillae, fossa |
| C090B | Cancer | Cancer | Neopl mal tonsillae, sinus |
| C091 | Cancer | Cancer | Neopl mal arcus palatoglossi sive palatopharyngei |
| C091A | Cancer | Cancer | Neopl mal tonsillae, arcus palatoglossus |
| C091B | Cancer | Cancer | Neopl mal tonsillae, arcus palatopharyngeus |
| C092 | Cancer | Cancer | Neopl mal tonsillae palatinae |
| C098 | Cancer | Cancer | Neopl mal tonsillae overgribende flere regioner |
| C099 | Cancer | Cancer | Neopl mal tonsillae uden specifikation |
| C10 | Cancer | Cancer | Kræft i midterste orale del af svælg |
| C100 | Cancer | Cancer | Neopl mal valleculae epiglotticae |
| C101 | Cancer | Cancer | Neopl mal oropharyngis epiglottis' forflade |
| C102 | Cancer | Cancer | Neopl mal oropharyngis sidevæg |
| C103 | Cancer | Cancer | Neopl mal oropharyngis bagvæg |
| C104 | Cancer | Cancer | Neopl mal cystis sive fistulae branchialis |
| C104A | Cancer | Cancer | Neopl mal oropharyngis, cystis branchialis |
| C104B | Cancer | Cancer | Neopl mal oropharyngis, fistula branchialis |
| C108 | Cancer | Cancer | Neopl mal oropharyngis overgribende flere regioner |
| C109 | Cancer | Cancer | Neopl mal oropharyngis uden specifikation |
| C11 | Cancer | Cancer | Kræft i næsesvælg |
| C110 | Cancer | Cancer | Neopl mal nasopharyngis øvre væg |
| C111 | Cancer | Cancer | Neopl mal nasopharyngis bagvæg |
| C112 | Cancer | Cancer | Neopl mal nasopharyngis sidevæg |
| C113 | Cancer | Cancer | Neopl mal nasopharyngis forvæg |
| C118 | Cancer | Cancer | Neopl mal nasopharyngis overgribende flere regioner |
| C119 | Cancer | Cancer | Neopl mal nasopharyngis uden specifikation |
| C12 | Cancer | Cancer | Kræft i pyriforme sinus |
| C129 | Cancer | Cancer | Neopl mal sinus pyriformis |
| C13 | Cancer | Cancer | Kræft i nedre del af svælg |
| C130 | Cancer | Cancer | Neopl mal regionis postcricoideae |
| C131 | Cancer | Cancer | Neopl mal plicae aryepiglotticae |
| C132 | Cancer | Cancer | Neopl mal hypopharyngis bagvæg |
| C138 | Cancer | Cancer | Neopl mal hypopharyngis overgribende flere regioner |
| C139 | Cancer | Cancer | Neopl mal hypopharyngis uden specifikation |
| C14 | Cancer | Cancer | Kræft m anden lokalisation på læbe, i mundhule og svælg |
| C140 | Cancer | Cancer | Neopl mal pharyngis uden specifikation |
| C141 | Cancer | Cancer | Neopl mal laryngopharyngis |
| C142 | Cancer | Cancer | Neopl mal circuli lymphatici pharyngis Waldeyer |
| C148 | Cancer | Cancer | Neopl mal overgribende læbe, mundhule og svælg |
| C15 | Cancer | Cancer | Kræft i spiserør |
| C150 | Cancer | Cancer | Neopl mal oesophagi pars cervicalis |
| C151 | Cancer | Cancer | Neopl mal oesophagi pars thoracalis |
| C152 | Cancer | Cancer | Neopl mal oesophagi pars abdominalis |
| C153 | Cancer | Cancer | Neopl mal oesophagi øverste trediedel |
| C154 | Cancer | Cancer | Neopl mal oesophagi midterste trediedel |
| C155 | Cancer | Cancer | Neopl mal oesophagi nederste trediedel |
| C158 | Cancer | Cancer | Neopl mal oesophagi overgribende flere regioner |
| C159 | Cancer | Cancer | Neopl mal oesophagi uden specifikation |
| C16 | Cancer | Cancer | Kræft i mavesæk |
| C160 | Cancer | Cancer | Neopl mal cardiae |
| C161 | Cancer | Cancer | Neopl mal fundi ventriculi |
| C162 | Cancer | Cancer | Neopl mal corporis ventriculi |
| C163 | Cancer | Cancer | Neopl mal antri pylorici ventriculi |
| C164 | Cancer | Cancer | Neopl mal pylori |
| C165 | Cancer | Cancer | Neopl mal curvaturae minoris ventriculi |
| C166 | Cancer | Cancer | Neopl mal curvaturae majoris ventriculi |
| C168 | Cancer | Cancer | Neopl mal ventriculi overgribende flere regioner |
| C169 | Cancer | Cancer | Neopl mal ventriculi uden specifikation |
| C17 | Cancer | Cancer | Kræft i tyndtarm |
| C170 | Cancer | Cancer | Neopl mal duodeni |
| C171 | Cancer | Cancer | Neopl mal jejuni |
| C172 | Cancer | Cancer | Neopl mal ilei |
| C173 | Cancer | Cancer | Neopl mal diverticuli Meckeli |
| C178 | Cancer | Cancer | Neopl mal intestini tenuis overgribende flere regioner |
| C179 | Cancer | Cancer | Neopl mal intestini tenuis uden specifikation |
| C18 | Cancer | Cancer | Kræft i tyktarm |
| C180 | Cancer | Cancer | Neopl mal coeci |
| C180A | Cancer | Cancer | Neopl mal valvulae ileocoecalis |
| C181 | Cancer | Cancer | Neopl mal appendicis |
| C182 | Cancer | Cancer | Neopl mal coli ascendentis |
| C183 | Cancer | Cancer | Neopl mal coli flexurae hepaticae |
| C184 | Cancer | Cancer | Neopl mal coli transversi |
| C185 | Cancer | Cancer | Neopl mal coli flexurae lienalis |
| C186 | Cancer | Cancer | Neopl mal coli descendentis |
| C187 | Cancer | Cancer | Neopl mal coli sigmoidei |
| C188 | Cancer | Cancer | Neopl mal coli overgribende flere regioner |
| C188A | Cancer | Cancer | Hereditær non-polypøs kolorektal cancer (HNPCC) |
| C189 | Cancer | Cancer | Neopl mal coli uden specifikation |
| C19 | Cancer | Cancer | Kræft på overgangen mellem tyktarm og endetarm |
| C199 | Cancer | Cancer | Neopl mal rectosigmoidei |
| C20 | Cancer | Cancer | Kræft i endetarm |
| C209 | Cancer | Cancer | Neopl mal recti |
| C21 | Cancer | Cancer | Kræft i anus og analkanalen |
| C210 | Cancer | Cancer | Neopl mal ani uden specifikation |
| C210D | Cancer | Cancer | Neoplasma malignum ani, basocellulært, nodulært |
| C210E | Cancer | Cancer | Neoplasma malignum ani, basocellulært, superficielt |
| C210F | Cancer | Cancer | Neoplasma malignum ani, basocellulært, morphaeatype |
| C210G | Cancer | Cancer | Neoplasma malignum ani, basocellulært, multiplex |
| C210J | Cancer | Cancer | Neoplasma malignum ani, planocellulært, verrukøst (cuniculatum, Buschke-Löwenstein) |
| C210K | Cancer | Cancer | Neoplasma malignum ani, planocellulært, spinocellulært |
| C210L | Cancer | Cancer | Neoplasma malignum ani, planocellulært, Marjorlin/Melaney |
| C210M | Cancer | Cancer | Neoplasma malignum ani, planocellulært, recidivans |
| C210N | Cancer | Cancer | Neoplasma malignum ani, mikrocystisk adneks karcinom |
| C210P | Cancer | Cancer | Neoplasma malignum ani, Merkelcelle karcinom |
| C210Q | Cancer | Cancer | Neoplasma malignum ani, adenoidt cystisk karcinom |
| C210R | Cancer | Cancer | Neoplasma malignum ani, mb. Paget (ekstramammært) |
| C210S | Cancer | Cancer | Neoplasma malignum ani, porokarcinom |
| C210T | Cancer | Cancer | Neoplasma malignum ani, sebaceøst karcinom |
| C210U | Cancer | Cancer | Neoplasma malignum ani, trichillemmalt karcinom |
| C210V | Cancer | Cancer | Neoplasma malignum ani, dermatofibrosarcoma protuberans |
| C210W | Cancer | Cancer | Neoplasma malignum ani, angiosarkom |
| C210Z | Cancer | Cancer | Neoplasma malignum ani, andet |
| C211 | Cancer | Cancer | Neopl mal canalis analis |
| C212 | Cancer | Cancer | Neopl mal zonae cloacogenicae |
| C218 | Cancer | Cancer | Neopl mal ani et canalis analis overgribende flere reg |
| C22 | Cancer | Cancer | Kræft i lever og intrahepatiske galdeveje |
| C220 | Cancer | Cancer | Neopl mal hepatocellulare |
| C221 | Cancer | Cancer | Neopl mal viarum biliarium intrahepaticarum |
| C221A | Cancer | Cancer | Cholangiocarcinoma, intrahepatisk |
| C222 | Cancer | Cancer | Hepatoblastoma |
| C223 | Cancer | Cancer | Angiosarcoma hepatis |
| C224 | Cancer | Cancer | Sarcoma hepatis anden form |
| C227 | Cancer | Cancer | Carcinoma hepatis spec anderledes |
| C229 | Cancer | Cancer | Neopl mal hepatis uden specifikation |
| C23 | Cancer | Cancer | Kræft i galdeblære |
| C239 | Cancer | Cancer | Neopl mal vesicae felleae |
| C24 | Cancer | Cancer | Kræft i andre og ikke spec dele af galdeveje eller uden spe |
| C240 | Cancer | Cancer | Neopl mal viarum biliarium extrahepaticarum |
| C240A | Cancer | Cancer | Neopl mal ductus choledochi |
| C240B | Cancer | Cancer | Neopl mal ductus cystici |
| C240C | Cancer | Cancer | Neopl mal ductus hepatici communis |
| C240D | Cancer | Cancer | Neopl mal ductus hepatici dexter |
| C240E | Cancer | Cancer | Neopl mal ductus hepatici sinister |
| C241 | Cancer | Cancer | Neopl mal papillae Vateri |
| C248 | Cancer | Cancer | Neopl mal overgribende flere regioner i galdeveje |
| C249 | Cancer | Cancer | Neopl mal viarum biliarium uden specifikation |
| C25 | Cancer | Cancer | Kræft i bugspytkirtel |
| C250 | Cancer | Cancer | Neopl mal capitis pancreatis |
| C251 | Cancer | Cancer | Neopl mal corporis pancreatis |
| C252 | Cancer | Cancer | Neopl mal caudae pancreatis |
| C253 | Cancer | Cancer | Neopl mal ductus pancreatici |
| C254 | Cancer | Cancer | Neopl mal insulae Langerhans |
| C257 | Cancer | Cancer | Neopl mal pancreatis andre dele |
| C258 | Cancer | Cancer | Neopl mal pancreatis overgribende flere regioner |
| C259 | Cancer | Cancer | Neopl mal pancreatis uden specifikation |
| C26 | Cancer | Cancer | Kræft i andre og dårligt definerede fordøjelsesorganer |
| C260 | Cancer | Cancer | Neopl mal tractus intestinalis uden specifikation |
| C261 | Cancer | Cancer | Neopl mal lienis |
| C268 | Cancer | Cancer | Neopl mal systematis digestivi overgribende flere regioner |
| C269 | Cancer | Cancer | Neopl mal systematis digestivi uden specifikation |
| C30 | Cancer | Cancer | Kræft i næsehule og mellemøre |
| C300 | Cancer | Cancer | Neopl mal cavi nasi |
| C300A | Cancer | Cancer | Neopl mal nasi, concha |
| C300B | Cancer | Cancer | Neopl mal nasi, septum |
| C300C | Cancer | Cancer | Neopl mal nasi, vestibulum |
| C301 | Cancer | Cancer | Neopl mal auris mediae |
| C301A | Cancer | Cancer | Neopl mal cellulae mastoideae |
| C301B | Cancer | Cancer | Neopl mal tubae auditivae |
| C31 | Cancer | Cancer | Kræft i bihuler |
| C310 | Cancer | Cancer | Neopl mal sinus maxillaris |
| C311 | Cancer | Cancer | Neopl mal sinus ethmoidalis |
| C312 | Cancer | Cancer | Neopl mal sinus frontalis |
| C313 | Cancer | Cancer | Neopl mal sinus sphenoidalis |
| C318 | Cancer | Cancer | Neopl mal sinuum nasi overgribende flere regioner |
| C319 | Cancer | Cancer | Neopl mal sinuum nasi uden specifikation |
| C32 | Cancer | Cancer | Kræft i strubehoved |
| C320 | Cancer | Cancer | Neopl mal glottidis |
| C320A | Cancer | Cancer | Neopl mal plicae vocalis |
| C321 | Cancer | Cancer | Neopl mal laryngis pars supraglottica |
| C321A | Cancer | Cancer | Neopl mal epiglottidis, bagflade |
| C321B | Cancer | Cancer | Neopl mal epiglottidis pars suprahyoidea uden spec |
| C321C | Cancer | Cancer | Neopl mal plicae aryepiglotticae, pars laryngealis |
| C321D | Cancer | Cancer | Neopl mal plicae vestibularis |
| C322 | Cancer | Cancer | Neopl mal laryngis pars subglottica |
| C323 | Cancer | Cancer | Neopl mal cartilaginis laryngis |
| C328 | Cancer | Cancer | Neopl mal laryngis overgribende flere regioner |
| C329 | Cancer | Cancer | Neopl mal laryngis uden specifikation |
| C33 | Cancer | Cancer | Kræft i luftrør |
| C339 | Cancer | Cancer | Neopl mal tracheae |
| C34 | Cancer | Cancer | Kræft i bronkie og lunge |
| C340 | Cancer | Cancer | Neopl mal bronchi hovedbronchus |
| C340A | Cancer | Cancer | Neopl mal pulmonis, hilus |
| C340B | Cancer | Cancer | Neopl mal tracheae, carina |
| C341 | Cancer | Cancer | Neopl mal pulmonis lobi superioris |
| C342 | Cancer | Cancer | Neopl mal pulmonis lobi medialis |
| C343 | Cancer | Cancer | Neopl mal pulmonis lobi inferioris |
| C348 | Cancer | Cancer | Neopl mal pulmonis overgribende flere regioner |
| C349 | Cancer | Cancer | Neopl mal bronchi sive pulmonis uden specifikation |
| C37 | Cancer | Cancer | Kræft i brissel |
| C379 | Cancer | Cancer | Neopl mal thymi |
| C38 | Cancer | Cancer | Kræft i hjerte, brysthule og lungehinde |
| C380 | Cancer | Cancer | Neopl mal cordis |
| C380A | Cancer | Cancer | Neopl mal pericardii |
| C381 | Cancer | Cancer | Neopl mal mediastini forreste del |
| C382 | Cancer | Cancer | Neopl mal mediastini bageste del |
| C383 | Cancer | Cancer | Neopl mal mediastini uden specifikation |
| C384 | Cancer | Cancer | Neopl mal pleurae |
| C388 | Cancer | Cancer | Neopl mal overgribende hjerte, mediastinum og pleura |
| C39 | Cancer | Cancer | Kræft m anden lokalis i åndedrætsorg og i org i brysthule |
| C390 | Cancer | Cancer | Neopl mal tractus respiratorii superioris uden specifikatio |
| C398 | Cancer | Cancer | Neopl mal org resp et intrathoracalium overgrib flere org |
| C399 | Cancer | Cancer | Neopl mal systematis resp m dårligt defineret lokalisation |
| C40 | Cancer | Cancer | Kræft i knogle og ledbrusk i ekstremiteter |
| C400 | Cancer | Cancer | Neopl mal scapulae, oss long et cart articuli extr superior |
| C400A | Cancer | Cancer | Neopl mal cartilaginis articuli ossium longorum extremitati |
| C400B | Cancer | Cancer | Neopl mal cartilaginis articuli scapulae |
| C400C | Cancer | Cancer | Neopl mal ossium longorum extremitatis superioris |
| C400D | Cancer | Cancer | Neopl mal scapulae |
| C401 | Cancer | Cancer | Neopl mal ossium brevium et cartilaginis articuli extr sup |
| C401A | Cancer | Cancer | Neopl mal cartilaginis articuli ossium brevium extrem. sup. |
| C401B | Cancer | Cancer | Neopl mal ossium brevium extremitatis superioris |
| C402 | Cancer | Cancer | Neopl mal ossium longorum et cartilaginis articuli extr inf |
| C402A | Cancer | Cancer | Neopl mal cartilaginis articuli ossium longorum extr inf |
| C402B | Cancer | Cancer | Neopl mal ossium longorum extremitatis inferioris |
| C403 | Cancer | Cancer | Neopl mal ossium brevium et cartilaginis articuli extr inf |
| C403A | Cancer | Cancer | Neopl mal cartilaginis articuli ossium brevium extrem. inf. |
| C403B | Cancer | Cancer | Neopl mal ossium brevium extremitatis inferioris |
| C408 | Cancer | Cancer | Neopl mal oss et cart articuli extr overgribende flere reg |
| C408A | Cancer | Cancer | Neopl mal cartilaginis articuli extremitatum, flere regione |
| C408B | Cancer | Cancer | Neopl mal ossium extremitatum, flere regioner |
| C409 | Cancer | Cancer | Neopl mal ossium et cartilaginis extremitatum uden specifik |
| C409A | Cancer | Cancer | Neopl mal cartilaginis articuli extremitatum uden specifik |
| C409B | Cancer | Cancer | Neopl mal ossium extremitatum uden specifikation |
| C41 | Cancer | Cancer | Kræft i knogle og ledbrusk m anden lokalisation eller u spe |
| C410 | Cancer | Cancer | Neopl mal ossium cranii et faciei |
| C410A | Cancer | Cancer | Neopl mal ossis cranii |
| C410B | Cancer | Cancer | Neopl mal ossis faciei |
| C410C | Cancer | Cancer | Neopl mal cartilaginis articuli cranii |
| C410D | Cancer | Cancer | Neopl mal cartilaginis articuli faciei |
| C410E | Cancer | Cancer | Knoglekræft i overkæben |
| C410F | Cancer | Cancer | Knoglekræft i øjenhule |
| C410G | Cancer | Cancer | Kræft i ledbrusk i overkæben |
| C411 | Cancer | Cancer | Neopl mal mandibulae |
| C411A | Cancer | Cancer | Neopl mal cartilaginis articuli mandibulae |
| C412 | Cancer | Cancer | Neopl mal columnae vertebralis |
| C412A | Cancer | Cancer | Neopl mal cartilaginis articuli columnae vertebralis |
| C412B | Cancer | Cancer | Knoglekræft i ryghvirvel |
| C413 | Cancer | Cancer | Neopl mal costae, sterni et claviculae |
| C413A | Cancer | Cancer | Neopl mal cartilaginis articuli claviculae |
| C413B | Cancer | Cancer | Neopl mal cartilaginis articuli costae |
| C413C | Cancer | Cancer | Neopl mal cartilaginis articuli sterni |
| C413D | Cancer | Cancer | Neopl mal claviculae |
| C413E | Cancer | Cancer | Neopl mal costae |
| C413F | Cancer | Cancer | Neopl mal sterni |
| C414 | Cancer | Cancer | Neopl mal ossis pelvis, sacri et coccygis |
| C414A | Cancer | Cancer | Neopl mal cartilaginis articuli ossis coccygis |
| C414B | Cancer | Cancer | Neopl mal cartilaginis articuli ossis pelvis |
| C414C | Cancer | Cancer | Neopl mal cartilaginis articuli ossis sacri |
| C414D | Cancer | Cancer | Neopl mal ossis coccygis |
| C414E | Cancer | Cancer | Neopl mal ossis pelvis |
| C414F | Cancer | Cancer | Neopl mal ossis sacri |
| C418 | Cancer | Cancer | Neopl mal oss et cart articuli overgribende flere regioner |
| C418A | Cancer | Cancer | Neopl mal cartilag articuli, flere reg (ikke ekstremiteter) |
| C418B | Cancer | Cancer | Neopl mal ossis, flere regioner (ikke extremiteter) |
| C419 | Cancer | Cancer | Neopl mal ossis et cartilaginis articuli uden specifikation |
| C419A | Cancer | Cancer | Neopl mal cartilaginis articuli uden spec (ikke ekstremitet |
| C419B | Cancer | Cancer | Neopl mal ossis uden specifikation (ikke ekstremiteter) |
| C43 | Cancer | Cancer | Ondartet melanom i hud |
| C430 | Cancer | Cancer | Melanoma mal labii oris |
| C430E | Cancer | Cancer | Malignt melanom i læbe, lentigo maligna |
| C430F | Cancer | Cancer | Malignt melanom i læbe, superficielt spredende |
| C430G | Cancer | Cancer | Malignt melanom i læbe, nodulært |
| C430H | Cancer | Cancer | Malignt melanom i læbe, akralt lentiginøst |
| C430J | Cancer | Cancer | Malignt melanom i læbe, malignt blue naevus |
| C430K | Cancer | Cancer | Malignt melanom i læbe, desmoplastisk |
| C430Z | Cancer | Cancer | Malignt melanom i læbe, anden type |
| C431 | Cancer | Cancer | Melanoma mal cutis palpebrae incl. canthus oculi |
| C431A | Cancer | Cancer | Melanoma mal cutis canthi oculi |
| C431B | Cancer | Cancer | Melanoma mal cutis palpebrae |
| C431E | Cancer | Cancer | Malignt melanom i øjenlåg, lentigo maligna |
| C431F | Cancer | Cancer | Malignt melanom i øjenlåg, superficielt spredende |
| C431G | Cancer | Cancer | Malignt melanom i øjenlåg, nodulært |
| C431H | Cancer | Cancer | Malignt melanom i øjenlåg, akralt lentiginøst |
| C431J | Cancer | Cancer | Malignt melanom i øjenlåg, malignt blue naevus |
| C431K | Cancer | Cancer | Malignt melanom i øjenlåg, desmoplastisk |
| C431Z | Cancer | Cancer | Malignt melanom i øjenlåg, anden type |
| C432 | Cancer | Cancer | Melanoma mal auris et meatus acustici ext |
| C432A | Cancer | Cancer | Melanoma mal auris |
| C432B | Cancer | Cancer | Melanoma mal canalis auris externae |
| C432C | Cancer | Cancer | Melanoma mal meatus acustici externi |
| C432E | Cancer | Cancer | Malignt melanom i hud i ydre øre, lentigo maligna |
| C432F | Cancer | Cancer | Malignt melanom i hud i ydre øre, superficielt spredende |
| C432G | Cancer | Cancer | Malignt melanom i hud i ydre øre, nodulært |
| C432H | Cancer | Cancer | Malignt melanom i hud i ydre øre, akralt lentiginøst |
| C432J | Cancer | Cancer | Malignt melanom i hud i ydre øre, malignt blue naevus |
| C432K | Cancer | Cancer | Malignt melanom i hud i ydre øre, desmoplstisk |
| C432Z | Cancer | Cancer | Malignt melanom i hud i ydre øre, anden type |
| C433 | Cancer | Cancer | Melanoma mal faciei, anden ell ikke spec. lokalisation |
| C433E | Cancer | Cancer | Malignt melanom i hud i ansigt med anden eller uspecificeret lokalisation, lentigo maligna |
| C433F | Cancer | Cancer | Malignt melanom i hud i ansigt med anden eller uspecificeret lokalisation, superficielt spredende |
| C433G | Cancer | Cancer | Malignt melanom i hud i ansigt med anden eller uspecificeret lokalisation, nodulært |
| C433H | Cancer | Cancer | Malignt melanom i hud i ansigt med anden eller uspecificeret lokalisation, akralt lentiginøst |
| C433J | Cancer | Cancer | Malignt melanom i hud i ansigt med anden eller uspecificeret lokalisation, malignt blue naevus |
| C433K | Cancer | Cancer | Malignt melanom i hud i ansigt med anden eller uspecificeret lokalisation, desmoplastisk |
| C433Z | Cancer | Cancer | Malignt melanom i hud i ansigt med anden eller uspecificeret lokalisation, anden type |
| C434 | Cancer | Cancer | Melanoma mal capillitii et colli |
| C434A | Cancer | Cancer | Melanoma mal capillitii |
| C434B | Cancer | Cancer | Melanoma mal colli |
| C434E | Cancer | Cancer | Malignt melanom i hud på skalp og hals, lentigo maligna |
| C434F | Cancer | Cancer | Malignt melanom i hud på skalp og hals, superficicielt spredende |
| C434G | Cancer | Cancer | Malignt melanom i hud på skalp og hals, nodulært |
| C434H | Cancer | Cancer | Malignt melanom i hud på skalp og hals, akralt lentiginøst |
| C434J | Cancer | Cancer | Malignt melanom i hud på skalp og hals, malignt blue naevus |
| C434K | Cancer | Cancer | Malignt melanom i hud på skalp og hals, desmoplastisk |
| C434Z | Cancer | Cancer | Malignt melanom i hud på skalp og hals, anden type |
| C435 | Cancer | Cancer | Melanoma mal trunci |
| C435A | Cancer | Cancer | Melanoma mal cutis ani |
| C435B | Cancer | Cancer | Melanoma mal cutis mammae |
| C435C | Cancer | Cancer | Melanoma mal cutis perianalis |
| C435D | Cancer | Cancer | Melanoma mal marginalis analis |
| C435E | Cancer | Cancer | Malignt melanom i hud på truncus, lentigo maligna |
| C435F | Cancer | Cancer | Malignt melanom i hud på truncus, superficielt spredende |
| C435G | Cancer | Cancer | Malignt melanom i hud på truncus, nodulært |
| C435H | Cancer | Cancer | Malignt melanom i hud på truncus, akralt lentiginøst |
| C435J | Cancer | Cancer | Malignt melanom i hud på truncus, malignt blue naevus |
| C435K | Cancer | Cancer | Malignt melanom i hud på truncus, desmoplastisk |
| C435L | Cancer | Cancer | Malignt melanom i anale marginalzone |
| C435M | Cancer | Cancer | Perianalt kutant malignt melanom |
| C435Z | Cancer | Cancer | Malignt melanom i hud på truncus, andet |
| C436 | Cancer | Cancer | Melanoma mal extr superioris incl. cingulum |
| C436A | Cancer | Cancer | Melanoma mal cinguli |
| C436B | Cancer | Cancer | Melanoma mal extremitatis superioris |
| C436C | Cancer | Cancer | Melanoma malignum digiti manus |
| C436E | Cancer | Cancer | Malignt melanom i hud på overekstremitet, lentigo maligna |
| C436F | Cancer | Cancer | Malignt melanom i hud på overekstremitet, superficielt spredende |
| C436G | Cancer | Cancer | Malignt melanom i hud på overekstremitet, nodulært |
| C436H | Cancer | Cancer | Malignt melanom i hud på overekstremitet, akralt lentiginøst |
| C436J | Cancer | Cancer | Malignt melanom i hud på overekstremitet, malignt blue naevus |
| C436K | Cancer | Cancer | Malignt melanom i hud på overekstremitet, desmoplastisk |
| C436Z | Cancer | Cancer | Malignt melanom i hud på overekstremitet, andet |
| C437 | Cancer | Cancer | Melanoma mal extr inferioris incl. regio coxae |
| C437A | Cancer | Cancer | Melanoma mal extremitatis inferioris |
| C437B | Cancer | Cancer | Melanoma mal regionis coxae |
| C437C | Cancer | Cancer | Melanoma malignum digiti pedis |
| C437E | Cancer | Cancer | Malignt melanom i hud på underekstremitet, lentigo maligna |
| C437F | Cancer | Cancer | Malignt melanom i hud på underekstremitet, superficielt spredende |
| C437G | Cancer | Cancer | Malignt melanom i hud på underekstremitet, nodulært |
| C437H | Cancer | Cancer | Malignt melanom i hud på underekstremitet, akralt lentiginøst |
| C437J | Cancer | Cancer | Malignt melanom i hud på underekstremitet, malignt blue naevus |
| C437K | Cancer | Cancer | Malignt melanom i hud på underekstremitet, desmoplastisk |
| C437Z | Cancer | Cancer | Malignt melanom i hud på underekstremitet, anden type |
| C438 | Cancer | Cancer | Melanoma mal overgribende flere regioner |
| C438E | Cancer | Cancer | Malignt melanom i hud overgribende flere regioner, lentigo maligna |
| C438F | Cancer | Cancer | Malignt melanom i hud overgribende flere regioner, superficielt spredende |
| C438G | Cancer | Cancer | Malignt melanom i hud overgribende flere regioner, nodulært |
| C438H | Cancer | Cancer | Malignt melanom i hud overgribende flere regioner, akralt lentiginøst |
| C438J | Cancer | Cancer | Malignt melanom i hud overgribende flere regioner, malignt blue naevus |
| C438K | Cancer | Cancer | Malignt melanom i hud overgribende flere regioner, desmoplastisk |
| C438Z | Cancer | Cancer | Malignt melanom i hud overgribende flere regioner, anden type |
| C439 | Cancer | Cancer | Melanoma mal uden specifikation |
| C439E | Cancer | Cancer | Malignt melanom i hud uden specifikation, lentigo maligna |
| C439F | Cancer | Cancer | Malignt melanom i hud uden specifikation, superficielt spredende |
| C439G | Cancer | Cancer | Malignt melanom i hud uden specifikation, nodulært |
| C439H | Cancer | Cancer | Malignt melanom i hud uden specifikation, akralt lentiginøst |
| C439J | Cancer | Cancer | Malignt melanom i hud uden specifikation, malignt blue naevus |
| C439K | Cancer | Cancer | Malignt melanom i hud uden specifikation, desmoplastisk |
| C439Z | Cancer | Cancer | Malignt melanom i hud uden specifikation, anden type |
| C45 | Cancer | Cancer | Mesoteliom |
| C450 | Cancer | Cancer | Mesothelioma pleurae |
| C451 | Cancer | Cancer | Mesothelioma peritonei |
| C452 | Cancer | Cancer | Mesothelioma pericardii |
| C457 | Cancer | Cancer | Mesothelioma anden lokalisation |
| C459 | Cancer | Cancer | Mesothelioma uden specifikation |
| C46 | Cancer | Cancer | Kaposi's sarkom |
| C460 | Cancer | Cancer | Sarcoma Kaposi cutis |
| C461 | Cancer | Cancer | Sarcoma Kaposi telae conjunctivae |
| C462 | Cancer | Cancer | Sarcoma Kaposi palati |
| C463 | Cancer | Cancer | Sarcoma Kaposi lymphonodi |
| C467 | Cancer | Cancer | Sarcoma Kaposi andre lokalisationer |
| C468 | Cancer | Cancer | Sarcoma Kaposi multiple organer |
| C469 | Cancer | Cancer | Sarcoma Kaposi uden specifikation |
| C47 | Cancer | Cancer | Kræft i perifere nerver og autonome nervesystem |
| C470 | Cancer | Cancer | Neopl mal n periph et syst nervosi autonom cap faciei colli |
| C470A | Cancer | Cancer | Neopl mal nervi peripherici capitis |
| C470B | Cancer | Cancer | Neopl mal nervi peripherici colli |
| C470C | Cancer | Cancer | Neopl mal nervi peripherici faciei |
| C470D | Cancer | Cancer | Neopl mal systematis nervosi autonomici capitis |
| C470E | Cancer | Cancer | Neopl mal systematis nervosi autonomici colli |
| C470F | Cancer | Cancer | Neopl mal systematis nervosi autonomici faciei |
| C471 | Cancer | Cancer | Neopl mal n periph et syst nervosi autonom extr superioris |
| C471A | Cancer | Cancer | Neopl mal nervi peripherici cinguli |
| C471B | Cancer | Cancer | Neopl mal nervi peripherici extremitatis superioris |
| C471C | Cancer | Cancer | Neopl mal systematis nervosi autonomici cinguli |
| C471D | Cancer | Cancer | Neopl mal systematis nervosi autonomici extremitatis sup |
| C472 | Cancer | Cancer | Neopl mal n periph et syst nervosi autonom extr inferioris |
| C472A | Cancer | Cancer | Neopl mal nervi peripherici regionis coxae |
| C472B | Cancer | Cancer | Neopl mal nervi peripherici extremitatis inferioris |
| C472C | Cancer | Cancer | Neopl mal systematis nervosi autonomici regionis coxae |
| C472D | Cancer | Cancer | Neopl mal systematis nervosi autonomici extremitatis inf |
| C473 | Cancer | Cancer | Neopl mal n periph et syst nervosi autonom thoracis |
| C473A | Cancer | Cancer | Neopl mal nervi peripherici thoracis |
| C473B | Cancer | Cancer | Neopl mal systematis nervosi autonomici thoracis |
| C474 | Cancer | Cancer | Neopl mal n peripher et syst nervosi autonom abdominis |
| C474A | Cancer | Cancer | Neopl mal nervi peripherici abdominis |
| C474B | Cancer | Cancer | Neopl mal systematis nervosi autonomici abdominis |
| C475 | Cancer | Cancer | Neopl mal n peripher et syst nervosi autonom pelvis |
| C475A | Cancer | Cancer | Neopl mal nervi peripherici pelvis |
| C475B | Cancer | Cancer | Neopl mal systematis nervosi autonomici pelvis |
| C476 | Cancer | Cancer | Neopl mal n peripher et syst nervosi autonom trunci u spec |
| C476A | Cancer | Cancer | Neopl mal nervi peripherici trunci uden specifikation |
| C476B | Cancer | Cancer | Neopl mal systematis nervosi autonomici trunci uden spec |
| C478 | Cancer | Cancer | Neopl mal n peripher et syst nervosi overgribende flere reg |
| C478A | Cancer | Cancer | Neopl mal nervi peripherici, flere regioner |
| C478B | Cancer | Cancer | Neopl mal systematis nervosi autonomici, flere regioner |
| C479 | Cancer | Cancer | Neopl mal n peripher et syst nervosi autonom uden specifik |
| C479A | Cancer | Cancer | Neopl mal nervi peripherici uden specifikation |
| C479B | Cancer | Cancer | Neopl mal systematis nervosi autonomici uden specifikation |
| C48 | Cancer | Cancer | Kræft i bughinde og bindevæv i bughulens bagvæg |
| C480 | Cancer | Cancer | Neopl mal telae conjunctivae retroperitonei |
| C481 | Cancer | Cancer | Neopl mal telae conjunctivae peritonei |
| C481A | Cancer | Cancer | Neopl mal telae conjunctivae mesenterii |
| C481B | Cancer | Cancer | Neopl mal telae conjunctivae mesocoli |
| C481C | Cancer | Cancer | Neopl mal telae conjunctivae omenti |
| C482 | Cancer | Cancer | Neopl mal telae conjunctivae peritonei uden specifikation |
| C488 | Cancer | Cancer | Neopl mal telae conj overgrib bindevæv i og bag bughinde |
| C49 | Cancer | Cancer | Kræft i andet bindevæv |
| C490 | Cancer | Cancer | Neopl mal telae conjunctivae capitis, faciei et colli |
| C490A | Cancer | Cancer | Neopl mal telae conjunctivae capitis |
| C490B | Cancer | Cancer | Neopl mal telae conjunctivae colli |
| C490C | Cancer | Cancer | Neopl mal telae conjunctivae faciei |
| C491 | Cancer | Cancer | Neopl mal telae conjunctivae extr superioris incl. cingulum |
| C491A | Cancer | Cancer | Neopl mal telae conjunctivae cinguli |
| C491B | Cancer | Cancer | Neopl mal telae conjunctivae extremitatis superioris |
| C492 | Cancer | Cancer | Neopl mal telae conjunctivae extr inf incl. regio coxae |
| C492A | Cancer | Cancer | Neopl mal telae conjunctivae extremitatis inferioris |
| C492B | Cancer | Cancer | Neopl mal telae conjunctivae regionis coxae |
| C493 | Cancer | Cancer | Neopl mal telae conjunctivae thoracis |
| C493A | Cancer | Cancer | Neopl mal telae conjunctivae axillae |
| C493B | Cancer | Cancer | Neopl mal telae conjunctivae diaphragmatis |
| C493C | Cancer | Cancer | Neopl mal telae conjunctivae thoracis |
| C494 | Cancer | Cancer | Neopl mal telae conjunctivae abdominis |
| C495 | Cancer | Cancer | Neopl mal telae conjunctivae pelvis |
| C495A | Cancer | Cancer | Neopl mal telae conjunctivae perinei |
| C496 | Cancer | Cancer | Neopl mal telae conjunctivae trunci uden specifikation |
| C498 | Cancer | Cancer | Neopl mal telae conjunctivae overgribende flere regioner |
| C498A | Cancer | Cancer | Malignt fibrøst histiocytom |
| C499 | Cancer | Cancer | Neopl mal telae conjunctivae uden specifikation |
| C50 | Cancer | Cancer | Kræft i bryst |
| C500 | Cancer | Cancer | Neopl mal papillae et areolae mammae |
| C500A | Cancer | Cancer | Neopl mal mammae, areola |
| C500B | Cancer | Cancer | Neopl mal mammae, papilla |
| C500C | Cancer | Cancer | Paget's sygdom i mamma |
| C500D | Cancer | Cancer | Paget's disease of the nipple |
| C501 | Cancer | Cancer | Neopl mal mammae centrale del |
| C502 | Cancer | Cancer | Neopl mal mammae øvre mediale kvadrant |
| C503 | Cancer | Cancer | Neopl mal mammae nedre mediale kvadrant |
| C504 | Cancer | Cancer | Neopl mal mammae øvre laterale kvadrant |
| C505 | Cancer | Cancer | Neopl mal mammae nedre laterale kvadrant |
| C506 | Cancer | Cancer | Neopl mal mammae processus axillaris |
| C508 | Cancer | Cancer | Neopl mal mammae overgribende flere regioner |
| C509 | Cancer | Cancer | Neopl mal mammae uden specifikation |
| C51 | Cancer | Cancer | Kræft i ydre kvindelige kønsorganer |
| C510 | Cancer | Cancer | Neopl mal labii majoris vulvae |
| C510A | Cancer | Cancer | Neopl mal glandulae vestibularis Bartholini |
| C511 | Cancer | Cancer | Neopl mal labii minoris vulvae |
| C512 | Cancer | Cancer | Neopl mal clitoridis |
| C518 | Cancer | Cancer | Neopl mal vulvae overgribende flere regioner |
| C519 | Cancer | Cancer | Neopl mal vulvae uden specifikation |
| C519D | Cancer | Cancer | Neoplasma malignum vulvae uden specifikation, basocellulært, nodulært |
| C519E | Cancer | Cancer | Neoplasma malignum vulvae uden specifikation, basocellulært, superficielt |
| C519F | Cancer | Cancer | Neoplasma malignum vulvae uden specifikation, basocellulært, morphaeatype |
| C519G | Cancer | Cancer | Neoplasma malignum vulvae uden specifikation, basocellulært, multiplex |
| C519H | Cancer | Cancer | Neoplasma malignum vulvae uden specifikation, basocellulært, recidivans |
| C519J | Cancer | Cancer | Neoplasma malignum vulvae uden specifikation, planocellulært, verrukøst (cuniculatum, Buschke-Löwenstein) |
| C519K | Cancer | Cancer | Neoplasma malignum vulvae uden specifikation, planocellulært, spinocellulært |
| C519L | Cancer | Cancer | Neoplasma malignum vulvae uden specifikation, planocellulært, Marjorlin/Melaney |
| C519M | Cancer | Cancer | Neoplasma malignum vulvae uden specifikation, planocellulært, recidivans |
| C519N | Cancer | Cancer | Neoplasma malignum vulvae uden specifikation, mikrocystisk adneks karcinom |
| C519P | Cancer | Cancer | Neoplasma malignum vulvae uden specifikation, Merkelcelle karcinom |
| C519Q | Cancer | Cancer | Neoplasma malignum vulvae uden specifikation, adenoidt cystisk karcinom |
| C519R | Cancer | Cancer | Neoplasma malignum vulvae uden specifikation, mb. Paget (ekstramammært) |
| C519S | Cancer | Cancer | Neoplasma malignum vulvae uden specifikation, porokarcinom |
| C519T | Cancer | Cancer | Neoplasma malignum vulvae uden specifikation, sebaceøst karcinom |
| C519U | Cancer | Cancer | Neoplasma malignum vulvae uden specifikation, trichillemmalt karcinom |
| C519V | Cancer | Cancer | Neoplasma malignum vulvae uden specifikation, dermatofibrosarcoma protuberans |
| C519W | Cancer | Cancer | Neoplasma malignum vulvae uden specifikation, angiosarkom |
| C519Z | Cancer | Cancer | Neoplasma malignum vulvae uden specifikation, andet |
| C52 | Cancer | Cancer | Kræft i skede |
| C529 | Cancer | Cancer | Neopl mal vaginae |
| C53 | Cancer | Cancer | Kræft i livmoderhals |
| C530 | Cancer | Cancer | Neopl mal endocervicis uteri |
| C531 | Cancer | Cancer | Neopl mal cervicis uteri, planocellulært carcinom st. I |
| C532 | Cancer | Cancer | Neopl mal cervicis uteri, planocellulært carcinom st. II |
| C533 | Cancer | Cancer | Neopl mal cervicis uteri, planocellulært carcinom st. III |
| C534 | Cancer | Cancer | Neopl mal cervicis uteri, planocellulært carcinom st. IV |
| C538 | Cancer | Cancer | Neopl mal cervicis uteri overgribende flere regioner |
| C539 | Cancer | Cancer | Neopl mal cervicis uteri uden specifikation |
| C54 | Cancer | Cancer | Kræft i livmoder |
| C540 | Cancer | Cancer | Neopl mal isthmi uteri, st. I |
| C541 | Cancer | Cancer | Neopl mal fundi uteri, st. I |
| C542 | Cancer | Cancer | Neopl mal myometrii, alle stadier |
| C543 | Cancer | Cancer | Neopl mal endometrii, st. I |
| C544 | Cancer | Cancer | Neopl mal endometrii, st. II |
| C545 | Cancer | Cancer | Neopl mal endometrii, st. III |
| C546 | Cancer | Cancer | Neopl mal endometrii, st. IV |
| C548 | Cancer | Cancer | Neopl mal corporis uteri overgribende flere regioner |
| C549 | Cancer | Cancer | Neopl mal corporis uteri uden specifikation |
| C55 | Cancer | Cancer | Kræft i livmoder uden specifikation |
| C559 | Cancer | Cancer | Neopl mal uteri uden specifikation |
| C56 | Cancer | Cancer | Kræft i æggestok |
| C560 | Cancer | Cancer | Neopl mal ovarii, st. I |
| C561 | Cancer | Cancer | Neopl mal ovarii, st. II |
| C562 | Cancer | Cancer | Neopl mal ovarii, st. III |
| C563 | Cancer | Cancer | Neopl mal ovarii, st. IV |
| C569 | Cancer | Cancer | Neopl mal ovarii uden specifikation |
| C569A | Cancer | Cancer | Ekstragonadal germinalcelletumor (ektopisk ovarie) |
| C57 | Cancer | Cancer | Kræft i andre og ikke specificerede kvindelige kønsorganer |
| C570 | Cancer | Cancer | Neopl mal tubae uterinae |
| C571 | Cancer | Cancer | Neopl mal ligamenti lati uteri |
| C572 | Cancer | Cancer | Neopl mal ligamenti teres uteri |
| C573 | Cancer | Cancer | Neopl mal parametrii |
| C574 | Cancer | Cancer | Neopl mal adnexae uteri uden specifikation |
| C577 | Cancer | Cancer | Neopl mal genitalium feminae spec på anden måde |
| C578 | Cancer | Cancer | Neopl mal genitalium feminae overgribende flere regioner |
| C579 | Cancer | Cancer | Neopl mal genitalium feminae uden specifikation |
| C58 | Cancer | Cancer | Kræft i moderkage |
| C589 | Cancer | Cancer | Neopl mal placentae |
| C589A | Cancer | Cancer | Choriokarcinom i placenta |
| C589B | Cancer | Cancer | Chorionepiteliom i placenta |
| C60 | Cancer | Cancer | Kræft i penis |
| C600 | Cancer | Cancer | Neopl mal praeputii |
| C601 | Cancer | Cancer | Neopl mal glandis penis |
| C602 | Cancer | Cancer | Neopl mal corporis penis |
| C608 | Cancer | Cancer | Neopl mal penis overgribende flere regioner |
| C609 | Cancer | Cancer | Neopl mal penis uden specifikation |
| C609D | Cancer | Cancer | Neoplasma malignum penis uden specifikation, basocellulært, nodulært |
| C609E | Cancer | Cancer | Neoplasma malignum penis uden specifikation, basocellulært, superficielt |
| C609F | Cancer | Cancer | Neoplasma malignum penis uden specifikation, basocellulært, morphaeatype |
| C609G | Cancer | Cancer | Neoplasma malignum penis uden specifikation, basocellulært, multiplex |
| C609H | Cancer | Cancer | Neoplasma malignum penis uden specifikation, basocellulært, recidivans |
| C609J | Cancer | Cancer | Neoplasma malignum penis uden specifikation, planocellulært, verrukøst (cuniculatum, Buschke-Löwenstein) |
| C609K | Cancer | Cancer | Neoplasma malignum penis uden specifikation, planocellulært, spinocellulært |
| C609L | Cancer | Cancer | Neoplasma malignum penis uden specifikation, planocellulært, Marjorlin/Melaney |
| C609M | Cancer | Cancer | Neoplasma malignum penis uden specifikation, planocellulært, recidivans |
| C609N | Cancer | Cancer | Neoplasma malignum penis uden specifikation, mikrocystisk adneks karcinom |
| C609P | Cancer | Cancer | Neoplasma malignum penis uden specifikation, Merkelcelle karcinom |
| C609Q | Cancer | Cancer | Neoplasma malignum penis uden specifikation, adenoidt cystisk karcinom |
| C609R | Cancer | Cancer | Neoplasma malignum penis uden specifikation, mb. Paget (ekstramammært) |
| C609S | Cancer | Cancer | Neoplasma malignum penis uden specifikation, porokarcinom |
| C609T | Cancer | Cancer | Neoplasma malignum penis uden specifikation, sebaceøst karcinom |
| C609U | Cancer | Cancer | Neoplasma malignum penis uden specifikation, trichillemmalt karcinom |
| C609V | Cancer | Cancer | Neoplasma malignum penis uden specifikation, dermatofibrosarcoma protuberans |
| C609W | Cancer | Cancer | Neoplasma malignum penis uden specifikation, angiosarkom |
| C609Z | Cancer | Cancer | Neoplasma malignum penis uden specifikation, andet |
| C61 | Cancer | Cancer | Kræft i blærehalskirtel |
| C619 | Cancer | Cancer | Neopl mal prostatae |
| C62 | Cancer | Cancer | Kræft i testikel |
| C620 | Cancer | Cancer | Neopl mal testis i forbind m retention og ektopi af testis |
| C621 | Cancer | Cancer | Neopl mal testis scroti |
| C629 | Cancer | Cancer | Neopl mal testis uden specifikation |
| C629A | Cancer | Cancer | Ekstragonadal germinalcelletumor (testis) |
| C63 | Cancer | Cancer | Kræft i andre og ikke specificerede mandlige kønsorganer |
| C630 | Cancer | Cancer | Neopl mal epididymidis |
| C631 | Cancer | Cancer | Neopl mal funiculi spermatici |
| C632 | Cancer | Cancer | Neopl mal scroti |
| C637 | Cancer | Cancer | Neopl mal genitalium viri aliorum |
| C637A | Cancer | Cancer | Neopl mal tunicae vaginalis scroti |
| C637B | Cancer | Cancer | Neopl mal vesiculae seminalis |
| C638 | Cancer | Cancer | Neopl mal genitalium viri overgribende flere regioner |
| C639 | Cancer | Cancer | Neopl mal genitalium viri uden specifikation |
| C64 | Cancer | Cancer | Kræft i nyre undtagen nyrebækken |
| C649 | Cancer | Cancer | Neopl mal renis excl. pelvis renis |
| C65 | Cancer | Cancer | Kræft i nyrebækken |
| C659 | Cancer | Cancer | Neopl mal pelvis renis |
| C66 | Cancer | Cancer | Kræft i urinleder |
| C669 | Cancer | Cancer | Neopl mal ureteris |
| C67 | Cancer | Cancer | Kræft i urinblære |
| C670 | Cancer | Cancer | Neopl mal trigoni vesicae urinariae |
| C671 | Cancer | Cancer | Neopl mal vesicae urinariae loft |
| C672 | Cancer | Cancer | Neopl mal vesicae urinariae sidevæg |
| C673 | Cancer | Cancer | Neopl mal vesicae urinariae forvæg |
| C674 | Cancer | Cancer | Neopl mal vesicae urinariae bagvæg |
| C675 | Cancer | Cancer | Neopl mal cervicis vesicae urinariae |
| C675A | Cancer | Cancer | Neopl mal vesicae urinariae, orificium internum urethrae |
| C676 | Cancer | Cancer | Neopl mal orificii ureteris |
| C677 | Cancer | Cancer | Neopl mal urachi |
| C678 | Cancer | Cancer | Neopl mal vesicae urinariae overgribende flere regioner |
| C679 | Cancer | Cancer | Neopl mal vesicae urinariae uden specifikation |
| C68 | Cancer | Cancer | Kræft i andre og ikke specificerede urinorganer |
| C680 | Cancer | Cancer | Neopl mal urethrae |
| C681 | Cancer | Cancer | Neopl mal glandulae paraurethralis |
| C688 | Cancer | Cancer | Neopl mal organorum urinariorum overgribende flere reg |
| C689 | Cancer | Cancer | Neopl mal organi urinarii uden specifikation |
| C69 | Cancer | Cancer | Kræft i øje og øjenomgivelser |
| C690 | Cancer | Cancer | Neopl mal conjunctivae |
| C691 | Cancer | Cancer | Neopl mal corneae |
| C692 | Cancer | Cancer | Neopl mal retinae |
| C692A | Cancer | Cancer | Retinoblastom |
| C693 | Cancer | Cancer | Neopl mal chorioideae |
| C694 | Cancer | Cancer | Neopl mal corporis ciliaris oculi |
| C694A | Cancer | Cancer | Neopl mal oculi, bulbus |
| C695 | Cancer | Cancer | Neopl mal glandulae et ductus lacrimalis |
| C695A | Cancer | Cancer | Neopl mal ductus lacrimalis |
| C695B | Cancer | Cancer | Neopl mal glandulae lacrimalis |
| C696 | Cancer | Cancer | Neopl mal orbitae |
| C697 | Cancer | Cancer | Malignt melanom i øjet |
| C698 | Cancer | Cancer | Neopl mal oculi et adnexae overgribende flere regioner |
| C699 | Cancer | Cancer | Neopl mal oculi uden specifikation |
| C699A | Cancer | Cancer | Kræft i øjeæble |
| C70 | Cancer | Cancer | Kræft i hjernehinder og rygmarvshinder |
| C700 | Cancer | Cancer | Neopl mal meningum cerebri |
| C701 | Cancer | Cancer | Neopl mal meningum medullae spinalis |
| C709 | Cancer | Cancer | Neopl mal meningum uden specifikation |
| C71 | Cancer | Cancer | Kræft i hjerne |
| C710 | Cancer | Cancer | Neopl mal cerebri excl. lobi et ventriculi |
| C710A | Cancer | Cancer | Neopl mal cerebri, corpus callosum |
| C710B | Cancer | Cancer | Neopl mal cerebri supratentoriale uden specifikation |
| C711 | Cancer | Cancer | Neopl mal cerebri lobus frontalis |
| C712 | Cancer | Cancer | Neopl mal cerebri lobus temporalis |
| C713 | Cancer | Cancer | Neopl mal cerebri lobus parietalis |
| C714 | Cancer | Cancer | Neopl mal cerebri lobus occipitalis |
| C715 | Cancer | Cancer | Neopl mal cerebri intraventriculare |
| C716 | Cancer | Cancer | Neopl mal cerebelli |
| C717 | Cancer | Cancer | Neopl mal cerebri hjernestamme og 4. ventrikel |
| C717A | Cancer | Cancer | Neopl mal cerebri, 4 ventrikel |
| C717B | Cancer | Cancer | Neopl mal cerebri, hjernestamme |
| C717C | Cancer | Cancer | Neopl mal cerebri infratentoriale uden specifikation |
| C718 | Cancer | Cancer | Neopl mal cerebri overgribende flere regioner |
| C719 | Cancer | Cancer | Neopl mal cerebri uden specifikation |
| C72 | Cancer | Cancer | Kræft i rygmarv, kranienerver og andre dele af nervesyst |
| C720 | Cancer | Cancer | Neopl mal medullae spinalis |
| C721 | Cancer | Cancer | Neopl mal caudae equinae |
| C722 | Cancer | Cancer | Neopl mal nervi olfactorii |
| C722A | Cancer | Cancer | Neopl mal bulbi olfactorii |
| C723 | Cancer | Cancer | Neopl mal nervi optici |
| C724 | Cancer | Cancer | Neopl mal nervi acustici |
| C725 | Cancer | Cancer | Neopl mal nervi cranialis m anden lokalisation eller u spec |
| C728 | Cancer | Cancer | Neopl mal overgribende hjerne og centralnervesystem |
| C728A | Cancer | Cancer | Carcinomatosis pleurae |
| C729 | Cancer | Cancer | Neopl mal systematis nervosi centralis uden specifikation |
| C73 | Cancer | Cancer | Kræft i skjoldbruskkirtel |
| C739 | Cancer | Cancer | Neopl mal glandulae thyreoideae |
| C74 | Cancer | Cancer | Kræft i binyre |
| C740 | Cancer | Cancer | Neopl mal glandulae suprarenalis cortex |
| C741 | Cancer | Cancer | Neopl mal glandulae suprarenalis medulla |
| C749 | Cancer | Cancer | Neopl mal glandulae suprarenalis uden specifikation |
| C75 | Cancer | Cancer | Kræft i andre endokrine kirtler og lignende strukturer |
| C750 | Cancer | Cancer | Neopl mal glandulae parathyreoideae |
| C751 | Cancer | Cancer | Neopl mal glandulae pituitariae |
| C752 | Cancer | Cancer | Neopl mal ductus craniopharyngei |
| C753 | Cancer | Cancer | Neopl mal corporis pinealis |
| C754 | Cancer | Cancer | Neopl mal glomeris carotici |
| C755 | Cancer | Cancer | Neopl mal corporis paraaortici et paragangliosae |
| C755A | Cancer | Cancer | Neopl mal corporis paraaortici |
| C755B | Cancer | Cancer | Neopl mal paragangliosum |
| C758 | Cancer | Cancer | Neopl mal pluriglandulare uden specifikation |
| C759 | Cancer | Cancer | Neopl mal glandulae endocrinae uden specifikation |
| C76 | Cancer | Cancer | Kræft m dårligt definerede, sek eller ikke spec lokalisat |
| C760 | Cancer | Cancer | Neopl mal capitis, faciei, colli uden specifikation |
| C760A | Cancer | Cancer | Neopl mal capitis uden specifikation |
| C760B | Cancer | Cancer | Neopl mal colli uden specifikation |
| C760C | Cancer | Cancer | Neopl mal faciei uden specifikation |
| C761 | Cancer | Cancer | Neopl mal thoracis uden specifikation |
| C762 | Cancer | Cancer | Neopl mal abdominis uden specifikation |
| C763 | Cancer | Cancer | Neopl mal pelvis uden specifikation |
| C764 | Cancer | Cancer | Neopl mal extr superioris uden specifikation |
| C765 | Cancer | Cancer | Neopl mal extr inferioris uden specifikation |
| C767 | Cancer | Cancer | Neopl mal anden specificeret lokalisation |
| C768 | Cancer | Cancer | Neopl mal overgribende flere dårligt definerede lokalisat |
| C77 | Cancer | Cancer | Metastatisk eller ikke spec kræft i lymfeknude |
| C770 | Cancer | Cancer | Neopl mal lymph cap faciei colli supraclav metast ell u spe |
| C770A | Cancer | Cancer | Neopl mal lymphonodi capitis, metastatisk |
| C770B | Cancer | Cancer | Neopl mal lymphonodi capitis uden specifikation |
| C770C | Cancer | Cancer | Neopl mal lymphonodi colli, metastatisk |
| C770D | Cancer | Cancer | Neopl mal lymphonodi colli uden specifikation |
| C770E | Cancer | Cancer | Neopl mal lymphonodi faciei, metastatisk |
| C770F | Cancer | Cancer | Neopl mal lymphonodi faciei uden specifikation |
| C770G | Cancer | Cancer | Neopl mal lymphonodi supraclavicularis, metastatisk |
| C770H | Cancer | Cancer | Neopl mal lymphonodi supraclavicularis uden specifikation |
| C771 | Cancer | Cancer | Neopl mal lymph intrathoracalis metast ell uden specifik |
| C771A | Cancer | Cancer | Neopl mal lymphonodi intrathoracalis, metastatisk |
| C771B | Cancer | Cancer | Neopl mal lymphonodi intrathoracalis uden specifikation |
| C772 | Cancer | Cancer | Neopl mal lymphonodi intraabdominalis metast ell uden spec |
| C772A | Cancer | Cancer | Neopl mal lymphonodi intraabdominalis, metastatisk |
| C772B | Cancer | Cancer | Neopl mal lymphonodi intraabdominalis uden specifikation |
| C773 | Cancer | Cancer | Neopl mal lymphonodi axillae et extr sup metast ell u spec |
| C773A | Cancer | Cancer | Neopl mal lymphonodi axillae, metastatisk |
| C773B | Cancer | Cancer | Neopl mal lymphonodi axillae uden specifikation |
| C773C | Cancer | Cancer | Neopl mal lymphonodi extremitatis superioris, metastatisk |
| C773D | Cancer | Cancer | Neopl mal lymphonodi extremitatis superioris uden specifik |
| C773E | Cancer | Cancer | Metastase i pektoral lymfeknude |
| C773F | Cancer | Cancer | Kræft UNS i pektoral lymfeknude |
| C774 | Cancer | Cancer | Neopl mal lymph inguinalis et extr inf metast ell uden spec |
| C774A | Cancer | Cancer | Neopl mal lymphonodi extremitatis inferioris, metastatisk |
| C774B | Cancer | Cancer | Neopl mal lymphonodi extremitatis inferioris uden specifik |
| C774C | Cancer | Cancer | Neopl mal lymphonodi inguinalis, metastatisk |
| C774D | Cancer | Cancer | Neopl mal lymphonodi inguinalis uden specifikation |
| C775 | Cancer | Cancer | Neopl mal lymphonodi pelvis metastatisk eller ikke spec |
| C775A | Cancer | Cancer | Neopl mal lymphonodi pelvis, metastatisk |
| C775B | Cancer | Cancer | Neopl mal lymphonodi pelvis uden specifikation |
| C778 | Cancer | Cancer | Neopl mal lymphonodi multiplex metastatisk eller ikke spec |
| C778A | Cancer | Cancer | Neopl mal lymphonodi multiplex, metastatisk |
| C778B | Cancer | Cancer | Neopl mal lymphonodi multiplex uden specifikation |
| C779 | Cancer | Cancer | Neopl mal lymphonodi metastatisk eller ikke spec |
| C779A | Cancer | Cancer | Neopl mal lymphonodi, metastatisk |
| C779B | Cancer | Cancer | Neopl mal lymphonodi uden specifikation |
| C78 | Cancer | Cancer | Metastatisk kræft i åndedrætsorganer og fordøjelsessystem |
| C780 | Cancer | Cancer | Neopl mal pulmonis metastaticum |
| C781 | Cancer | Cancer | Neopl mal mediastini metastaticum |
| C782 | Cancer | Cancer | Neopl mal pleurae metastaticum |
| C782A | Cancer | Cancer | Carcinomatosis pleurae |
| C782B | Cancer | Cancer | Malign pleural effusion |
| C783 | Cancer | Cancer | Neopl mal organorum aliorum respirationis metastaticum |
| C784 | Cancer | Cancer | Neopl mal intestini tenuis metastaticum |
| C784A | Cancer | Cancer | Metastase i duodenum |
| C785 | Cancer | Cancer | Neopl mal coli et recti metastaticum |
| C785A | Cancer | Cancer | Neopl mal coli metastaticum |
| C785B | Cancer | Cancer | Neopl mal recti metastaticum |
| C786 | Cancer | Cancer | Neopl mal retroperitonei et peritonei metastaticum |
| C786B | Cancer | Cancer | Neopl mal peritonei metastaticum |
| C786C | Cancer | Cancer | Neopl mal retroperitoneale metastaticum |
| C786D | Cancer | Cancer | Carcinomatosis peritonei |
| C786E | Cancer | Cancer | Pseudomyxoma peritonei |
| C787 | Cancer | Cancer | Neopl mal hepatis metastaticum |
| C788 | Cancer | Cancer | Neopl mal metastaticum i andre eller u spec fordøjelsesorg |
| C79 | Cancer | Cancer | Metastatisk kræft i andre specificerede lokalisationer |
| C790 | Cancer | Cancer | Neopl mal renis et pelvis renis metastaticum |
| C790A | Cancer | Cancer | Neopl mal pelvis renis metastaticum |
| C790B | Cancer | Cancer | Neopl mal renis metastaticum |
| C791 | Cancer | Cancer | Neopl mal metast ves urinariae og i andre urinorg uden spec |
| C791I | Cancer | Cancer | Neoplasma malignum metastastic. vesicae urinariae |
| C791J | Cancer | Cancer | Neopl. malignum metastaticum prostatae |
| C791S | Cancer | Cancer | Neopl. malignum metastaticum penis |
| C791T | Cancer | Cancer | Neopl. malignum metastaticum testis |
| C791U | Cancer | Cancer | Neoplasma malignum metastaticum ureteris |
| C791V | Cancer | Cancer | Neopl. malignum metastaticum urethrae |
| C791X | Cancer | Cancer | Neopl.mal.metastastic., i urinorg., overgrib.fl.org/regioner |
| C793 | Cancer | Cancer | Neopl mal cerebri et meningum cerebri metastaticum |
| C793A | Cancer | Cancer | Neopl mal cerebri metastaticum |
| C793B | Cancer | Cancer | Neopl mal meningum cerebri metastaticum |
| C793C | Cancer | Cancer | Neopl mal meningum medullae spinalis metastaticum |
| C794 | Cancer | Cancer | Neopl mal metastaticum aliud systematis nervosi metast |
| C795 | Cancer | Cancer | Neopl mal ossis et medullae ossium metastaticum |
| C795A | Cancer | Cancer | Neopl mal medullae ossium metastaticum |
| C795B | Cancer | Cancer | Neopl mal ossis metastaticum |
| C795C | Cancer | Cancer | Metastase i ryghvirvel |
| C795D | Cancer | Cancer | Metastase i ribben |
| C795E | Cancer | Cancer | Metastase i kraniet |
| C796 | Cancer | Cancer | Neopl mal ovarii metastaticum |
| C797 | Cancer | Cancer | Neopl mal glandulae suprarenalis metastaticum |
| C798 | Cancer | Cancer | Neopl mal metastaticum andre specificerede lokalisationer |
| C798A | Cancer | Cancer | Carcinomatosis UNS |
| C80 | Cancer | Cancer | Kræft uden specifikation m hensyn til lokalisation |
| C809 | Cancer | Cancer | Neopl mal uden specifikation m hensyn til lokalisation |
| C81 | Cancer | Cancer | Malignt lymfom af Hodgkin type |
| C810 | Cancer | Cancer | Lymphoma Hodgkin 'lymphocytic predominance' |
| C811 | Cancer | Cancer | Lymphoma Hodgkin 'nodular sclerosis' |
| C812 | Cancer | Cancer | Lymphoma Hodgkin 'mixed cellularity' |
| C813 | Cancer | Cancer | Lymphoma Hodgkin 'lymphocytic depletion' |
| C814 | Cancer | Cancer | Klassisk lymfocytrigt Hodgkin lymfom |
| C817 | Cancer | Cancer | Lymphoma Hodgkin specificeret på anden måde |
| C819 | Cancer | Cancer | Lymphoma Hodgkin uden specifikation |
| C82 | Cancer | Cancer | Malignt lymfom af non-Hodgkin, follikulær type |
| C820 | Cancer | Cancer | Lymphoma mal non-Hodgkin follikulær small cleaved cell |
| C821 | Cancer | Cancer | Lymph mal non-Hodg foll mixed small cleaved and large cell |
| C822 | Cancer | Cancer | Lymphoma mal non Hodgkin af follikulær type large cell |
| C825 | Cancer | Cancer | Diffust follikelcenter lymfom |
| C826 | Cancer | Cancer | Kutant follikelcenter lymfom |
| C827 | Cancer | Cancer | Lymphoma mal non-Hodgkin anden af follikulær type |
| C829 | Cancer | Cancer | Lymphoma mal non-Hodgkin af follikulær type uden specifik |
| C83 | Cancer | Cancer | Malignt lymfom af non-Hodgkin af diffus type |
| C830 | Cancer | Cancer | Lymphoma mal non-Hodgkin af diffus type small cell |
| C830B | Cancer | Cancer | Lymfoplasmacytært lymfom |
| C830C | Cancer | Cancer | Nodalt marginalzonelymfom |
| C830D | Cancer | Cancer | Splenisk marginalzonelymfom |
| C831 | Cancer | Cancer | Lymphoma mal non-Hodgkin af diffus small cleaved cell |
| C831A | Cancer | Cancer | Centrocytisk lymfom |
| C831B | Cancer | Cancer | Malign lymfomatøs polypose |
| C832 | Cancer | Cancer | Lymph mal non-Hodg af diff type mixed small and large cell |
| C833 | Cancer | Cancer | Lymphoma mal non-Hodgkin af diffus type large cell |
| C833A | Cancer | Cancer | Anaplastisk diffust storcellet B-celle lymfom |
| C833B | Cancer | Cancer | CD30-positivt diffust storcellet B-celle lymfom |
| C833C | Cancer | Cancer | Centroblastært diffust storcellet B-celle lymfom |
| C833D | Cancer | Cancer | Immunoblastært diffust storcellet B-celle lymfom |
| C833E | Cancer | Cancer | Plasmablastært diffust storcellet B-celle lymfom |
| C833F | Cancer | Cancer | T-cellerigt diffust storcellet B-celle lymfom |
| C834 | Cancer | Cancer | Lymphoma mal non-Hodgkin af diffus immunoblastær type |
| C835 | Cancer | Cancer | Lymphoma mal non-Hodgkin af diffus lymfoblastær type |
| C835A | Cancer | Cancer | Lymfoblastært B-celle lymfom |
| C835B | Cancer | Cancer | Lymfoblastært T-celle lymfom |
| C835C | Cancer | Cancer | Lymfoblastært lymfom UNS |
| C836 | Cancer | Cancer | Lymphoma mal non-Hodgkin af diffus udifferentieret type |
| C837 | Cancer | Cancer | Lymphoma Burkitt |
| C837A | Cancer | Cancer | Atypisk Burkitt lymfom |
| C837B | Cancer | Cancer | Burkitt-lignende lymfom |
| C838 | Cancer | Cancer | Lymphoma mal non-Hodgkin andre diffuse typer |
| C838A | Cancer | Cancer | Primært effusionslymfom (af B-celle type) |
| C838B | Cancer | Cancer | Intravaskulært storcellet B-celle lymfom |
| C839 | Cancer | Cancer | Lymphoma mal non-Hodgkin af diffus type uden specifikation |
| C84 | Cancer | Cancer | T-celle malignt lymfom lokaliseret i hud eller systemisk |
| C840 | Cancer | Cancer | Mycosis fungoides |
| C840A | Cancer | Cancer | Mycosis fungoides assoc. m. follikulær mucinose |
| C840B | Cancer | Cancer | Pagetoid retikulose (Woringer-Kolopp) |
| C840C | Cancer | Cancer | Granulomatøs slack skin (lymphoma malignum) |
| C841 | Cancer | Cancer | Morbus Sézary |
| C842 | Cancer | Cancer | Lymphoma mal T-zone |
| C843 | Cancer | Cancer | Lymphoma mal lymfoepiteloidt |
| C844 | Cancer | Cancer | Lymphoma mal T-celle, perifert |
| C844A | Cancer | Cancer | Storcellet Pleomorft CD30+ T-celle lymfom |
| C844B | Cancer | Cancer | Storcellet Pleomorft CD30- T-celle lymfom |
| C844C | Cancer | Cancer | Storcellet immunoblastisk CD30+ T-celle lymfom |
| C844D | Cancer | Cancer | Storcellet immunoblastisk CD30- T-celle lymfom |
| C844E | Cancer | Cancer | Middel-småcellet pleomorft T-celle lymfom |
| C844F | Cancer | Cancer | Lymfoepitelioidt lymfom |
| C844H | Cancer | Cancer | Modent T-celle lymfom UNS |
| C845 | Cancer | Cancer | Lymphoma mal T-celle, anden eller uden specifikation |
| C845A | Cancer | Cancer | Storcellet anaplastisk CD30+ T-celle lymfom |
| C845B | Cancer | Cancer | Storcellet anaplastisk CD30+ T-celle lymfom, primært i huden |
| C845C | Cancer | Cancer | Subkutant pannikulitrelateret T-lymfom |
| C845D | Cancer | Cancer | Angiocentrisk T-/NK-celle lymfom |
| C846 | Cancer | Cancer | Anaplastisk storcellet lymfom, ALK-positivt |
| C846A | Cancer | Cancer | Anaplastisk storcellet lymfom, CD30-positivt |
| C847 | Cancer | Cancer | Anaplastisk storcellet lymfom, ALK-negativt |
| C848 | Cancer | Cancer | Kutant T-celle lymfom UNS |
| C849 | Cancer | Cancer | Modent NK/T-celle lymfom UNS |
| C85 | Cancer | Cancer | Malignt lymfom af non-Hodgkin type, andet og ikke spec |
| C850 | Cancer | Cancer | Lymphosarcoma |
| C851 | Cancer | Cancer | Lymphoma mal B-celle uden specifikation |
| C851A | Cancer | Cancer | Follikelcenter B-celle lymfom |
| C851B | Cancer | Cancer | Marginal zone B-celle lymfom (immunocytom) |
| C851C | Cancer | Cancer | Diffust storcellet B-celle lymfom |
| C851D | Cancer | Cancer | T-cellerigt B-celle lymfom |
| C851E | Cancer | Cancer | Storcellet intravaskulært B-celle lymfom |
| C852 | Cancer | Cancer | Mediastinalt (tymisk) storcellet B-celle lymfom |
| C857 | Cancer | Cancer | Lymphoma mal non-Hodgkin anden spec |
| C857A | Cancer | Cancer | Reticulosis lymphatica follicularis |
| C857B | Cancer | Cancer | Polymorf posttransplantations lymfoproliferativ sygd. (PTLD) |
| C859 | Cancer | Cancer | Lymphoma mal non-Hodgkin uden specifikation |
| C859A | Cancer | Cancer | Malignt lymfom uden specifikation |
| C859B | Cancer | Cancer | Lymfom uden specifikation |
| C86 | Cancer | Cancer | Andre NK/T-celle lymfomer |
| C860 | Cancer | Cancer | Ekstranodalt NK/T-celle lymfom, nasal type |
| C861 | Cancer | Cancer | Hepatosplenisk T-celle lymfom |
| C862 | Cancer | Cancer | Enteropati-type (intestinalt) T-celle lymfom |
| C863 | Cancer | Cancer | Subkutant panniculitis-lignende T-celle lymfom |
| C864 | Cancer | Cancer | Blastisk NK-celle lymfom |
| C865 | Cancer | Cancer | Angioimmunoblastært T-celle lymfom |
| C866 | Cancer | Cancer | Primært kutane CD30-positive T-celle proliferationer |
| C866A | Cancer | Cancer | Lymfomatoid papulose |
| C866B | Cancer | Cancer | Primært kutant anaplastisk storcellet lymfom |
| C866C | Cancer | Cancer | Primært kutant CD30-positivt storcellet lymfom |
| C88 | Cancer | Cancer | Ondartet immunoproliferativ sygdom |
| C880 | Cancer | Cancer | Macroglobulinaemia primaria Waldenström |
| C881 | Cancer | Cancer | Paraproteinaemia alpha heavy chain |
| C882 | Cancer | Cancer | Paraproteinaemia gamma heavy chain |
| C882A | Cancer | Cancer | Gamma heavy chain disease |
| C882B | Cancer | Cancer | My heavy chain disease |
| C883 | Cancer | Cancer | Neopl mal immunoproliferativum intestini tenuis |
| C883A | Cancer | Cancer | Alpha heavy chain disease |
| C883B | Cancer | Cancer | Middelhavs lymfom |
| C884 | Cancer | Cancer | Ekstranodalt marginalzone B-celle lymfom |
| C884A | Cancer | Cancer | Lymfom af mucosa-associeret lymfoidt væv (MALT-lymfom) |
| C884B | Cancer | Cancer | Lymfom af hud-associeret lymfoidt væv (SALT-lymfom) |
| C884C | Cancer | Cancer | Lymfom af bronkie-associeret lymfoidt væv (BALT-lymfom) |
| C887 | Cancer | Cancer | Neopl mal immunoproliferativum andre former |
| C889 | Cancer | Cancer | Neopl mal immunoproliferativum uden specifikation |
| C90 | Cancer | Cancer | Myelomatose, multiple myelomer |
| C900 | Cancer | Cancer | Myelomatosis |
| C901 | Cancer | Cancer | Leukaemia plasmacytica |
| C902 | Cancer | Cancer | Plasmacytoma extramedullare |
| C902A | Cancer | Cancer | Myeloma solitarium |
| C903 | Cancer | Cancer | Solitært ossøst plasmacytom |
| C91 | Cancer | Cancer | Lymfatisk leukæmi |
| C910 | Cancer | Cancer | Leukaemia lymphoblastica acuta |
| C911 | Cancer | Cancer | Leukaemia lymphatica chronica |
| C911A | Cancer | Cancer | Lymfoplasmacytær leukæmi |
| C911B | Cancer | Cancer | Richter syndrom |
| C912 | Cancer | Cancer | Leukaemia lymphatica subacuta |
| C913 | Cancer | Cancer | Leukaemia prolymphocytica |
| C914 | Cancer | Cancer | Leukaemia hårcelle |
| C914A | Cancer | Cancer | Reticuloendotheliosis leucaemica |
| C915 | Cancer | Cancer | Leukaemia T-lymfocytær hos voksne |
| C915A | Cancer | Cancer | Adult T-celle lymfom/leukæmi (HTLV-1-assoc.), akut variant |
| C915B | Cancer | Cancer | Adult T-celle lymfom/leukæmi (HTLV-1-assoc.), kron. variant |
| C915C | Cancer | Cancer | Adult T-celle lymfom/leukæmi (HTLV-1-assoc), lymfomatoid var |
| C915D | Cancer | Cancer | Adult T-celle lymfom/leukæmi (HTLV-1-assoc), smouldering var |
| C916 | Cancer | Cancer | Prolymfocyt leukæmi af T-celle type |
| C917 | Cancer | Cancer | Leukaemia lymphatica anden form |
| C917A | Cancer | Cancer | Leukaemia aleukaemica lymphatica |
| C917B | Cancer | Cancer | Large granular T-celle lymfocytær leukæmi |
| C918 | Cancer | Cancer | Moden B-celle leukæmi af Burkitt-type |
| C919 | Cancer | Cancer | Leukaemia lymphatica uden specifikation |
| C92 | Cancer | Cancer | Myeloid leukæmi |
| C920 | Cancer | Cancer | Leukaemia myeloblastica acuta |
| C920A | Cancer | Cancer | Akut myeloblastær leukæmi med minimal differentiering |
| C920B | Cancer | Cancer | Akut myeloblastær leukæmi med modning |
| C920C | Cancer | Cancer | Akut myeloblastær leukæmi, AML1/ETO |
| C920D | Cancer | Cancer | Akut myeloblastær leukæmi, AML M0 |
| C920E | Cancer | Cancer | Akut myeloblastær leukæmi, AML M1 |
| C920F | Cancer | Cancer | Akut myeloblastær leukæmi, AML M2 |
| C920G | Cancer | Cancer | Akut myeloblastær leukæmi, AML med t(8 |
| C920H | Cancer | Cancer | Akut myeloblastær leukæmi UNS (uden FAB-klassificering) |
| C921 | Cancer | Cancer | Leukaemia myeloides chronica |
| C921A | Cancer | Cancer | Kronisk myeloid leukæmi med blastkrise |
| C922 | Cancer | Cancer | Leukaemia myeloides subacuta |
| C923 | Cancer | Cancer | Sarcoma myeloides |
| C923A | Cancer | Cancer | Chloroma |
| C923B | Cancer | Cancer | Sarcoma granulocytica |
| C924 | Cancer | Cancer | Leukaemia promyelocytica |
| C924A | Cancer | Cancer | Akut myeloblastær leukæmi M3 med t(15 |
| C925 | Cancer | Cancer | Leukaemia myelomonocytica acuta |
| C925A | Cancer | Cancer | Akut myeloblastær leukæmi M4 Eo med inv(16) eller t(16 |
| C926 | Cancer | Cancer | Akut myeloblastær leukæmi med 11q23-abnormalitet |
| C926A | Cancer | Cancer | Akut myeloblastær leukæmi med variation af MLL-gen |
| C927 | Cancer | Cancer | Leukaemia myeloides anden form |
| C927A | Cancer | Cancer | Leukaemia aleukaemica myeloides |
| C927B | Cancer | Cancer | Kronisk neutrofil leukæmi |
| C928 | Cancer | Cancer | Akut myeloid leukæmi med multilinje dysplasi |
| C929 | Cancer | Cancer | Leukaemia myeloides uden specifikation |
| C93 | Cancer | Cancer | Monocytleukæmi |
| C930 | Cancer | Cancer | Leukaemia monocytica acuta |
| C930A | Cancer | Cancer | Akut monoblastær, AML M5a |
| C930B | Cancer | Cancer | Akut monoblastær, AML M5b |
| C931 | Cancer | Cancer | Leukaemia monocytica chronica |
| C931A | Cancer | Cancer | Kronisk myelomonocytær leukæmi, CMML-1 |
| C931B | Cancer | Cancer | Kronisk myelomonocytær leukæmi, CMML-2 |
| C931C | Cancer | Cancer | Kronisk myelomonocytær, CMML med eosinofili |
| C932 | Cancer | Cancer | Leukaemia monocytica subacuta |
| C933 | Cancer | Cancer | Juvenil myelomonocytær leukæmi |
| C937 | Cancer | Cancer | Leukaemia monocytica anden form |
| C939 | Cancer | Cancer | Leukaemia monocytica uden specifikation |
| C94 | Cancer | Cancer | Leukæmi, anden spec |
| C940 | Cancer | Cancer | Erythroleukaemia acuta |
| C940A | Cancer | Cancer | Erytroleukæmi UNS |
| C941 | Cancer | Cancer | Erythroleukaemia chronica |
| C942 | Cancer | Cancer | Leukaemia megakaryoblastica acuta |
| C943 | Cancer | Cancer | Leukaemia basophilica |
| C943A | Cancer | Cancer | Mastcelleleukæmi |
| C944 | Cancer | Cancer | Panmyelosis acuta |
| C945 | Cancer | Cancer | Myelofibrosis acuta |
| C946 | Cancer | Cancer | Uklassificerbar myelodysplasi/myeloproliferativ sygdom |
| C947 | Cancer | Cancer | Leukaemia anden spec form |
| C947A | Cancer | Cancer | Leukaemia eosinophilica |
| C947B | Cancer | Cancer | Aggressiv NK-celle leukæmi |
| C947C | Cancer | Cancer | Akut basofil leukæmi |
| C95 | Cancer | Cancer | Leukæmi uden specifikation |
| C950 | Cancer | Cancer | Leukaemia acuta uden specifikation |
| C950A | Cancer | Cancer | Blastcelleleukæmi |
| C950B | Cancer | Cancer | Leukaemia acuta, stamcelleleukæmi |
| C950C | Cancer | Cancer | Akut bilineær leukæmi |
| C950D | Cancer | Cancer | Akut leukæmi af blandet linearitet |
| C951 | Cancer | Cancer | Leukaemia chronica uden specifikation |
| C952 | Cancer | Cancer | Leukaemia subacuta uden specifikation |
| C957 | Cancer | Cancer | Leukaemia anden form uden specifikation |
| C959 | Cancer | Cancer | Leukaemia uden specifikation |
| C96 | Cancer | Cancer | Kræft, anden og ikke spec i lymfatisk og bloddannende væv |
| C961 | Cancer | Cancer | Histiocytosis maligna |
| C962 | Cancer | Cancer | Mastocytoma mal |
| C962B | Cancer | Cancer | Mastcelle sarkom |
| C963 | Cancer | Cancer | Lymphoma mal histiocyticum verum |
| C964 | Cancer | Cancer | Dendritcelle sarkom (accessoriske celler) |
| C964A | Cancer | Cancer | Interdigiterende dendritcelle sarkom |
| C964B | Cancer | Cancer | Langerhans-celle sarkom |
| C964C | Cancer | Cancer | Follikulært dendritcelle sarkom |
| C967 | Cancer | Cancer | Neopl mal aliud telae lymphaticae et haematopoieticae |
| C967A | Cancer | Cancer | Myelosarkom |
| C968 | Cancer | Cancer | Malign histiocytose UNS |
| C969 | Cancer | Cancer | Neopl mal telae lymphaticae et haematopoieticae uden spec |
| C97 | Cancer | Cancer | Kræft opstået uafhængigt i flere lokalisationer |
| C979 | Cancer | Cancer | Neopl mal primarium flere lokalisationer |
| D00 | Cancer | Cancer | Carc in situ på læbe, i mundhule, spiserør og mavesæk |
| D000 | Cancer | Cancer | Carc in situ labii oris, cavi oris et pharyngis |
| D000A | Cancer | Cancer | Carc in situ cavi oris |
| D000B | Cancer | Cancer | Carc in situ labii oris |
| D000C | Cancer | Cancer | Carc in situ pharyngis |
| D001 | Cancer | Cancer | Carc in situ oesophagi |
| D002 | Cancer | Cancer | Carc in situ ventriculi |
| D01 | Cancer | Cancer | Carc in situ i andre og ikke spec fordøjelsesorganer |
| D010 | Cancer | Cancer | Carc in situ coli |
| D011 | Cancer | Cancer | Carc in situ rectosigmoidei |
| D012 | Cancer | Cancer | Carc in situ recti |
| D013 | Cancer | Cancer | Carc in situ ani et canalis analis |
| D013A | Cancer | Cancer | Carc in situ ani |
| D013B | Cancer | Cancer | Carc in situ canalis analis |
| D014 | Cancer | Cancer | Carc in situ intestini alii uden specifikation |
| D014A | Cancer | Cancer | Carcinoma in situ i tyndtarmen |
| D015 | Cancer | Cancer | Carc in situ hepatis, vesicae felleae et viarum biliarium |
| D015A | Cancer | Cancer | Carc in situ ampullae Vateri |
| D015B | Cancer | Cancer | Carc in situ hepatis |
| D015C | Cancer | Cancer | Carc in situ vesicae felleae |
| D015D | Cancer | Cancer | Carc in situ viarum biliarium |
| D017 | Cancer | Cancer | Carc in situ systematis digestivi m anden lokalisation |
| D017A | Cancer | Cancer | Carcinoma in situ i bugspytkirtlen |
| D019 | Cancer | Cancer | Carc in situ systematis digestivi uden specifikation |
| D02 | Cancer | Cancer | Carc in situ i mellemøre og åndedrætsorganer |
| D020 | Cancer | Cancer | Carc in situ laryngis |
| D021 | Cancer | Cancer | Carc in situ tracheae |
| D022 | Cancer | Cancer | Carc in situ bronchi et pulmonis |
| D022A | Cancer | Cancer | Carc in situ bronchi |
| D022B | Cancer | Cancer | Carc in situ pulmonis |
| D023 | Cancer | Cancer | Carc in situ i andre dele af åndedrætsorganer samt mellemør |
| D023A | Cancer | Cancer | Carc in situ auris mediae |
| D023B | Cancer | Cancer | Carc in situ cavi nasi |
| D023C | Cancer | Cancer | Carc in situ sinus accessorii |
| D024 | Cancer | Cancer | Carc in situ organorum respiratorii uden specifikation |
| D03 | Cancer | Cancer | Melanoma in situ |
| D030 | Cancer | Cancer | Melanoma in situ labii oris |
| D030E | Cancer | Cancer | Melanoma in situ labii oris, lentigo maligna |
| D030F | Cancer | Cancer | Melanoma in situ labii oris, superficielt spredende |
| D031 | Cancer | Cancer | Melanoma in situ palpebrae incl. canthus oculi |
| D031A | Cancer | Cancer | Melanoma in situ canthi oculi |
| D031B | Cancer | Cancer | Melanoma in situ palpebrae |
| D031E | Cancer | Cancer | Melanoma in situ palpebrae, lentigo maligna |
| D031F | Cancer | Cancer | Melanoma in situ palpebrae, superficielt spredende |
| D032 | Cancer | Cancer | Melanoma in situ auris, can auris ext et meatus acustici ex |
| D032A | Cancer | Cancer | Melanoma in situ auris |
| D032B | Cancer | Cancer | Melanoma in situ canalis auris externae |
| D032C | Cancer | Cancer | Melanoma in situ meatus acustici externi |
| D032E | Cancer | Cancer | Melanoma in situ auris, canalis auris externae et meatus acustici externi, lentigo maligna |
| D032F | Cancer | Cancer | Melanoma in situ auris, canalis auris externae et meatus acustici externi, superficielt spredende |
| D033 | Cancer | Cancer | Melanoma in situ faciei m anden eller ikke spec lokalisatio |
| D033A | Cancer | Cancer | Melanoma in situ på næsen |
| D033E | Cancer | Cancer | Melanoma in situ faciei med anden eller ikke specificeret lokalisation, lentigo maligna |
| D033F | Cancer | Cancer | Melanoma in situ faciei med anden eller ikke specificeret lokalisation, superficielt spredende |
| D034 | Cancer | Cancer | Melanoma in situ capillitii et colli |
| D034A | Cancer | Cancer | Melanoma in situ capillitii |
| D034B | Cancer | Cancer | Melanoma in situ colli |
| D034E | Cancer | Cancer | Melanoma in situ capillitii et colli, lentigo maligna |
| D034F | Cancer | Cancer | Melanoma in situ capillitii et colli, superficielt spredende |
| D035 | Cancer | Cancer | Melanoma in situ trunci |
| D035A | Cancer | Cancer | Melanoma in situ cutis ani |
| D035B | Cancer | Cancer | Melanoma in situ cutis mammae |
| D035C | Cancer | Cancer | Melanoma in situ cutis perianalis |
| D035D | Cancer | Cancer | Melanoma in situ cervicis uteri |
| D035E | Cancer | Cancer | Melanoma in situ trunci, lentigo maligna |
| D035F | Cancer | Cancer | Melanoma in situ trunci, superficielt spredende |
| D035G | Cancer | Cancer | Melanoma in situ i vulva |
| D036 | Cancer | Cancer | Melanoma in situ extr superioris incl. cingulum |
| D036A | Cancer | Cancer | Melanoma in situ cinguli |
| D036B | Cancer | Cancer | Melanoma in situ extremitatis superioris |
| D036E | Cancer | Cancer | Melanoma in situ extremitatis superioris, lentigo maligna |
| D036F | Cancer | Cancer | Melanoma in situ extremitatis superioris, superficielt spredende |
| D037 | Cancer | Cancer | Melanoma in situ extr inferioris incl. regio coxae |
| D037A | Cancer | Cancer | Melanoma in situ extremitatis inferioris |
| D037B | Cancer | Cancer | Melanoma in situ regionis coxae |
| D037E | Cancer | Cancer | Melanoma in situ extremitatis inferioris, lentigo maligna |
| D037F | Cancer | Cancer | Melanoma in situ extremitatis inferioris, superficielt spredende |
| D038 | Cancer | Cancer | Melanoma in situ m anden lokalisation |
| D038E | Cancer | Cancer | Melanoma in situ med anden lokalisation, lentigo maligna |
| D038F | Cancer | Cancer | Melanoma in situ med anden lokalisation, superficielt spredende |
| D039 | Cancer | Cancer | Melanoma in situ uden specifikation |
| D039E | Cancer | Cancer | Melanoma in situ uden specifikation, lentigo maligna |
| D039F | Cancer | Cancer | Melanoma in situ uden specifikation, superficielt spredende |
| D05 | Cancer | Cancer | Carc in situ i bryst |
| D050 | Cancer | Cancer | Carc in situ mammae lobularis |
| D051 | Cancer | Cancer | Carc in situ mammae intraductalis |
| D056 | Cancer | Cancer | Pagets sygdom i bryst (brystvorte) |
| D057 | Cancer | Cancer | Carc in situ mammae m anden lokalisation |
| D059 | Cancer | Cancer | Carc in situ mammae uden specifikation |
| D06 | Cancer | Cancer | Carc in situ i livmoderhals |
| D060 | Cancer | Cancer | Carc in situ endocervicis uteri |
| D061 | Cancer | Cancer | Carc in situ exocervicis uteri |
| D067 | Cancer | Cancer | Carc in situ cervicis uteri m anden lokalisation |
| D069 | Cancer | Cancer | Carc in situ cervicis uteri uden specifikation |
| D07 | Cancer | Cancer | Carc in situ i andre kønsorganer |
| D070 | Cancer | Cancer | Carc in situ endometrii |
| D071 | Cancer | Cancer | Carc in situ vulvae |
| D071A | Cancer | Cancer | Vulvær intraepitelial neoplasi (VIN), grad III |
| D071D | Cancer | Cancer | Carcinoma in situ vulvae, mb. Bowen |
| D071E | Cancer | Cancer | Carcinoma in situ vulvae, bowenoid papulose |
| D071Z | Cancer | Cancer | Carcinoma in situ vulvae, andet |
| D072 | Cancer | Cancer | Carc in situ vaginae |
| D072A | Cancer | Cancer | Vaginal intraepitelial neoplasi (VAIN), grad III |
| D073 | Cancer | Cancer | Carc in situ gen fem m anden eller ikke spec lokalisation |
| D073A | Cancer | Cancer | Carcinoma in situ i æggestok |
| D073B | Cancer | Cancer | Carcinoma in situ m. an. lokalisation i kvindeligt kønsorgan |
| D073C | Cancer | Cancer | Carcinoma in situ i kvindeligt kønsorgan UNS |
| D074 | Cancer | Cancer | Carc in situ penis |
| D074D | Cancer | Cancer | Carcinoma in situ penis, mb. Bowen |
| D074E | Cancer | Cancer | Carcinoma in situ penis, bowenoid papulose |
| D074Z | Cancer | Cancer | Carcinoma in situ penis, andet |
| D075 | Cancer | Cancer | Carc in situ prostatae |
| D076 | Cancer | Cancer | Carc in situ gen viri m anden eller ikke spec lokalisation |
| D076A | Cancer | Cancer | Carc in situ genitalium viri, anden lokalisation |
| D076B | Cancer | Cancer | Carc in situ genitalium viri uden specifikation |
| D076T | Cancer | Cancer | Carcinoma in situ testis |
| D09 | Cancer | Cancer | Carc in situ i andre eller ikke spec lokalisationer |
| D090 | Cancer | Cancer | Carc in situ vesicae urinariae |
| D091 | Cancer | Cancer | Carc in situ i andre og ikke spec urinorganer |
| D091A | Cancer | Cancer | Carcinoma in situ i nyre |
| D091B | Cancer | Cancer | Carcinoma in situ i nyrebækken |
| D091C | Cancer | Cancer | Carcinoma in situ i urinleder |
| D091D | Cancer | Cancer | Carcinoma in situ i urinrør |
| D092 | Cancer | Cancer | Carc in situ oculi |
| D093 | Cancer | Cancer | Carc in situ glandulae thyr et glandulae endocrinae aliae |
| D093A | Cancer | Cancer | Carc in situ glandulae thyreoideae |
| D093B | Cancer | Cancer | Carc in situ glandulae endocrinae aliae |
| D097 | Cancer | Cancer | Carc in situ m anden spec lokalisation |
| D097A | Cancer | Cancer | Carcinoma in situ på næsen UNS |
| D099 | Cancer | Cancer | Carc in situ uden specifikation |
| D50 | Non-traumatic bleedings | Possible bleedings | Jernmangelanæmi |
| D500 | Non-traumatic bleedings | Remaining bleedings | Blødningsanæmi kronisk |
| D508 | Non-traumatic bleedings | Possible bleedings | Jernmangelanæmi af anden årsag |
| D508A | Non-traumatic bleedings | Possible bleedings | Jernmangelanæmi forårsaget af insufficient indtag af jern |
| D509 | Non-traumatic bleedings | Possible bleedings | Jernmangelanæmi uden specifikation |
| D509A | Non-traumatic bleedings | Possible bleedings | Anæmia hypochromica |
| D62 | Non-traumatic bleedings | Remaining bleedings | Akut anæmi efter blødning |
| D629 | Non-traumatic bleedings | Remaining bleedings | Akut anæmi efter blødning |
| D63 | Non-traumatic bleedings | Possible bleedings | Anæmi ved kroniske sygdomme klassificeret andetsteds |
| D638 | Non-traumatic bleedings | Possible bleedings | Anæmi ved andre kroniske sygdomme klassificeret andetsteds |
| D64 | Non-traumatic bleedings | Possible bleedings | Anæmier, andre |
| D648 | Non-traumatic bleedings | Possible bleedings | Anæmi, anden specificeret |
| D649 | Non-traumatic bleedings | Possible bleedings | Anæmi uden specifikation |
| D698 | Non-traumatic bleedings | Remaining bleedings | Tilstande m blødningstendens, andre specificerede |
| D699 | Non-traumatic bleedings | Remaining bleedings | Blødningstendens uden specifikation |
| D733 | Bacterial infections | Remaining infections | Abscessus lienis |
| D735 | Thromboembolic conditions | Remaining thromboembolisms | Infarctus lienis |
| D738D | Bacterial infections | Remaining infections | Splenitis uden specifikation |
| E06 | Bacterial infections | Remaining infections | Betændelse i skjoldbruskkirtlen |
| E060 | Bacterial infections | Remaining infections | Betændelse i skjoldbruskkirtlen, akut |
| E060A | Bacterial infections | Remaining infections | Abscessus glandulae thyreoideae |
| E060B | Bacterial infections | Remaining infections | Thyreoiditis suppurativa |
| E061 | Bacterial infections | Remaining infections | Betændelse i skjoldbruskkirtlen, subakut |
| E062 | Bacterial infections | Remaining infections | Betændelse i skjoldbruskkirtlen, kronisk med tyreotoksokose |
| E065 | Bacterial infections | Remaining infections | Betændelse i skjoldbruskkirtlen, anden kronisk |
| E069 | Bacterial infections | Remaining infections | Betændelse i skjoldbruskkirtlen uden specifikation |
| E078B | Non-traumatic bleedings | Remaining bleedings | Haemorrhagia glandulae thyreoideae |
| E078C | Thromboembolic conditions | Remaining thromboembolisms | Infarctus glandulae thyreoideae |
| E236A | Bacterial infections | Remaining infections | Abscessus hypophyseos |
| E321 | Bacterial infections | Remaining infections | Byld i thymus |
| G00 | Bacterial infections | Infections in the CNS | Hjernehindebetændelse forårsaget af bakterie |
| G000 | Bacterial infections | Infections in the CNS | Meningitis, haemophilus influenzae |
| G001 | Bacterial infections | Infections in the CNS | Meningitis pneumococcica |
| G002 | Bacterial infections | Infections in the CNS | Meningitis streptococcica |
| G003 | Bacterial infections | Infections in the CNS | Meningitis staphylococcica |
| G008 | Bacterial infections | Infections in the CNS | Meningitis som følge af anden bakterie |
| G008A | Bacterial infections | Infections in the CNS | Meningitis, Klebsiella |
| G008B | Bacterial infections | Infections in the CNS | Meningitis, Escherichia coli |
| G009 | Bacterial infections | Infections in the CNS | Meningitis bakteriel, uden specifikation |
| G009A | Bacterial infections | Infections in the CNS | Meningitis purulenta |
| G01 | Bacterial infections | Infections in the CNS | Hjernehindebetændelse ved bakterielle sygd klass ansted |
| G019 | Bacterial infections | Infections in the CNS | Meningitis ved bakterielle sygdom klassificeret andetsteds |
| G019A | Bacterial infections | Infections in the CNS | Meningitis, sekundær syfilis |
| G019B | Bacterial infections | Infections in the CNS | Meningitis, tyfus |
| G019C | Bacterial infections | Infections in the CNS | Meningitis salmonellosa |
| G019E | Bacterial infections | Infections in the CNS | Meningitis, kongenit syfilis |
| G019F | Bacterial infections | Infections in the CNS | Meningitis, neurosyfilis |
| G019G | Bacterial infections | Infections in the CNS | Meningitis, leptospirosis |
| G019H | Bacterial infections | Infections in the CNS | Meningitis gonococcica |
| G019I | Bacterial infections | Infections in the CNS | Meningitis, Listeria |
| G019J | Bacterial infections | Infections in the CNS | Meningitis, Lyme's sygdom |
| G02 | Bacterial infections | Infections in the CNS | Hjernehindebet ved infek og parasit sygd klass ansted |
| G020 | Bacterial infections | Infections in the CNS | Meningitis ved viral sygdom klassificeret andetsteds |
| G028 | Bacterial infections | Infections in the CNS | Meningitis ved andre infek og parasit sygd klass ansted |
| G03 | Bacterial infections | Infections in the CNS | Hjernehindebetændelse af anden årsag og uden specifikation |
| G04 | Bacterial infections | Infections in the CNS | Hjerne- og rygmarvsbetændelse |
| G040 | Bacterial infections | Infections in the CNS | Encephalitis disseminata acuta |
| G040A | Bacterial infections | Infections in the CNS | Encephalomyelitis efter vaccination |
| G041 | Bacterial infections | Infections in the CNS | Paraplegia spastica tropica |
| G042 | Bacterial infections | Infections in the CNS | Meningoencephalitis et -myelitis bact ikke klass ansted |
| G042A | Bacterial infections | Infections in the CNS | Meningomyelitis bacterialis, ikke klassificeret andetsteds |
| G042B | Bacterial infections | Infections in the CNS | Bakteriel meningoencefalitis IKA |
| G048 | Bacterial infections | Infections in the CNS | Encephalitis, myelitis et encephalomyelitis, anden |
| G048A | Bacterial infections | Infections in the CNS | Encephalitis postinfectiosa ikke klassificeret andetsteds |
| G048B | Bacterial infections | Infections in the CNS | Encephalomyelitis postinfectiosa ikke klass andetsteds |
| G048C | Bacterial infections | Infections in the CNS | Myelitis postinfectiosa ikke klassificeret andetsteds |
| G049 | Bacterial infections | Infections in the CNS | Encephalitis, myelitis et encephalomyelitis uden specifik |
| G049A | Bacterial infections | Infections in the CNS | Encephalitis uden specifikation |
| G049B | Bacterial infections | Infections in the CNS | Encephalomyelitis uden specifikation |
| G049C | Bacterial infections | Infections in the CNS | Myelitis uden specifikation |
| G049D | Bacterial infections | Infections in the CNS | Cerebral ventrikulitis UNS |
| G05 | Bacterial infections | Infections in the CNS | Hjerne- og rygmarvsbetændelse ved sygdom klass ansted |
| G050 | Bacterial infections | Infections in the CNS | Encephalitis og encephmyelitis ved bakt sygd klass ansted |
| G050B | Bacterial infections | Infections in the CNS | Encephalitis tuberculosa |
| G050D | Bacterial infections | Infections in the CNS | Encephalitis, listeria |
| G050E | Bacterial infections | Infections in the CNS | Encephalitis meningococcica |
| G050F | Bacterial infections | Infections in the CNS | Encephalomyelitis tuberculosa |
| G050H | Bacterial infections | Infections in the CNS | Encephalomyelitis, listeria |
| G050I | Bacterial infections | Infections in the CNS | Encephalomyelitis meningococcica |
| G050K | Bacterial infections | Infections in the CNS | Myelitis, listeria |
| G050L | Bacterial infections | Infections in the CNS | Myelitis, sensyfilitisk |
| G050N | Bacterial infections | Infections in the CNS | Myelitis tuberculosa |
| G050O | Bacterial infections | Infections in the CNS | Myelitis meningococcica |
| G050P | Bacterial infections | Infections in the CNS | Encefalitis ved bakteriel sygdom klassificeret andetsteds |
| G050R | Bacterial infections | Infections in the CNS | Encefalomyelitis ved bakteriel sygdom klas. andetsteds |
| G050S | Bacterial infections | Infections in the CNS | Myelitis ved bakteriel sygdom klassificeret andetsteds |
| G051 | Bacterial infections | Infections in the CNS | Encephalitis og encephalomyel ved viral sygd klass ansted |
| G052 | Bacterial infections | Infections in the CNS | Encephalitis og encephmyelitis ved infek sygd klass ansted |
| G052K | Bacterial infections | Infections in the CNS | Encefalitis ved anden infektiøs eller parasitær sygdom KA |
| G052L | Bacterial infections | Infections in the CNS | Myelitis ved anden infektiøs eller parasitær sygdom KA |
| G052M | Bacterial infections | Infections in the CNS | Encefalomyelitis ved anden infektiøs el. parasitær sygdom KA |
| G052N | Bacterial infections | Infections in the CNS | Meningoencefalitis ved anden infektiøs/parasitær sygdom KA |
| G058 | Bacterial infections | Infections in the CNS | Encephalitis og encephmyelitis ved andre sygd klass ansted |
| G06 | Bacterial infections | Infections in the CNS | Byld og granulom i kranie og rygmarvskanal |
| G060 | Bacterial infections | Infections in the CNS | Abscessus et granuloma intracraniale |
| G060A | Bacterial infections | Infections in the CNS | Abscessus intracranialis extraduralis |
| G060B | Bacterial infections | Infections in the CNS | Abscessus intracranialis subduralis |
| G060C | Bacterial infections | Infections in the CNS | Abscessus otogenicus |
| G060D | Bacterial infections | Infections in the CNS | Abscessus intracranialis epiduralis |
| G060E | Bacterial infections | Infections in the CNS | Abscessus cerebelli |
| G060F | Bacterial infections | Infections in the CNS | Abscessus cerebri |
| G060G | Bacterial infections | Infections in the CNS | Granuloma cerebellaris |
| G060H | Bacterial infections | Infections in the CNS | Granuloma intracraniale subdurale |
| G060I | Bacterial infections | Infections in the CNS | Granuloma otogenicum |
| G060J | Bacterial infections | Infections in the CNS | Granuloma cerebralis |
| G060K | Bacterial infections | Infections in the CNS | Granuloma intracraniale |
| G060L | Bacterial infections | Infections in the CNS | Granuloma intracraniale epidurale |
| G060M | Bacterial infections | Infections in the CNS | Granuloma intracraniale extradurale |
| G061 | Bacterial infections | Infections in the CNS | Abscessus et granuloma intraspinale |
| G061A | Bacterial infections | Infections in the CNS | Abscessus intraspinalis subduralis |
| G061B | Bacterial infections | Infections in the CNS | Abscessus intraspinalis extraduralis |
| G061C | Bacterial infections | Infections in the CNS | Abscessus intraspinalis epiduralis |
| G061D | Bacterial infections | Infections in the CNS | Granuloma intraspinale extradurale |
| G061E | Bacterial infections | Infections in the CNS | Granuloma intraspinale epidurale |
| G061F | Bacterial infections | Infections in the CNS | Granuloma intraspinale |
| G061G | Bacterial infections | Infections in the CNS | Granuloma intraspinale subdurale |
| G062 | Bacterial infections | Infections in the CNS | Abscessus epiduralis et subduralis uden specifikation |
| G062A | Bacterial infections | Infections in the CNS | Abscessus epiduralis uden specifikation |
| G062B | Bacterial infections | Infections in the CNS | Abscessus extraduralis uden specifikation |
| G062C | Bacterial infections | Infections in the CNS | Abscessus subduralis uden specifikation |
| G07 | Bacterial infections | Infections in the CNS | Byld i kranie og rygmarvskanal ved sygd klass ansted |
| G079 | Bacterial infections | Infections in the CNS | Abscessus intracraniale et intraspin ved sygd klass ansted |
| G079A | Bacterial infections | Infections in the CNS | Abscessus intraspinalis ved sygd klassificeret andetsteds |
| G079B | Bacterial infections | Infections in the CNS | Abscessus intracranialis ved sygd klassificeret andetsteds |
| G079C | Bacterial infections | Infections in the CNS | Abscessus cerebri tuberculosus |
| G079D | Bacterial infections | Infections in the CNS | Abscessus cerebri, amøbicus |
| G079E | Bacterial infections | Infections in the CNS | Abscessus cerebri gonococcicus |
| G079F | Bacterial infections | Infections in the CNS | Granuloma cerebralis, schistosomiasis |
| G079G | Bacterial infections | Infections in the CNS | Tuberculoma cerebri |
| G079H | Bacterial infections | Infections in the CNS | Tuberculoma meninges |
| G079J | Bacterial infections | Infections in the CNS | Intrakranielt granulom ved sygdom klassificeret andetsteds |
| G079K | Bacterial infections | Infections in the CNS | Intraspinalt granulom ved sygdom klassificeret andetsteds |
| G08 | Thromboembolic conditions | Thromboembolisms in the CNS | Årebetændelse og blodprop i kranie og rygmarvskanal |
| G089 | Thromboembolic conditions | Thromboembolisms in the CNS | Phlebitis et thrombophlebitis intracranialis et intraspin |
| G089A | Thromboembolic conditions | Thromboembolisms in the CNS | Embolia intraspinalis pyogenica |
| G089B | Thromboembolic conditions | Thromboembolisms in the CNS | Embolia intracranialis pyogenica |
| G089C | Other vascular conditions | Remaining other vascular conditions | Endophlebitis intraspinalis pyogenica |
| G089D | Other vascular conditions | Remaining other vascular conditions | Endophlebitis intracranialis pyogenica |
| G089E | Other vascular conditions | Remaining other vascular conditions | Phlebitis intraspinalis pyogenica |
| G089F | Other vascular conditions | Remaining other vascular conditions | Phlebitis sinus cerebri pyogenica |
| G089G | Other vascular conditions | Remaining other vascular conditions | Phlebitis intracranialis pyogenica |
| G089H | Thromboembolic conditions | Thromboembolisms in the CNS | Thrombophlebitis sinuum durae matris pyogenica |
| G089I | Thromboembolic conditions | Thromboembolisms in the CNS | Thrombophlebitis cerebralis pyogenica |
| G089J | Thromboembolic conditions | Thromboembolisms in the CNS | Thrombophlebitis intracranialis pyogenica |
| G089K | Thromboembolic conditions | Thromboembolisms in the CNS | Thrombophlebitis intraspinalis pyogenica |
| G089L | Thromboembolic conditions | Thromboembolisms in the CNS | Thrombosis sinus cerebri pyogenica |
| G089M | Thromboembolic conditions | Thromboembolisms in the CNS | Thrombosis intracranialis pyogenica |
| G089N | Thromboembolic conditions | Thromboembolisms in the CNS | Thrombosis intraspinalis pyogenica |
| G361 | Bacterial infections | Infections in the CNS | Leukoencephalitis haemorrhagica acuta et subacuta |
| G361A | Bacterial infections | Infections in the CNS | Leukoencephalitis haemorrhagica subacuta |
| G361B | Bacterial infections | Infections in the CNS | Leukoencephalitis haemorrhagica acuta |
| G373 | Bacterial infections | Infections in the CNS | Myelitis tranversa acuta ved demyeliniserende sygd i CNS |
| G373A | Bacterial infections | Infections in the CNS | Myelitis tranversa acuta uden specifikation |
| G374 | Bacterial infections | Infections in the CNS | Myelitis necroticans subacuta |
| G433A | Thromboembolic conditions | Thromboembolisms in the CNS | Hemicrania med cerebralt infarkt |
| G45 | Other vascular conditions | Other vascular conditions in the CNS | Utilstrækkelig blodforsyning i hjernen og beslægtede syndr |
| G450 | Other vascular conditions | Other vascular conditions in the CNS | Arteria vertebro-basilaris syndrom |
| G450A | Other vascular conditions | Other vascular conditions in the CNS | Arteria vertebralis syndrom |
| G450B | Other vascular conditions | Other vascular conditions in the CNS | Arteria basilaris syndrom |
| G451 | Other vascular conditions | Other vascular conditions in the CNS | Arteria carotis syndrom |
| G452 | Other vascular conditions | Other vascular conditions in the CNS | Arteria precerebralis multiplex et bilateralis syndrom |
| G452A | Other vascular conditions | Other vascular conditions in the CNS | Arteria praecerebralis bilateralis syndrom |
| G453 | Other vascular conditions | Other vascular conditions in the CNS | Amaurosis fugax |
| G458 | Other vascular conditions | Other vascular conditions in the CNS | Ischaemia cerebri trans og beslægtede syndromer, andre |
| G459 | Other vascular conditions | Other vascular conditions in the CNS | Ischaemia cerebri transitoria uden specifikation |
| G460 | Other vascular conditions | Other vascular conditions in the CNS | Arteria cerebri media syndrom |
| G461 | Other vascular conditions | Other vascular conditions in the CNS | Arteria cerebri anterior syndrom |
| G462 | Other vascular conditions | Other vascular conditions in the CNS | Arteria cerebri posterior syndrom |
| G463 | Thromboembolic conditions | Thromboembolisms in the CNS | Apoplexia trunci cerebri syndrom |
| G463A | Thromboembolic conditions | Thromboembolisms in the CNS | Benedikt's syndrom |
| G463B | Thromboembolic conditions | Thromboembolisms in the CNS | Claude's syndrom |
| G463C | Thromboembolic conditions | Thromboembolisms in the CNS | Foville's syndrom |
| G463D | Thromboembolic conditions | Thromboembolisms in the CNS | Millard-Gubler's syndrom |
| G463E | Thromboembolic conditions | Thromboembolisms in the CNS | Wallenberg's syndrom |
| G463F | Thromboembolic conditions | Thromboembolisms in the CNS | Weber's syndrom |
| G464 | Thromboembolic conditions | Thromboembolisms in the CNS | Apoplexia cerebellaris syndrom |
| G465 | Thromboembolic conditions | Thromboembolisms in the CNS | Lakunære syndromer m udelukkende motorisk udfald |
| G465A | Thromboembolic conditions | Thromboembolisms in the CNS | Hjernestammeapopleksi |
| G466 | Thromboembolic conditions | Thromboembolisms in the CNS | Lakunære syndromer m udelukkende sensorisk udfald |
| G467 | Thromboembolic conditions | Thromboembolisms in the CNS | Lakunære syndromer, andre |
| G468 | Other vascular conditions | Other vascular conditions in the CNS | Vaskulære syndromer ved cerebrovaskulær sygd, andre |
| G951A | Non-traumatic bleedings | Bleedings in the CNS | Haematomyelia |
| G951B | Thromboembolic conditions | Thromboembolisms in the CNS | Infarctus medullae spinalis acutus |
| G951F | Thromboembolic conditions | Thromboembolisms in the CNS | Thrombophlebitis intraspinalis, ikke pyogen |
| G951G | Thromboembolic conditions | Thromboembolisms in the CNS | Thrombosis arteriae medullae spinalis |
| H000A | Bacterial infections | Remaining infections | Abscessus palpebrae |
| H000B | Bacterial infections | Remaining infections | Furunculus palpebrae |
| H000D | Bacterial infections | Remaining infections | Phlegmone palpebrae |
| H01 | Bacterial infections | Remaining infections | Betændelse i øjelåg, anden |
| H010 | Bacterial infections | Remaining infections | Blepharitis |
| H018 | Bacterial infections | Remaining infections | Betændelse af øjelåg, anden specificeret |
| H019 | Bacterial infections | Remaining infections | Betændelse af øjelåg uden specifikation |
| H031E | Bacterial infections | Remaining infections | Tuberkulose i øjelåg |
| H038A | Bacterial infections | Remaining infections | Impetigo palpebrae |
| H040 | Bacterial infections | Remaining infections | Dacryoadenitis |
| H043 | Bacterial infections | Remaining infections | Dacryocystitis acuta og uden specifikation |
| H043A | Bacterial infections | Remaining infections | Canaliculitis lacrimalis acuta |
| H043B | Bacterial infections | Remaining infections | Canaliculitis lacrimalis uden specifikation |
| H043C | Bacterial infections | Remaining infections | Dacryocystitis acuta |
| H043D | Bacterial infections | Remaining infections | Dacryocystitis uden specifikation |
| H043E | Bacterial infections | Remaining infections | Dacryopericystitis acuta |
| H043F | Bacterial infections | Remaining infections | Dacryopericystitis uden specifikation |
| H044 | Bacterial infections | Remaining infections | Dacryocystitis chronica |
| H044A | Bacterial infections | Remaining infections | Canaliculitis lacrimalis chronica |
| H044B | Bacterial infections | Remaining infections | Dacryodochitis chronica |
| H050A | Bacterial infections | Remaining infections | Abscessus orbitae |
| H050B | Bacterial infections | Remaining infections | Cellulitis orbitae |
| H050C | Bacterial infections | Infections in joints and bones | Osteomyelitis orbitae |
| H050D | Bacterial infections | Infections in joints and bones | Periostitis orbitae |
| H050E | Bacterial infections | Remaining infections | Phlegmone orbitae |
| H050F | Bacterial infections | Remaining infections | Tenonitis orbitae |
| H052A | Non-traumatic bleedings | Remaining bleedings | Haemorrhagia orbitae |
| H10 | Bacterial infections | Remaining infections | Betændelse i øjets bindehinde |
| H100 | Bacterial infections | Remaining infections | Conjunctivitis mucopurulenta |
| H102 | Bacterial infections | Remaining infections | Conjunktivitis, anden akut |
| H103 | Bacterial infections | Remaining infections | Conjunctivitis acuta uden specifikation |
| H104 | Bacterial infections | Remaining infections | Conjunctivitis chronica |
| H105 | Bacterial infections | Remaining infections | Blepharoconjunctivitis |
| H108 | Bacterial infections | Remaining infections | Conjunktivitis, andre former |
| H109 | Bacterial infections | Remaining infections | Conjunktivitis uden specifikation |
| H113 | Non-traumatic bleedings | Remaining bleedings | Haemorrhagia conjunctivae |
| H113A | Non-traumatic bleedings | Remaining bleedings | Haemorrhagia subconjunctivae |
| H114A | Other vascular conditions | Remaining other vascular conditions | Aneurysma conjunctivae |
| H131 | Bacterial infections | Remaining infections | Conjunctivitis ved infekt og parasit sygdomme klass ansted |
| H131B | Bacterial infections | Remaining infections | Conjunctivitis haemorrhagica acuta |
| H131F | Bacterial infections | Remaining infections | Conjunctivitis ved infektiøs sygdom klassificeret andetsted |
| H131H | Bacterial infections | Remaining infections | Conjunctivitis meningococcica |
| H131I | Bacterial infections | Remaining infections | Conjunctivitis membranacea |
| H131L | Bacterial infections | Remaining infections | Conjunctivitis diphtheritica pseudomembranacea |
| H132 | Bacterial infections | Remaining infections | Conjunctivitis ved andre sygdomme klassificeret andetsteds |
| H16 | Bacterial infections | Remaining infections | Betændelse i hornhinde |
| H180A | Non-traumatic bleedings | Remaining bleedings | Haematocornea |
| H190C | Bacterial infections | Remaining infections | Episcleritis tuberculosa |
| H192 | Bacterial infections | Remaining infections | Keratitis et keratoconjunct ved infekt sygdomme klass anste |
| H192E | Bacterial infections | Remaining infections | Keratitis (interstitialis) tuberculosa |
| H192G | Bacterial infections | Remaining infections | Keratoconjunctivitis (interstitialis) tuberculosa |
| H192L | Bacterial infections | Remaining infections | Keratitis ved infektøs eller parasitær sygdom KA |
| H192M | Bacterial infections | Remaining infections | Keratokonjunktivitis ved infektøs eller parasitær sygdom KA |
| H200A | Bacterial infections | Remaining infections | Cyclitis subacuta |
| H200B | Bacterial infections | Remaining infections | Cyclitis recidivans |
| H200C | Bacterial infections | Remaining infections | Cyclitis acuta |
| H220B | Bacterial infections | Remaining infections | Iridocyclitis ved infektiøs sygdom klassificeret andetsteds |
| H220F | Bacterial infections | Remaining infections | Iridocyclitis tuberculosa |
| H313A | Non-traumatic bleedings | Remaining bleedings | Haemorrhagia chorioideae expulsiva |
| H313B | Non-traumatic bleedings | Remaining bleedings | Haemorrhagia chorioideae |
| H320 | Bacterial infections | Remaining infections | Chorioretinit ved infekt og parasit sygdomme klass ansted |
| H320A | Bacterial infections | Remaining infections | Chorioretinitis ved parasitær sygd klassificeret andetsteds |
| H320B | Bacterial infections | Remaining infections | Chorioretinitis ved infektiøs sygd klassificeret andetsteds |
| H320E | Bacterial infections | Remaining infections | Chorioretinitis tuberculosa |
| H340 | Thromboembolic conditions | Remaining thromboembolisms | Occlusio arteriae retinae transitoria |
| H341 | Thromboembolic conditions | Remaining thromboembolisms | Occlusio arteriae centralis retinae |
| H341A | Thromboembolic conditions | Remaining thromboembolisms | Embolia arteriae centralis retinae |
| H341B | Thromboembolic conditions | Remaining thromboembolisms | Thrombosis arteriae centralis retinae |
| H342 | Thromboembolic conditions | Remaining thromboembolisms | Okklusion af arteriae retinae, andre former |
| H342C | Thromboembolic conditions | Remaining thromboembolisms | Occlusio arteriae retinae partialis |
| H348A | Thromboembolic conditions | Remaining thromboembolisms | Occlusio venae retinae incipiens |
| H348B | Thromboembolic conditions | Remaining thromboembolisms | Occlusio venae retinae |
| H348C | Thromboembolic conditions | Remaining thromboembolisms | Occlusio venae retinae partialis |
| H348D | Thromboembolic conditions | Remaining thromboembolisms | Occlusio venae centralis retinae |
| H348E | Thromboembolic conditions | Remaining thromboembolisms | Thrombosis venae retinae |
| H348F | Thromboembolic conditions | Remaining thromboembolisms | Thrombosis venae centralis retinae |
| H349 | Thromboembolic conditions | Remaining thromboembolisms | Vaskulære okklusioner i retinae uden specifikation |
| H350C | Other vascular conditions | Remaining other vascular conditions | Microaneurysmus retinae |
| H356 | Non-traumatic bleedings | Remaining bleedings | Haemorrhagia retinae |
| H431 | Non-traumatic bleedings | Remaining bleedings | Haemorrhagia corporis vitrei |
| H440 | Non-traumatic bleedings | Remaining bleedings | Endophthalmitis purulenta |
| H440A | Bacterial infections | Remaining infections | Abscessus corporis vitrei |
| H440B | Bacterial infections | Remaining infections | Panoftalmitis |
| H441 | Bacterial infections | Remaining infections | Endophthalmitis, anden form |
| H441A | Bacterial infections | Remaining infections | Endophthalmitis parasitica uden specifikation |
| H441D | Bacterial infections | Remaining infections | Uveitis sympatica |
| H448B | Non-traumatic bleedings | Remaining bleedings | Haemophthalmos |
| H450 | Non-traumatic bleedings | Remaining bleedings | Haemorrhagia corporis vitrei ved sygdomme klass andetsteds |
| H451 | Bacterial infections | Remaining infections | Endophthalmit ved sygdomme klassificeret andetsteds |
| H46 | Bacterial infections | Remaining infections | Betændelse i synsnerve |
| H469C | Bacterial infections | Remaining infections | Papillitis retinae |
| H470B | Bacterial infections | Remaining infections | Haemorrhagia vaginae nervi optici |
| H60 | Bacterial infections | Remaining infections | Betændelse i ydre øre |
| H600 | Bacterial infections | Remaining infections | Abscessus auris ext |
| H600A | Bacterial infections | Remaining infections | Abscessus meatus acustici externi |
| H601 | Bacterial infections | Remaining infections | Phlegmone auris ext |
| H601A | Bacterial infections | Remaining infections | Cellulitis auris externae |
| H601B | Bacterial infections | Remaining infections | Cellulitis meatus acustici externi |
| H602 | Bacterial infections | Remaining infections | Otitis ext maligna |
| H603 | Bacterial infections | Remaining infections | Infektiøs otitis ext, anden form |
| H603A | Bacterial infections | Remaining infections | Badeotitis |
| H603B | Bacterial infections | Remaining infections | Otitis externa haemorrhagica |
| H603C | Bacterial infections | Remaining infections | Otitis externa diffusa |
| H605H | Bacterial infections | Remaining infections | Otitis externa acuta uden specifikation |
| H608 | Bacterial infections | Remaining infections | Otitis ext, anden form |
| H608A | Bacterial infections | Remaining infections | Otitis externa chronica uden specifikation |
| H609 | Bacterial infections | Remaining infections | Otitis ext uden specifikation |
| H610 | Bacterial infections | Remaining infections | Perichondritis auris ext |
| H610A | Bacterial infections | Remaining infections | Chondrodermatitis nodularis helicis |
| H610B | Bacterial infections | Remaining infections | Perichondritis pinnae |
| H620 | Bacterial infections | Remaining infections | Otitis ext ved bakteriel sygdom klassificeret andetsteds |
| H620A | Bacterial infections | Remaining infections | Otitis externa, erysipelas |
| H623 | Bacterial infections | Remaining infections | Otitis ext ved anden infek og parasit sygdom klass ansted |
| H623B | Bacterial infections | Remaining infections | Otitis externa ved andre infektiøs sygdom klass andetsteds |
| H624A | Bacterial infections | Remaining infections | Otitis externa, impetigo |
| H66 | Bacterial infections | Remaining infections | Mellemørebetændelse m pusdannelse og uden specifikation |
| H660 | Bacterial infections | Remaining infections | Otitis media purulenta acuta |
| H661 | Bacterial infections | Remaining infections | Otitis media purulenta chronica benigna |
| H661A | Bacterial infections | Remaining infections | Otitis media purulenta chronica tubotympanica |
| H662 | Bacterial infections | Remaining infections | Otitis media purulenta atticoantralis chronica |
| H663 | Bacterial infections | Remaining infections | Otitis media purulenta chronica, anden form |
| H664 | Bacterial infections | Remaining infections | Otitis media purulenta uden specifikation |
| H670 | Bacterial infections | Remaining infections | Otitis media ved bakteriel sygdom klassificeret andetsteds |
| H670B | Bacterial infections | Remaining infections | Otitis media tuberculosa |
| H671B | Bacterial infections | Remaining infections | Otitis media, influenza |
| H68 | Bacterial infections | Remaining infections | Betændelse og tillukning af det eustakiske rør |
| H680 | Bacterial infections | Remaining infections | Otosalpingitis |
| H70 | Bacterial infections | Remaining infections | Mastoiditis og beslægtede sygdom |
| H700 | Bacterial infections | Remaining infections | Mastoiditis acuta |
| H700A | Bacterial infections | Remaining infections | Abscessus processus mastoidei |
| H700B | Bacterial infections | Remaining infections | Empyema processus mastoidei |
| H701 | Bacterial infections | Remaining infections | Mastoiditis chronica |
| H702 | Bacterial infections | Remaining infections | Petrositis |
| H708 | Bacterial infections | Remaining infections | Mastoiditis og beslægtede sygdomme, andre former |
| H709 | Bacterial infections | Remaining infections | Mastoiditis uden specifikation |
| H750 | Bacterial infections | Remaining infections | Mastoiditis ved infek og parasit sygdom klass andetsteds |
| H750B | Bacterial infections | Remaining infections | Mastoiditis ved infektiøs sygdom klassificeret andetsteds |
| H750C | Bacterial infections | Remaining infections | Mastoiditis tuberculosa |
| H940 | Bacterial infections | Remaining infections | Neuritis n acustici ved infek og parasit sygdom klass anste |
| H940B | Bacterial infections | Remaining infections | Neuritis nervi acustici ved infektiøs sygd klass andetsteds |
| H951C | Bacterial infections | Remaining infections | Mastoiditis chronica post mastoidectomiam |
| I200 | Other vascular conditions | Remaining other vascular conditions | Angina pectoris (ustabil) |
| I200A | Other vascular conditions | Remaining other vascular conditions | Praeinfarkt syndrom |
| I200B | Other vascular conditions | Remaining other vascular conditions | Ustabil angina pectoris (alene klinisk vurderet) |
| I200C | Other vascular conditions | Remaining other vascular conditions | Ustabil angina pectoris (med dokumenteret iskæmi) |
| I208A | Other vascular conditions | Remaining other vascular conditions | Angina pectoris, anstrengelsesudløst |
| I208D | Other vascular conditions | Remaining other vascular conditions | Mikrovaskulær angina (Kardialt syndroma X) |
| I209 | Other vascular conditions | Remaining other vascular conditions | Angina pectoris uden specifikation |
| I21 | Thromboembolic conditions | Remaining thromboembolisms | Akut hjerteinfarkt |
| I210 | Thromboembolic conditions | Remaining thromboembolisms | Infarctus myocardii acutus transmuralis anterioris |
| I210A | Thromboembolic conditions | Remaining thromboembolisms | Non-ST-elevations AMI, anteriort med Q-taksudvikling |
| I210B | Thromboembolic conditions | Remaining thromboembolisms | ST-elevations AMI, anteriort med Q-taksudvikling |
| I211 | Thromboembolic conditions | Remaining thromboembolisms | Infarctus myocardii acutus transmuralis inferioris |
| I211A | Thromboembolic conditions | Remaining thromboembolisms | Non-ST-elevations AMI, inferiort/posteriort m. Q-taksudvikl |
| I211B | Thromboembolic conditions | Remaining thromboembolisms | ST-elevations AMI, inferiort/posteriort med Q-taksudvikling |
| I212 | Thromboembolic conditions | Remaining thromboembolisms | Infarctus myocardii acutus transmuralis m anden lokalisatio |
| I212A | Thromboembolic conditions | Remaining thromboembolisms | Infarctus myocardii acutus transmuralis posterolateralis |
| I212B | Thromboembolic conditions | Remaining thromboembolisms | Infarctus myocardii acutus transmuralis septalis |
| I212C | Thromboembolic conditions | Remaining thromboembolisms | Infarctus myocardii acutus transmuralis posterobasalis |
| I212D | Thromboembolic conditions | Remaining thromboembolisms | Infarctus myocardii acutus transmuralis posteroseptalis |
| I212E | Thromboembolic conditions | Remaining thromboembolisms | Infarctus myocardii acutus transmuralis apicolateralis |
| I212F | Thromboembolic conditions | Remaining thromboembolisms | Infarctus myocardii acutus transmuralis basolateralis |
| I212G | Thromboembolic conditions | Remaining thromboembolisms | Infarctus myocardii acutus transmuralis lateralis |
| I212H | Thromboembolic conditions | Remaining thromboembolisms | Infarctus myocardii acutus transmuralis posterioris |
| I213 | Thromboembolic conditions | Remaining thromboembolisms | Infarctus myocardii acutus transmuralis uden specifikation |
| I214 | Thromboembolic conditions | Remaining thromboembolisms | Infarctus myocardii acutus subendocardialis u spec |
| I219 | Thromboembolic conditions | Remaining thromboembolisms | Infarctus myocardii acutus uden specifikation |
| I22 | Thromboembolic conditions | Remaining thromboembolisms | Tilbagevendende akut hjerteinfarkt |
| I220 | Thromboembolic conditions | Remaining thromboembolisms | Infarctus myocardii acutus recidivans anterioris |
| I220A | Thromboembolic conditions | Remaining thromboembolisms | Infarctus myocardii acutus recidivans anteroseptalis |
| I220B | Thromboembolic conditions | Remaining thromboembolisms | Infarctus myocardii acutus recidivans anterolateralis |
| I220C | Thromboembolic conditions | Remaining thromboembolisms | Infarctus myocardii acutus recidivans anteroapicalis |
| I221 | Thromboembolic conditions | Remaining thromboembolisms | Infarctus myocardii acutus recidivans inferioris |
| I221A | Thromboembolic conditions | Remaining thromboembolisms | Infarctus myocardii acutus recidivans inferoposterioris |
| I221B | Thromboembolic conditions | Remaining thromboembolisms | Infarctus myocardii acutus recidivans inferolateralis |
| I221C | Thromboembolic conditions | Remaining thromboembolisms | Infarctus myocardii acutus recidivans diaphragmatica |
| I228 | Thromboembolic conditions | Remaining thromboembolisms | Infarctus myocardii acutus recidivans m anden lokalisation |
| I228A | Thromboembolic conditions | Remaining thromboembolisms | Infarctus myocardii acutus recidivans posteroseptalis |
| I228B | Thromboembolic conditions | Remaining thromboembolisms | Infarctus myocardii acutus recidivans posterolateralis |
| I228C | Thromboembolic conditions | Remaining thromboembolisms | Infarctus myocardii acutus recidivans septalis |
| I228D | Thromboembolic conditions | Remaining thromboembolisms | Infarctus myocardii acutus recidivans apicolateralis |
| I228E | Thromboembolic conditions | Remaining thromboembolisms | Infarctus myocardii acutus recidivans basolateralis |
| I228F | Thromboembolic conditions | Remaining thromboembolisms | Infarctus myocardii acutus recidivans lateralis |
| I228G | Thromboembolic conditions | Remaining thromboembolisms | Infarctus myocardii acutus recidivans posterioris |
| I228H | Thromboembolic conditions | Remaining thromboembolisms | Infarctus myocardii acutus recidivans posterobasalis |
| I229 | Thromboembolic conditions | Remaining thromboembolisms | Infarctus myocardii acutus recidivans uden specifikation |
| I230 | Non-traumatic bleedings | Remaining bleedings | Haemopericardium i efterforløbet af akut myokardieinfarkt |
| I236 | Thromboembolic conditions | Remaining thromboembolisms | Thromb atrii, ventriculi cordis efter akut myokardieinfarkt |
| I236A | Thromboembolic conditions | Remaining thromboembolisms | Thrombosis auriculae atrii cordis eft akut myokardieinfarkt |
| I236B | Thromboembolic conditions | Remaining thromboembolisms | Thrombosis ventriculi cordis efter akut myokardieinfarkt |
| I24 | Other vascular conditions | Remaining other vascular conditions | Akut iskæmisk hjertesygdom, anden form |
| I240 | Thromboembolic conditions | Remaining thromboembolisms | Thrombosis coronariae (arteriel eller venøs) u infarcering |
| I240A | Thromboembolic conditions | Remaining thromboembolisms | Embolia coronariae (arteriae, venae) uden infarcering |
| I248 | Other vascular conditions | Remaining other vascular conditions | Akut iskæmisk hjertesygdom, andre former |
| I248A | Other vascular conditions | Remaining other vascular conditions | Insufficientia coronaria |
| I249 | Other vascular conditions | Remaining other vascular conditions | Akut iskæmisk hjertesygdom uden specifikation |
| I25 | Other vascular conditions | Remaining other vascular conditions | Kronisk iskæmisk hjertesygdom |
| I250 | Other vascular conditions | Remaining other vascular conditions | Arteriosclerosis cardiovascularis |
| I251 | Other vascular conditions | Remaining other vascular conditions | Morbus cordis arterioscleroticus |
| I251A | Other vascular conditions | Remaining other vascular conditions | Ateriosclerosis arteriae coronariae |
| I251B | Other vascular conditions | Remaining other vascular conditions | Angina pectoris (alene klinisk vurderet) |
| I251C | Other vascular conditions | Remaining other vascular conditions | Angina pectoris (med dokumenteret iskæmi) |
| I252 | Thromboembolic conditions | Remaining thromboembolisms | Infarctus myocardii antea |
| I253 | Other vascular conditions | Remaining other vascular conditions | Aneurysma cordis |
| I254 | Other vascular conditions | Remaining other vascular conditions | Aneurysma arteriae coronariae |
| I254A | Other vascular conditions | Remaining other vascular conditions | Fistula arteriovenosa coronaria acquisita |
| I255 | Other vascular conditions | Remaining other vascular conditions | Cardiomyopathia ischaemica |
| I256 | Other vascular conditions | Remaining other vascular conditions | Ischaemia myocardii asymptomatica |
| I256A | Other vascular conditions | Remaining other vascular conditions | Søvnrelateret iskæmisk hjertesygdom |
| I258 | Other vascular conditions | Remaining other vascular conditions | Kronisk iskæmisk hjertesygdom, andre former |
| I259 | Other vascular conditions | Remaining other vascular conditions | Kronisk iskæmisk hjertesygdom uden specifikation |
| I26 | Thromboembolic conditions | Remaining thromboembolisms | Blodprop i lunge |
| I260 | Thromboembolic conditions | Remaining thromboembolisms | Embolia pulmonis m akut cor pulmonale |
| I260A | Thromboembolic conditions | Remaining thromboembolisms | Cor pulmonale acutum uden specifikation |
| I269 | Thromboembolic conditions | Remaining thromboembolisms | Embolia pulmonis u akut cor pulmonale |
| I269A | Thromboembolic conditions | Remaining thromboembolisms | Embolia pulmonis uden specifikation |
| I272 | Thromboembolic conditions | Remaining thromboembolisms | Kronisk tromboembolisk pulmonal hypertension |
| I280 | PAVMs | PAVMs | Fistula arteriovenosa pulmonalis |
| I281 | PAVMs | PAVMs | Aneurysma arteriae pulmonalis |
| I30 | Bacterial infections | Remaining infections | Akut betændelse i hjertesækken |
| I300 | Bacterial infections | Remaining infections | Pericarditis acuta idiopathica |
| I301 | Bacterial infections | Remaining infections | Pericarditis infectiosa |
| I301A | Bacterial infections | Remaining infections | Pericarditis, pneumokok |
| I301B | Bacterial infections | Remaining infections | Pericarditis purulenta |
| I301C | Bacterial infections | Remaining infections | Pericarditis, staphylokok |
| I301D | Bacterial infections | Remaining infections | Pericarditis, streptokok |
| I310 | Bacterial infections | Remaining infections | Pericarditis adhaesiva chronica |
| I312 | Non-traumatic bleedings | Remaining bleedings | Haemopericardium ikke klassificeret andetsteds |
| I320 | Bacterial infections | Remaining infections | Perikardit ved bakterielle sygdom klassificeret andetsteds |
| I320B | Bacterial infections | Remaining infections | Pericarditis meningococcica |
| I320D | Bacterial infections | Remaining infections | Pericarditis tuberculosa |
| I321 | Bacterial infections | Remaining infections | Perikardit ved anden infekti og parasit sygdom klass ansted |
| I321B | Bacterial infections | Remaining infections | Pericarditis ved infektiøs sygdom klassificeret andetsteds |
| I330 | Bacterial infections | Remaining infections | Endocarditis infectiosa acuta |
| I330A | Bacterial infections | Remaining infections | Endocarditis infectiosa subacuta |
| I330B | Bacterial infections | Remaining infections | Endocarditis infectiosa chronica |
| I330C | Bacterial infections | Remaining infections | Endocarditis lenta |
| I330D | Bacterial infections | Remaining infections | Endocarditis ulcerativa acuta |
| I330E | Bacterial infections | Remaining infections | Endocarditis ulcerativa subacuta |
| I330F | Bacterial infections | Remaining infections | Endocarditis ulcerativa chronica |
| I339 | Bacterial infections | Remaining infections | Endocarditis acuta uden specifikation |
| I35 | Other vascular conditions | Remaining other vascular conditions | Ikke reumatiske sygdom i aortaklapper |
| I350 | Other vascular conditions | Remaining other vascular conditions | Stenosis aortae non rheumatica |
| I351 | Other vascular conditions | Remaining other vascular conditions | Insufficientia aortae non rheumatica |
| I352 | Other vascular conditions | Remaining other vascular conditions | Stenosis et insufficientia aortae non rheumatica |
| I358 | Other vascular conditions | Remaining other vascular conditions | Ikke reumatisk aortaklapsygdom, andre former |
| I358A | Other vascular conditions | Remaining other vascular conditions | Aortaklapsklerose |
| I359 | Other vascular conditions | Remaining other vascular conditions | Ikke reumatiske aortaklapsygdom uden specifikation |
| I389 | Bacterial infections | Remaining infections | Endokardit uden specifikation |
| I39 | Bacterial infections | Remaining infections | Betændelse m.m. af hjerteklap ved sygdom klass andetsteds |
| I398 | Bacterial infections | Remaining infections | Endokardit u spec ved sygdom klassificeret andetsteds |
| I398D | Bacterial infections | Remaining infections | Endocarditis meningococcica |
| I398G | Bacterial infections | Remaining infections | Endocarditis tuberculosa |
| I398H | Bacterial infections | Remaining infections | Endocarditis typhoidea |
| I40 | Bacterial infections | Remaining infections | Akut betændelse i hjertemuskulaturen |
| I400 | Bacterial infections | Remaining infections | Myocarditis infectiosa |
| I400A | Bacterial infections | Sepsis | Myocarditis septica |
| I401 | Bacterial infections | Remaining infections | Myocarditis monosymptomatica |
| I408 | Bacterial infections | Remaining infections | Myocarditis acuta, anden form |
| I41 | Bacterial infections | Remaining infections | Betændelse i hjertemuskulaturen ved sygdom klass andetsteds |
| I410 | Bacterial infections | Remaining infections | Myokardit ved bakterielle sygdom klassificeret andetsteds |
| I410A | Bacterial infections | Remaining infections | Myocarditis diphtheritica |
| I410C | Bacterial infections | Remaining infections | Myocarditis meningococcica |
| I410E | Bacterial infections | Remaining infections | Myocarditis tuberculosa |
| I518A | Bacterial infections | Remaining infections | Carditis acuta |
| I518B | Bacterial infections | Remaining infections | Carditis chronica |
| I60 | Non-traumatic bleedings | Remaining bleedings | Hjernehindeblødning |
| I600 | Non-traumatic bleedings | Bleedings in the CNS | Haemorrhagia subarachnoidalis (carotissiphonen) |
| I601 | Non-traumatic bleedings | Bleedings in the CNS | Haemorrhagia subarachnoidalis (arteria cerebri media) |
| I602 | Non-traumatic bleedings | Bleedings in the CNS | Haemorrhagia subarachnoidalis (arteria communicans anterior |
| I603 | Non-traumatic bleedings | Bleedings in the CNS | Haemorrhagia subarachnoidalis (arteria communicans post.) |
| I604 | Non-traumatic bleedings | Bleedings in the CNS | Haemorrhagia subarachnoidalis (arteria basilaris) |
| I605 | Non-traumatic bleedings | Bleedings in the CNS | Haemorrhagia subarachnoidalis (arteria vertebralis) |
| I606 | Non-traumatic bleedings | Bleedings in the CNS | Haemorrhagia subarachnoidalis (andre intrakran. arterier) |
| I606A | Non-traumatic bleedings | Bleedings in the CNS | Haemorrhagia subarachnoidalis, arteria cerebri posterior |
| I606B | Non-traumatic bleedings | Bleedings in the CNS | Haemorrhagia subarachnoidalis, arteria cerebri anterior |
| I606C | Non-traumatic bleedings | Bleedings in the CNS | Haemorrhagia subarachnoidalis, arteriae intracran multiplex |
| I606D | Non-traumatic bleedings | Bleedings in the CNS | Haemorrhagia subarachnoidalis, anden arterie |
| I607 | Non-traumatic bleedings | Bleedings in the CNS | Haemorrhagia subarachnoidalis (intrakraniel arterie u spec) |
| I607A | Non-traumatic bleedings | Bleedings in the CNS | Aneurysma intracraniale sacculatum congenitum ruptum |
| I608 | Non-traumatic bleedings | Bleedings in the CNS | haemorrhagia subarachnoidalis, anden form |
| I609 | Non-traumatic bleedings | Bleedings in the CNS | Haemorrhagia subarachnoidalis uden specifikation |
| I609A | Non-traumatic bleedings | Bleedings in the CNS | Bristet (medfødt) intrakranielt aneurisme UNS |
| I61 | Non-traumatic bleedings | Bleedings in the CNS | Hjerneblødning |
| I610 | Non-traumatic bleedings | Bleedings in the CNS | Haemorrhagia cerebri hemisphaeri subcorticalis |
| I610A | Non-traumatic bleedings | Bleedings in the CNS | Haemorrhagia cerebri profunda |
| I611 | Non-traumatic bleedings | Bleedings in the CNS | Haemorrhagia cerebri hemisphaeri corticalis |
| I611A | Non-traumatic bleedings | Bleedings in the CNS | Haemorrhagia cerebri superficialis |
| I611B | Non-traumatic bleedings | Bleedings in the CNS | Haemorrhagia lobi cerebri |
| I612 | Non-traumatic bleedings | Bleedings in the CNS | Haemorrhagia cerebri hemisphaeri uden specifikation |
| I613 | Non-traumatic bleedings | Bleedings in the CNS | Haemorrhagia cerebri (hjernestamme) |
| I614 | Non-traumatic bleedings | Bleedings in the CNS | Haemorrhagia cerebri (cerebellum) |
| I615 | Non-traumatic bleedings | Bleedings in the CNS | Haemorrhagia cerebri intraventricularis |
| I616 | Non-traumatic bleedings | Bleedings in the CNS | Haemorrhagia cerebri (multiple lokalisationer) |
| I618 | Non-traumatic bleedings | Bleedings in the CNS | Haemorrhagia cerebri, anden form |
| I619 | Non-traumatic bleedings | Bleedings in the CNS | Haemorrhagia cerebri uden specifikation |
| I62 | Non-traumatic bleedings | Bleedings in the CNS | Ikke traumatisk intrakraniel blødning, anden |
| I620 | Non-traumatic bleedings | Bleedings in the CNS | Haemorrhagia subduralis (acuta, non traumatica) |
| I621 | Non-traumatic bleedings | Bleedings in the CNS | Haemorrhagia epiduralis non traumatica |
| I629 | Non-traumatic bleedings | Bleedings in the CNS | Haemorrhagia epiduralis non traumatica uden specifikation |
| I63 | Thromboembolic conditions | Thromboembolisms in the CNS | Infarctus cerebri |
| I630 | Thromboembolic conditions | Thromboembolisms in the CNS | Infarctus cerebri, thrombosis arteriae praecerebralis |
| I631 | Thromboembolic conditions | Thromboembolisms in the CNS | Infarctus cerebri, embolia arteriae praecerebralis |
| I632 | Thromboembolic conditions | Thromboembolisms in the CNS | Infarctus cerebri, occl sive sten art praecerebralis u spec |
| I633 | Thromboembolic conditions | Thromboembolisms in the CNS | Infarctus cerebri, thrombosis arteriae cerebri |
| I634 | Thromboembolic conditions | Thromboembolisms in the CNS | Infarctus cerebri, embolia arteriae cerebri |
| I634A | Thromboembolic conditions | Thromboembolisms in the CNS | Embolia cerebri |
| I635 | Thromboembolic conditions | Thromboembolisms in the CNS | Infarctus cerebri, occl sive sten art cerebri u spec |
| I636 | Thromboembolic conditions | Thromboembolisms in the CNS | Infarctus cerebri, thrombosis venae cerebri non pyogenica |
| I638 | Thromboembolic conditions | Thromboembolisms in the CNS | Infarctus cerebri, anden form |
| I639 | Thromboembolic conditions | Thromboembolisms in the CNS | Infarctus cerebri uden specifikation |
| I64 | Thromboembolic conditions | Thromboembolisms in the CNS | Slagtilfælde u oplysning om blødning eller infarkt |
| I649 | Thromboembolic conditions | Thromboembolisms in the CNS | Apoplexia cerebri uden specifikation |
| I65 | Other vascular conditions | Other vascular conditions in the CNS | Tillukning og forsnævring af hjernens kar u hjerneinfarkt |
| I650 | Other vascular conditions | Other vascular conditions in the CNS | Occlusio et stenosis arteriae vertebralis u hjerneinfarkt |
| I650A | Other vascular conditions | Other vascular conditions in the CNS | Occlusio arteriae vertebralis uden hjerneinfarkt |
| I650B | Other vascular conditions | Other vascular conditions in the CNS | Stenosis arteriae vertebralis uden hjerneinfarkt |
| I651 | Other vascular conditions | Other vascular conditions in the CNS | Occlusio et stenosis arteriae basilaris u hjerneinfarkt |
| I651A | Other vascular conditions | Other vascular conditions in the CNS | Occlusio arteriae basilaris uden hjerneinfarkt |
| I651B | Other vascular conditions | Other vascular conditions in the CNS | Stenosis arteriae basilaris uden hjerneinfarkt |
| I652 | Other vascular conditions | Other vascular conditions in the CNS | Occlusio et stenosis arteriae carotidis u hjerneinfarkt |
| I652A | Other vascular conditions | Other vascular conditions in the CNS | Occlusio arteriae carotis uden hjerneinfarkt |
| I652B | Other vascular conditions | Other vascular conditions in the CNS | Stenosis arteriae carotis uden hjerneinfarkt |
| I653 | Other vascular conditions | Other vascular conditions in the CNS | Occl et sten art praecereb multip et bilat u hjerneinfarkt |
| I653A | Other vascular conditions | Other vascular conditions in the CNS | Occlusio arteriae praecerebralis bilat uden hjerneinfarkt |
| I653B | Other vascular conditions | Other vascular conditions in the CNS | Occlusio arteriae praecerebralis multiplex u hjerneinfarkt |
| I653C | Other vascular conditions | Other vascular conditions in the CNS | Stenosis arteriae praecerebralis multiplex u hjerneinfarkt |
| I653D | Other vascular conditions | Other vascular conditions in the CNS | Stenosis arteriae praecerebralis bilateralis u hjerneinfark |
| I658 | Other vascular conditions | Other vascular conditions in the CNS | Tillukning og forsnævring af hjernens kar u hjerneinfarkt |
| I659 | Other vascular conditions | Other vascular conditions in the CNS | Tillukning og forsnævr af hjernens kar u hjerneinf, u spec |
| I66 | Other vascular conditions | Other vascular conditions in the CNS | Tillukning og forsnævring af hjernekar u infarkt |
| I660 | Other vascular conditions | Other vascular conditions in the CNS | Occlusio et stenosis arteriae cerebri u hjerneinfarkt |
| I660A | Other vascular conditions | Other vascular conditions in the CNS | Occlusio arteriae cerebri mediae uden hjerneinfarkt |
| I660B | Other vascular conditions | Other vascular conditions in the CNS | Stenosis arteriae cerebri mediae uden hjerneinfarkt |
| I661 | Other vascular conditions | Other vascular conditions in the CNS | Occlusio et stenosis arteriae cerebri ant u hjerneinfarkt |
| I661A | Other vascular conditions | Other vascular conditions in the CNS | Occlusio arteriae cerebri anterioris uden hjerneinfarkt |
| I661B | Other vascular conditions | Other vascular conditions in the CNS | Stenosis arteriae cerebri anterioris uden hjerneinfarkt |
| I662 | Other vascular conditions | Other vascular conditions in the CNS | Occlusio et stenosis arteriae cerebri post u hjerneinfarkt |
| I662A | Other vascular conditions | Other vascular conditions in the CNS | Occlusio arteriae cerebri posterioris uden hjerneinfarkt |
| I662B | Other vascular conditions | Other vascular conditions in the CNS | Stenosis arteriae cerebri posterioris uden hjerneinfarkt |
| I663 | Other vascular conditions | Other vascular conditions in the CNS | Occlusio et stenosis arteriae cerebelli u hjerneinfarkt |
| I663A | Other vascular conditions | Other vascular conditions in the CNS | Occlusio arteriae cerebelli uden hjerneinfarkt |
| I663B | Other vascular conditions | Other vascular conditions in the CNS | Stenosis arteriae cerebelli uden hjerneinfarkt |
| I664 | Other vascular conditions | Other vascular conditions in the CNS | Occl et sten art cerebri multiplex et bilat u hjerneinfarkt |
| I664A | Other vascular conditions | Other vascular conditions in the CNS | Occlusio arteriae cerebri bilateralis uden hjerneinfarkt |
| I664B | Other vascular conditions | Other vascular conditions in the CNS | Occlusio arteriae cerebri multiplex uden hjerneinfarkt |
| I664C | Other vascular conditions | Other vascular conditions in the CNS | Stenosis arteriae cerebri bilateralis uden hjerneinfarkt |
| I664D | Other vascular conditions | Other vascular conditions in the CNS | Stenosis arteriae cerebri multiplex uden hjerneinfarkt |
| I668 | Other vascular conditions | Other vascular conditions in the CNS | Tillukning og forsnævring af hjernekar u hjerneinfarkt |
| I668A | Other vascular conditions | Other vascular conditions in the CNS | Occlusio arteriae perforantis cerebri uden hjerneinfarkt |
| I669 | Other vascular conditions | Other vascular conditions in the CNS | Tillukn og forsnævr af hjernekar u hjerneinfarkt og u spec |
| I671 | Other vascular conditions | Other vascular conditions in the CNS | Aneurysma cerebri non ruptum |
| I671A | Other vascular conditions | Other vascular conditions in the CNS | Fistula arteriovenosa cerebri acquisita |
| I672 | Other vascular conditions | Other vascular conditions in the CNS | Arteriosclerosis cerebri |
| I673A | Other vascular conditions | Other vascular conditions in the CNS | Binswanger's sygdom |
| I676 | Thromboembolic conditions | Thromboembolisms in the CNS | Thrombosis venae intracranialis non pyogenica |
| I676A | Thromboembolic conditions | Thromboembolisms in the CNS | Thrombosis sinuum durae matris non pyogenica |
| I678 | Other vascular conditions | Other vascular conditions in the CNS | Cerebrovaskulær sygdom, andre spec former |
| I678A | Other vascular conditions | Other vascular conditions in the CNS | Insufficientia cerebrovascularis acuta |
| I678B | Other vascular conditions | Other vascular conditions in the CNS | Ischaemia cerebri |
| I679 | Other vascular conditions | Other vascular conditions in the CNS | Cerebrovaskulær sygdom uden specifikation |
| I68 | Other vascular conditions | Other vascular conditions in the CNS | Karsygdom i hjerne ved sygdom klassificeret andetsteds |
| I681 | Other vascular conditions | Other vascular conditions in the CNS | Arteritis cerebri ved infektiøse og parasitære sygdom |
| I681A | Other vascular conditions | Other vascular conditions in the CNS | Arteritis cerebri ved infektiøs sygdom |
| I681C | Other vascular conditions | Other vascular conditions in the CNS | Arteritis cerebri tuberculosa |
| I681D | Other vascular conditions | Other vascular conditions in the CNS | Arteritis cerebri listerica |
| I688 | Other vascular conditions | Other vascular conditions in the CNS | Cerebrovaskulære, anden sygdom ved sygdom klass andetsteds |
| I69 | Other vascular conditions | Other vascular conditions in the CNS | Følger efter karsygdom i hjerne |
| I690 | Non-traumatic bleedings | Bleedings in the CNS | Sequelae haemorrhagiae subarachnoidalis |
| I691 | Non-traumatic bleedings | Bleedings in the CNS | Sequelae haemorrhagiae cerebri |
| I692 | Non-traumatic bleedings | Bleedings in the CNS | Sequelae efter haemorrhagia intracran non traum, amdem form |
| I693 | Thromboembolic conditions | Thromboembolisms in the CNS | Sequelae infarcti cerebri |
| I694 | Thromboembolic conditions | Thromboembolisms in the CNS | Sequelae apoplexiae cerebri |
| I698 | Other vascular conditions | Other vascular conditions in the CNS | Sequelae efter anden cerebrovaskulær sygdom og u spec |
| I70 | Other vascular conditions | Remaining other vascular conditions | Åreforkalkning |
| I700 | Other vascular conditions | Remaining other vascular conditions | Arteriosclerosis aortae |
| I701 | Other vascular conditions | Remaining other vascular conditions | Arteriosclerosis arteriae renalis |
| I702 | Other vascular conditions | Remaining other vascular conditions | Arteriosclerosis arteriae extremitatis inferioris |
| I702A | Other vascular conditions | Remaining other vascular conditions | Gangraena atherosclerotica |
| I702B | Other vascular conditions | Remaining other vascular conditions | Mönckeberg's mediasclerose |
| I708 | Other vascular conditions | Remaining other vascular conditions | Arteriosclerose i andre arterier |
| I708A | Other vascular conditions | Remaining other vascular conditions | Aterosklerotisk retinopati |
| I709 | Other vascular conditions | Remaining other vascular conditions | Arteriosclerosis uden specifikation |
| I71 | Other vascular conditions | Remaining other vascular conditions | Aortaaneurisme og dissekerende aortaaneurisme |
| I710 | Other vascular conditions | Remaining other vascular conditions | Aneurysma aortae dissecans, alle lokalisationer |
| I710A | Other vascular conditions | Remaining other vascular conditions | Aortadissektion, Type A |
| I710B | Other vascular conditions | Remaining other vascular conditions | Aortadissektion, Type B |
| I711 | Other vascular conditions | Remaining other vascular conditions | Aneurysma aortae thoracalis ruptum |
| I712 | Other vascular conditions | Remaining other vascular conditions | Aneurysma aortae thoracalis non ruptum |
| I713 | Other vascular conditions | Remaining other vascular conditions | Aneurysma aortae abdominalis ruptum |
| I714 | Other vascular conditions | Remaining other vascular conditions | Aneurysma aortae abdominalis non ruptum |
| I715 | Other vascular conditions | Remaining other vascular conditions | Aneurysma aortae thoracoabdominalis ruptum |
| I716 | Other vascular conditions | Remaining other vascular conditions | Aneurysma aortae thoracoabdominalis non ruptum |
| I718 | Other vascular conditions | Remaining other vascular conditions | Bristet aortaaneurysme u lokalisation |
| I719 | Other vascular conditions | Remaining other vascular conditions | Aortaaneurysma u lokalisation |
| I719A | Other vascular conditions | Remaining other vascular conditions | Dilatatio aortae |
| I72 | Other vascular conditions | Remaining other vascular conditions | Aneurismer, andre |
| I720 | Other vascular conditions | Remaining other vascular conditions | Aneurysma arteriae carotidis |
| I721 | Other vascular conditions | Remaining other vascular conditions | Aneurysma arteriae extremitatis superioris |
| I722 | Other vascular conditions | Remaining other vascular conditions | Aneurysma arteriae renalis |
| I723 | Other vascular conditions | Remaining other vascular conditions | Aneurysma arteriae iliacae |
| I724 | Other vascular conditions | Remaining other vascular conditions | Aneurysma arteriae extremitatis inferioris |
| I725 | Other vascular conditions | Remaining other vascular conditions | Aneurisme i anden præcerebral arterie |
| I726 | Other vascular conditions | Remaining other vascular conditions | Aneurisme på arteria vertebralis |
| I728 | Other vascular conditions | Remaining other vascular conditions | Aneurysme på andre specificerede arterier |
| I729 | Other vascular conditions | Remaining other vascular conditions | Aneurysme u lokalisation |
| I739A | Other vascular conditions | Remaining other vascular conditions | Claudicatio intermittens |
| I739C | Other vascular conditions | Remaining other vascular conditions | Iskæmiske hvilesmerter i underekstremitet |
| I74 | Thromboembolic conditions | Remaining thromboembolisms | Blodprop i pulsårer |
| I740 | Thromboembolic conditions | Remaining thromboembolisms | Embolia et thrombosis aortae abdominalis |
| I740A | Thromboembolic conditions | Remaining thromboembolisms | Bifurcatura aortae syndrom |
| I740B | Thromboembolic conditions | Remaining thromboembolisms | Embolia aortae abdominalis |
| I740C | Thromboembolic conditions | Remaining thromboembolisms | Leriche's syndrom |
| I740D | Thromboembolic conditions | Remaining thromboembolisms | Thrombosis aortae abdominalis |
| I741 | Thromboembolic conditions | Remaining thromboembolisms | Embolia et thromb aortae m anden og u lokalisation |
| I741A | Thromboembolic conditions | Remaining thromboembolisms | Embolia aortae uden lokalisation |
| I741B | Thromboembolic conditions | Remaining thromboembolisms | Thrombosis aortae uden lokalisation |
| I742 | Thromboembolic conditions | Remaining thromboembolisms | Embolia et thrombosis arteriae extremitatis superioris |
| I742A | Thromboembolic conditions | Remaining thromboembolisms | Embolia arteriae extremitatis superioris |
| I742B | Thromboembolic conditions | Remaining thromboembolisms | Thrombosis arteriae extremitatis superioris |
| I743 | Thromboembolic conditions | Remaining thromboembolisms | Embolia et thrombosis arteriae extremitatis inferioris |
| I743A | Thromboembolic conditions | Remaining thromboembolisms | Embolia arteriae extremitatis inferioris |
| I743B | Thromboembolic conditions | Remaining thromboembolisms | Thrombosis arteriae extremitatis inferioris |
| I744 | Thromboembolic conditions | Remaining thromboembolisms | Embolia et thrombosis arteriae extremitatis u specifikation |
| I744A | Thromboembolic conditions | Remaining thromboembolisms | Embolia arteriae extremitatis uden specifikation |
| I744B | Thromboembolic conditions | Remaining thromboembolisms | Thrombosis arteriae extremitatis uden specifikation |
| I744C | Thromboembolic conditions | Remaining thromboembolisms | Embolia arteriae periphericae |
| I744D | Thromboembolic conditions | Remaining thromboembolisms | Livedo reticularis (kolesterolembolier) |
| I744E | Thromboembolic conditions | Remaining thromboembolisms | Kolesterol-emboli |
| I745 | Thromboembolic conditions | Remaining thromboembolisms | Embolia et thrombosis arteriae iliacae |
| I745A | Thromboembolic conditions | Remaining thromboembolisms | Embolia arteriae iliacae |
| I745B | Thromboembolic conditions | Remaining thromboembolisms | Thrombosis arteriae iliacae |
| I748 | Thromboembolic conditions | Remaining thromboembolisms | Emboli og thrombose i andre arterier |
| I749 | Thromboembolic conditions | Remaining thromboembolisms | Emboli og thrombose i arterie uden specifikation |
| I77 | Other vascular conditions | Remaining other vascular conditions | Sygdom i arterier og arterioler, anden |
| I770 | Other vascular conditions | Remaining other vascular conditions | Fistula arteriovenosa acquisita |
| I770A | Other vascular conditions | Remaining other vascular conditions | Aneurysma varicosum |
| I772B | Other vascular conditions | Remaining other vascular conditions | Fistula arteriae |
| I778 | Other vascular conditions | Remaining other vascular conditions | Sygdom i arterier og arterioler, anden specificeret |
| I779 | Other vascular conditions | Remaining other vascular conditions | Sygdom i arterier og arterioler uden specifikation |
| I78 | Other vascular conditions | Remaining other vascular conditions | Sygdom i kapillæer |
| I780 | HHT as diagnosis | HHT as diagnosis | Telangiectasia haemorrhagica hereditaria |
| I780A | HHT as diagnosis | HHT as diagnosis | Osler-Weber's sygdom |
| I780B | Other vascular conditions | Remaining other vascular conditions | Telangiectasia generalisata, essentiel |
| I788 | Other vascular conditions | Remaining other vascular conditions | Sygdom i kapillæer, anden |
| I788A | Other vascular conditions | Remaining other vascular conditions | Telangiectasia, secundaria |
| I788B | Other vascular conditions | Remaining other vascular conditions | Telangiectasia uden specifikation |
| I788C | Other vascular conditions | Remaining other vascular conditions | Telangiectasia actinica |
| I788D | Other vascular conditions | Remaining other vascular conditions | Telangiectasia cosmetica |
| I789 | Other vascular conditions | Remaining other vascular conditions | Kapillærsygdom uden specifikation |
| I790 | Other vascular conditions | Remaining other vascular conditions | Aneurysma aortae ved sygdom klassificeret andetsteds |
| I791 | Bacterial infections | Remaining infections | Aortitis ved sygdom klassificeret andetsteds |
| I80 | Other vascular conditions | Remaining other vascular conditions | Årebetændelse |
| I800 | Thromboembolic conditions | Remaining thromboembolisms | Phlebitis et thrombophlebitis superficialis extremitatis in |
| I800B | Thromboembolic conditions | Remaining thromboembolisms | Thrombophlebitis superficialis extremitatis inferioris |
| I801 | Thromboembolic conditions | Remaining thromboembolisms | Phlebitis et thrombophlebitis venae femoralis |
| I801B | Thromboembolic conditions | Remaining thromboembolisms | Thrombophlebitis venae femoralis |
| I802 | Thromboembolic conditions | Remaining thromboembolisms | Phlebitis et thrombophleb prof i andre kar på underextr |
| I802B | Thromboembolic conditions | Remaining thromboembolisms | Thrombosis profunda extremitatis inferioris uden specifikat |
| I803 | Thromboembolic conditions | Remaining thromboembolisms | Phlebitis et thrombophlebitis profunda extr inf u spec |
| I803A | Thromboembolic conditions | Remaining thromboembolisms | Embolia extremitatis inferioris uden specifikation |
| I803D | Thromboembolic conditions | Remaining thromboembolisms | Thrombophlebitis extremitatis inferioris uden specifikation |
| I803E | Thromboembolic conditions | Remaining thromboembolisms | Thrombophlebitis profunda extremitatis inferioris uden spec |
| I803F | Thromboembolic conditions | Remaining thromboembolisms | Thrombosis extremitatis inferioris uden specifikation |
| I808 | Thromboembolic conditions | Remaining thromboembolisms | Phlebitis et thrombophlebitis m anden lokalisation |
| I808B | Thromboembolic conditions | Remaining thromboembolisms | Thrombophlebitis med anden lokalisation |
| I808D | Thromboembolic conditions | Remaining thromboembolisms | Phlebitis et thrombophlebitis venae jugularis |
| I809 | Thromboembolic conditions | Remaining thromboembolisms | Phlebitis et thrombophlebitis u lokalisation |
| I809B | Thromboembolic conditions | Remaining thromboembolisms | Thrombophlebitis uden lokalisation |
| I81 | Thromboembolic conditions | Remaining thromboembolisms | Blodprop i portåre |
| I819 | Thromboembolic conditions | Remaining thromboembolisms | Thrombosis venae portae |
| I82 | Thromboembolic conditions | Remaining thromboembolisms | Blodprop i blodåre, anden |
| I820 | Thromboembolic conditions | Remaining thromboembolisms | Budd-Chiari syndrom |
| I821 | Thromboembolic conditions | Remaining thromboembolisms | Thrombophlebitis migrans |
| I822 | Thromboembolic conditions | Remaining thromboembolisms | Embolia et thrombosis venae cavae |
| I822A | Thromboembolic conditions | Remaining thromboembolisms | Embolia venae cavae |
| I822B | Thromboembolic conditions | Remaining thromboembolisms | Thrombosis venae cavae |
| I823 | Thromboembolic conditions | Remaining thromboembolisms | Embolia et thrombosis venae renalis |
| I823A | Thromboembolic conditions | Remaining thromboembolisms | Embolia venae renalis |
| I823B | Thromboembolic conditions | Remaining thromboembolisms | Thrombosis venae renalis |
| I828 | Thromboembolic conditions | Remaining thromboembolisms | Emboli og trombose i andre specificerede vener |
| I829 | Thromboembolic conditions | Remaining thromboembolisms | Emboli og trombose i vene uden specifikation |
| I829A | Thromboembolic conditions | Remaining thromboembolisms | Embolia i vene uden specifikation |
| I829B | Thromboembolic conditions | Remaining thromboembolisms | Thrombosis i vene uden specifikation |
| I841C | Non-traumatic bleedings | Remaining bleedings | Tumores haemorrhoidales interni med blødning |
| I844C | Non-traumatic bleedings | Remaining bleedings | Tumores haemorrhoidales externi med blødning |
| I850 | Non-traumatic bleedings | Bleedings in the GI tract | Varices oesophagi m blødning |
| I864A | Non-traumatic bleedings | Bleedings in the GI tract | Varices gastricae med blødning |
| I979A | Other vascular conditions | Remaining other vascular conditions | Iskæmisk sår på underekstremitet |
| I979E | Other vascular conditions | Remaining other vascular conditions | Iskæmisk sår på underekstremitet |
| J009 | Bacterial infections | Remaining infections | Nasopharyngitis acuta |
| J009A | Bacterial infections | Remaining infections | Rhinitis infectiosa |
| J009B | Bacterial infections | Remaining infections | Catarrhalia acuta |
| J009C | Bacterial infections | Remaining infections | Rhinitis acuta |
| J01 | Bacterial infections | Remaining infections | Akut bihulebetændelse |
| J010 | Bacterial infections | Remaining infections | Sinuitis maxillaris acuta |
| J010A | Bacterial infections | Remaining infections | Empyema acuta sinus maxillaris |
| J010B | Bacterial infections | Remaining infections | Abscessus acutus sinus maxillaris |
| J011 | Bacterial infections | Remaining infections | Sinuitis frontalis acuta |
| J011A | Bacterial infections | Remaining infections | Empyema acuta sinus frontalis |
| J011B | Bacterial infections | Remaining infections | Abscessus acutus sinus frontalis |
| J012 | Bacterial infections | Remaining infections | Sinuitis ethmoidalis acuta |
| J012A | Bacterial infections | Remaining infections | Abscessus acutus sinus ethmoidalis |
| J012B | Bacterial infections | Remaining infections | Empyema acuta sinus ethmoidalis |
| J013 | Bacterial infections | Remaining infections | Sinuitis sphenoidalis acuta |
| J013A | Bacterial infections | Remaining infections | Empyema acuta sinus sphenoidalis |
| J013B | Bacterial infections | Remaining infections | Abscessus acutus sinus sphenoidalis |
| J014 | Bacterial infections | Remaining infections | Pansinuitis acuta |
| J014A | Bacterial infections | Remaining infections | Empyema acuta sinus nasi |
| J014B | Bacterial infections | Remaining infections | Abscessus acutus sinus nasi |
| J018 | Bacterial infections | Remaining infections | Sinuitis acuta, andre former |
| J019 | Bacterial infections | Remaining infections | Sinuitis acuta uden specifikation |
| J02 | Bacterial infections | Remaining infections | Akut svælgkatar |
| J020 | Bacterial infections | Remaining infections | Pharyngitis acuta streptococcica |
| J020A | Bacterial infections | Sepsis | Angina septica |
| J028 | Bacterial infections | Remaining infections | Akut faryngit forårsaget af andre specificerede organismer |
| J029 | Bacterial infections | Remaining infections | Pharyngitis acuta uden specifikation |
| J029A | Bacterial infections | Remaining infections | Pharyngitis acuta ulcerosa |
| J029B | Bacterial infections | Remaining infections | Pharyngitis acuta catarrhalis |
| J029C | Bacterial infections | Remaining infections | Pharyngitis acuta gangraenosa |
| J030 | Bacterial infections | Remaining infections | Tonsillitis acuta streptococcica |
| J038 | Bacterial infections | Remaining infections | Tonsillitis acuta forårsaget af andre specific organismer |
| J039A | Bacterial infections | Remaining infections | Tonsillitis acuta catarrhalis |
| J039B | Bacterial infections | Remaining infections | Tonsillitis acuta ulcerosa |
| J039C | Bacterial infections | Remaining infections | Tonsillitis acuta infectiosa |
| J039D | Bacterial infections | Remaining infections | Tonsillitis acuta gangraenosa |
| J039E | Bacterial infections | Remaining infections | Tonsillitis acuta follicularis |
| J039F | Bacterial infections | Remaining infections | Tonsillitis acuta recidivans |
| J04 | Bacterial infections | Infections in lower airways | Akut strube- og luftrørskatar |
| J040 | Bacterial infections | Infections in lower airways | Laryngitis acuta |
| J040A | Bacterial infections | Infections in lower airways | Laryngitis ulcerosa acuta |
| J040B | Bacterial infections | Infections in lower airways | Laryngitis subglottica acuta |
| J040C | Bacterial infections | Infections in lower airways | Laryngitis phlegmonosa acuta |
| J040D | Bacterial infections | Infections in lower airways | Laryngitis oedematosa acuta |
| J041 | Bacterial infections | Infections in lower airways | Tracheitis acuta |
| J042 | Bacterial infections | Infections in lower airways | Laryngotracheitis acuta |
| J042A | Bacterial infections | Infections in lower airways | Tracheolaryngitis acuta |
| J05 | Bacterial infections | Infections in lower airways | Pseudokrup og akut strubelågsbetændelse |
| J050 | Bacterial infections | Infections in lower airways | Laryngitis acuta stridulans (pseudocroup) |
| J051 | Bacterial infections | Remaining infections | Epiglottitis acuta |
| J06 | Bacterial infections | Remaining infections | Akut øvre luftvejsinfek m flere og ikke spec lokalisationer |
| J060 | Bacterial infections | Remaining infections | Laryngopharyngitis acuta |
| J068 | Bacterial infections | Remaining infections | Akutte øvre luftvejsinfek m flere lokalisationer, andre |
| J069 | Bacterial infections | Remaining infections | Akut øvre luftsvejsinfek uden specifikation |
| J13 | Bacterial infections | Infections in lower airways | Pneumokok-lungebetændelse |
| J139 | Bacterial infections | Infections in lower airways | Pneumonia pneumococcica |
| J139A | Bacterial infections | Infections in lower airways | Pneumonia lobaris pneumococcica |
| J139B | Bacterial infections | Infections in lower airways | Bronchopneumonia pneumococcica |
| J14 | Bacterial infections | Infections in lower airways | Hæmofilus-lungebetændelse |
| J149 | Bacterial infections | Infections in lower airways | Pneumoni forårsaget af Haemophilus influenzae |
| J149A | Bacterial infections | Infections in lower airways | Bronchopneumonia, Haemophilus influenzae |
| J149B | Bacterial infections | Infections in lower airways | Pneumonia lobaris, Haemophilus influenzae |
| J15 | Bacterial infections | Infections in lower airways | Bakteriel lungebetændelse ikke klassificeret andetsteds |
| J150 | Bacterial infections | Infections in lower airways | Pneumoni forårsaget af Klebsiella pneumoniae |
| J151 | Bacterial infections | Infections in lower airways | Pneumoni forårsaget af Pseudomonas |
| J152 | Bacterial infections | Infections in lower airways | Pneumonia staphylococcica |
| J153 | Bacterial infections | Infections in lower airways | Pneumonia streptococcica, gruppe B |
| J154 | Bacterial infections | Infections in lower airways | Pneumoni forårsaget af andre streptokokker |
| J155 | Bacterial infections | Infections in lower airways | Pneumoni forårsaget af Escherichia coli |
| J156 | Bacterial infections | Infections in lower airways | Pneumoni forårsaget af anden aerob gram-negativ bakterie |
| J156A | Bacterial infections | Infections in lower airways | Pneumonia, Serratia marcescens |
| J157 | Bacterial infections | Infections in lower airways | Pneumoni forårsaget af Mycoplasma pneumoniae |
| J158 | Bacterial infections | Infections in lower airways | Bakterielle pneumonier, andre |
| J159 | Bacterial infections | Infections in lower airways | Bakteriel pneumoni uden specifikation |
| J16 | Bacterial infections | Infections in lower airways | Lungebetændelse s.f.a. andet infek agens, ikke klass ansted |
| J168 | Bacterial infections | Infections in lower airways | Pneumonier forårsaget af andre specificerede infekt agentia |
| J17 | Bacterial infections | Infections in lower airways | Lungebetændelse ved sygdom klassificeret andetsteds |
| J170 | Bacterial infections | Infections in lower airways | Pneumoni ved bakterielle sygdomme klassificeret andetsteds |
| J170A | Bacterial infections | Infections in lower airways | Pneumonia, tularaemia |
| J170B | Bacterial infections | Infections in lower airways | Pneumonia typhosa |
| J170D | Bacterial infections | Infections in lower airways | Pneumonia actinomycotica |
| J170E | Bacterial infections | Infections in lower airways | Pneumonia, nocardiosis |
| J170F | Bacterial infections | Infections in lower airways | Pneumonia, anthrax |
| J170G | Bacterial infections | Infections in lower airways | Pneumonia, salmonella |
| J170H | Bacterial infections | Infections in lower airways | Pneumonia, tussis convulsiva |
| J171 | Bacterial infections | Infections in lower airways | Pneumoni ved virale sygdom klassificeret andetsteds |
| J171A | Bacterial infections | Infections in lower airways | Pneumonia, varicella |
| J171B | Bacterial infections | Infections in lower airways | Pneumonia, rubella |
| J171C | Bacterial infections | Infections in lower airways | Pneumonia, morbilli |
| J171D | Bacterial infections | Infections in lower airways | Pneumonia, cytomegalovirus |
| J172 | Bacterial infections | Infections in lower airways | Pneumoni ved mykoser klassificeret andetsteds |
| J172A | Bacterial infections | Infections in lower airways | Pneumonia, histoplasmosis |
| J172B | Bacterial infections | Infections in lower airways | Pneumonia, coccidiodomycosis |
| J172C | Bacterial infections | Infections in lower airways | Pneumonia, candidiasis |
| J172D | Bacterial infections | Infections in lower airways | Pneumonia, aspergillosis |
| J173 | Bacterial infections | Infections in lower airways | Pneumoni ved parasitære sygdom klassificeret andetsteds |
| J173A | Bacterial infections | Infections in lower airways | Pneumonia, toxoplasmosis |
| J173B | Bacterial infections | Infections in lower airways | Pneumonia, schistosomiasis |
| J173C | Bacterial infections | Infections in lower airways | Pneumonia, ascariasis |
| J178 | Bacterial infections | Infections in lower airways | Pneumoni ved andre sygdom klassificeret andetsteds |
| J178A | Bacterial infections | Infections in lower airways | Pneumonia rheumatica |
| J178B | Bacterial infections | Infections in lower airways | Pneumonia, Q feber |
| J178C | Bacterial infections | Infections in lower airways | Pneumonia, ornithosis |
| J18 | Bacterial infections | Infections in lower airways | Lungebetændelse, agens ikke specificeret |
| J180 | Bacterial infections | Infections in lower airways | Bronchopneumoni uden specifikation |
| J181 | Bacterial infections | Infections in lower airways | Pneumonia lobaris uden specifikation |
| J182 | Bacterial infections | Infections in lower airways | Pneumonia hypostatica uden specifikation |
| J188 | Bacterial infections | Infections in lower airways | Pneumoni, andre former agens ikke specificeret |
| J189 | Bacterial infections | Infections in lower airways | Pneumoni uden specifikation |
| J20 | Bacterial infections | Infections in lower airways | Akut bronkit |
| J200 | Bacterial infections | Infections in lower airways | Bronchitis acuta forårsaget af Mycoplasma pneumoniae |
| J200A | Bacterial infections | Infections in lower airways | Akut bronkitis f.a. Mycoplasma pneumoniae med bronkospasme |
| J201 | Bacterial infections | Infections in lower airways | Bronchitis acuta forårsaget af Haemophilus influenzae |
| J201A | Bacterial infections | Infections in lower airways | Akut bronkitis f.a. Haemophilus influenzae med bronkospasme |
| J202 | Bacterial infections | Infections in lower airways | Bronchitis acuta streptococcica |
| J202A | Bacterial infections | Infections in lower airways | Akut bronkitis forårsaget af streptokokker med bronkospasme |
| J22 | Bacterial infections | Infections in lower airways | Akut infektion i nedre luftveje uden specifikation |
| J229 | Bacterial infections | Infections in lower airways | Akut nedre luftsvejsinfek uden specifikation |
| J320 | Bacterial infections | Remaining infections | Sinuitis chronica maxillaris |
| J321 | Bacterial infections | Remaining infections | Sinuitis chronica frontalis |
| J322 | Bacterial infections | Remaining infections | Sinuitis chronica ethmoidalis |
| J323 | Bacterial infections | Remaining infections | Sinuitis chronica sphenoidalis |
| J324 | Bacterial infections | Remaining infections | Pansinuitis chronica |
| J328 | Bacterial infections | Remaining infections | Sinuitis chronica, andre former |
| J329 | Bacterial infections | Remaining infections | Sinuitis chronica uden specifikation |
| J340 | Bacterial infections | Remaining infections | Abscessus, furunculus et carbunculus nasi |
| J340A | Bacterial infections | Remaining infections | Abscessus nasi |
| J340B | Bacterial infections | Remaining infections | Carbunculus nasi |
| J340C | Bacterial infections | Remaining infections | Cellulitis nasi |
| J340D | Bacterial infections | Remaining infections | Phlegmone nasi |
| J340J | Bacterial infections | Remaining infections | Furunculus nasi |
| J350 | Bacterial infections | Remaining infections | Tonsillitis chronica |
| J36 | Bacterial infections | Remaining infections | Halsbyld |
| J369 | Bacterial infections | Remaining infections | Abscessus peritonsillaris |
| J37 | Bacterial infections | Infections in lower airways | Kronisk strubekatar og luftrørskatar |
| J370 | Bacterial infections | Infections in lower airways | Laryngitis chronica |
| J370A | Bacterial infections | Infections in lower airways | Laryngitis sicca |
| J370B | Bacterial infections | Infections in lower airways | Laryngitis catarrhalis |
| J370C | Bacterial infections | Infections in lower airways | Laryngitis hypertrophica |
| J371 | Bacterial infections | Infections in lower airways | Laryngotracheitis chronica |
| J383D | Bacterial infections | Remaining infections | Abscessus plicae vocalis |
| J390 | Bacterial infections | Remaining infections | Abscessus retropharyngealis et parapharyngealis |
| J390A | Bacterial infections | Remaining infections | Abscessus peripharyngealis |
| J390B | Bacterial infections | Remaining infections | Abscessus retropharyngealis |
| J390C | Bacterial infections | Remaining infections | Abscessus parapharyngealis |
| J391 | Bacterial infections | Remaining infections | Abscesser i pharynx, andre |
| J391A | Bacterial infections | Remaining infections | Abscessus pharyngis |
| J391B | Bacterial infections | Remaining infections | Abscessus nasopharyngealis |
| J398A | Bacterial infections | Infections in lower airways | Abscessus tracheae |
| J41 | Bacterial infections | Infections in lower airways | Simpel og mukopurulent kronisk bronkit |
| J411 | Bacterial infections | Infections in lower airways | Bronchitis chronica mucopurulenta |
| J418 | Bacterial infections | Infections in lower airways | Bronchitis chronica simplex et mucopurulenta, blandet type |
| J843 | Bacterial infections | Infections in lower airways | Bronchiolitis obliterans organiserende pneumoni (BOOP) |
| J85 | Bacterial infections | Infections in lower airways | Byld på lunge og i skillevæg mellem lunger |
| J850 | Bacterial infections | Infections in lower airways | Gangraena et necrosis pulmonis |
| J850A | Bacterial infections | Infections in lower airways | Gangraena pulmonis |
| J850B | Bacterial infections | Infections in lower airways | Necrosis pulmonis |
| J851 | Bacterial infections | Infections in lower airways | Lungeabsces m pneumoni |
| J852 | Bacterial infections | Infections in lower airways | Lungeabsces u pneumoni |
| J853 | Bacterial infections | Infections in lower airways | Abscessus mediastini |
| J86 | Bacterial infections | Infections in lower airways | Pus i lungehinde |
| J860 | Bacterial infections | Infections in lower airways | Empyema pleurae m fistel |
| J860A | Bacterial infections | Infections in lower airways | Pyopneumothorax med fistel |
| J869 | Bacterial infections | Infections in lower airways | Empyema pleurae u fistel |
| J869A | Bacterial infections | Infections in lower airways | Pyopneumothorax uden fistel |
| J942 | Non-traumatic bleedings | Remaining bleedings | Haemothorax |
| J985D | Bacterial infections | Remaining infections | Mediastinitis |
| J986D | Bacterial infections | Remaining infections | Diaphragmatitis |
| K040A | Bacterial infections | Remaining infections | Abscessus pulpae dentis |
| K041 | Bacterial infections | Remaining infections | Necrosis pulpae dentis |
| K041A | Bacterial infections | Remaining infections | Gangraena pulpae dentis |
| K044 | Bacterial infections | Remaining infections | Parodontitis apicalis acuta |
| K045 | Bacterial infections | Remaining infections | Parodontitis apicalis chronica |
| K046 | Bacterial infections | Remaining infections | Abscessus periapicalis dentis m fistel |
| K047 | Bacterial infections | Remaining infections | Abscessus periapicalis dentis u fistel |
| K047A | Bacterial infections | Remaining infections | Abscessus alveolaris dentis uden specifikation |
| K050 | Bacterial infections | Remaining infections | Gingivitis acuta |
| K051 | Bacterial infections | Remaining infections | Gingivitis chronica |
| K051A | Bacterial infections | Remaining infections | Gingivitis ulcerosa |
| K051B | Bacterial infections | Remaining infections | Gingivitis uden specifikation |
| K052A | Bacterial infections | Remaining infections | Abscessus parodontalis |
| K052B | Bacterial infections | Remaining infections | Parodontitis marginalis acuta |
| K052C | Bacterial infections | Remaining infections | Pericoronitis acuta |
| K053 | Bacterial infections | Remaining infections | Parodontitis chronica |
| K053A | Bacterial infections | Remaining infections | Parodontitis uden specifikation |
| K053B | Bacterial infections | Remaining infections | Pericoronitis chronica |
| K089A | Non-traumatic bleedings | Remaining bleedings | Haemorrhagia processus alveolaris |
| K102 | Bacterial infections | Infections in joints and bones | Osteomyelitis et periostitis mandibulae et maxillae |
| K102A | Bacterial infections | Infections in joints and bones | Osteitis (acuta)(chronica)(purulenta) maxillae |
| K102B | Bacterial infections | Infections in joints and bones | Osteitis (acuta)(chronica)(purulenta) mandibulae |
| K102C | Bacterial infections | Infections in joints and bones | Osteomyelitis (neonatalis)(acuta)(chron)(purulenta) mandibu |
| K102D | Bacterial infections | Infections in joints and bones | Osteomyelitis (neonatalis)(acuta)(chron)(purulenta) maxilla |
| K102G | Bacterial infections | Infections in joints and bones | Periostitis (acuta)(chronica)(purulenta) mandibulae |
| K102H | Bacterial infections | Infections in joints and bones | Periostitis (acuta)(chronica)(purulenta) maxillae |
| K103 | Bacterial infections | Infections in joints and bones | Ostitis alveolaris |
| K103A | Bacterial infections | Infections in joints and bones | Ostitis alveolaris |
| K103B | Bacterial infections | Infections in joints and bones | Periostitis alveolaris |
| K108I | Bacterial infections | Infections in joints and bones | Ostitis fibrosa mandibulae |
| K108J | Bacterial infections | Infections in joints and bones | Ostitis fibrosa maxillae |
| K112A | Bacterial infections | Remaining infections | Parotitis purulenta |
| K112B | Bacterial infections | Remaining infections | Parotitis chronica |
| K112C | Bacterial infections | Remaining infections | Parotitis acuta |
| K113 | Bacterial infections | Remaining infections | Abscessus glandulae salivaris |
| K113A | Bacterial infections | Remaining infections | Abscessus glandulae submaxillaris |
| K113B | Bacterial infections | Remaining infections | Abscessus glandulae sublingualis |
| K113C | Bacterial infections | Remaining infections | Abscessus glandulae submandibularis |
| K113D | Bacterial infections | Remaining infections | Abscessus glandulae parotidis |
| K121C | Bacterial infections | Remaining infections | Stomatitis catarrhalis |
| K122 | Bacterial infections | Remaining infections | Phlegmone et abscessus oris |
| K122A | Bacterial infections | Remaining infections | Abscessus submandibularis |
| K122B | Bacterial infections | Remaining infections | Abscessus oris |
| K130A | Bacterial infections | Remaining infections | Abscessus labii oris |
| K140A | Bacterial infections | Remaining infections | Abscessus linguae |
| K140B | Bacterial infections | Remaining infections | Papillitis linguae |
| K20 | Bacterial infections | Remaining infections | Betændelse i spiserøret |
| K209 | Bacterial infections | Remaining infections | Oesophagitis |
| K209A | Bacterial infections | Remaining infections | Abscessus oesophagi |
| K228F | Bacterial infections | Remaining infections | Haemorrhagia oesophagi uden specifikation |
| K230 | Bacterial infections | Remaining infections | Oesophagitis tuberculosa |
| K25 | Bacterial infections | Remaining infections | Mavesår |
| K250 | Non-traumatic bleedings | Bleedings in the GI tract | Ulcus ventriculi acutum m blødning |
| K250A | Non-traumatic bleedings | Bleedings in the GI tract | Ulcus cardiae, akut med blødning |
| K250B | Non-traumatic bleedings | Bleedings in the GI tract | Ulcus corporis ventriculi, akut med blødning |
| K250C | Non-traumatic bleedings | Bleedings in the GI tract | Ulcus juxtapyloricum, akut med blødning |
| K250D | Non-traumatic bleedings | Bleedings in the GI tract | Ulcus praepyloricum, akut med blødning |
| K250E | Non-traumatic bleedings | Bleedings in the GI tract | Ulcus pylori, akut med blødning |
| K250F | Non-traumatic bleedings | Bleedings in the GI tract | Exulceratio simplex Dieulafoy med blødning |
| K251 | Bacterial infections | Remaining infections | Ulcus ventriculi acutum m perforation |
| K251A | Bacterial infections | Remaining infections | Ulcus cardiae, akut med perforation |
| K251B | Bacterial infections | Remaining infections | Ulcus pylori, akut med perforation |
| K251C | Bacterial infections | Remaining infections | Ulcus corporis ventriculi, akut med perforation |
| K251D | Bacterial infections | Remaining infections | Ulcus juxtapyloricum, akut med perforation |
| K251E | Bacterial infections | Remaining infections | Ulcus praepyloricum, akut med perforation |
| K252 | Non-traumatic bleedings | Bleedings in the GI tract | Ulcus ventriculi acutum m både blødning og perforation |
| K252A | Non-traumatic bleedings | Bleedings in the GI tract | Ulcus cardiae, akut med både blødning og perforation |
| K252B | Non-traumatic bleedings | Bleedings in the GI tract | Ulcus pylori, akut med både blødning og perforation |
| K252C | Non-traumatic bleedings | Bleedings in the GI tract | Ulcus corporis ventriculi, akut med både blødning og perfor |
| K252D | Non-traumatic bleedings | Bleedings in the GI tract | Ulcus juxtapyloricum, akut med både blødning og perforation |
| K252E | Non-traumatic bleedings | Bleedings in the GI tract | Ulcus praepyloricum, akut med både blødning og perforation |
| K253 | Bacterial infections | Remaining infections | Ulcus ventriculi acutum u blødning eller perforation |
| K253A | Bacterial infections | Remaining infections | Ulcus cardiae, akut uden blødning eller perforation |
| K253B | Bacterial infections | Remaining infections | Ulcus pylori, akut uden blødning eller perforation |
| K253C | Bacterial infections | Remaining infections | Ulcus corporis ventriculi, akut uden blødning eller perfor |
| K253D | Bacterial infections | Remaining infections | Ulcus juxtapyloricum, akut uden blødning eller perforation |
| K253E | Bacterial infections | Remaining infections | Ulcus praepyloricum, akut uden blødning eller perforation |
| K254 | Non-traumatic bleedings | Bleedings in the GI tract | Ulcus ventriculi chronicum eller u spec m blødning |
| K254A | Non-traumatic bleedings | Bleedings in the GI tract | Ulcus cardiae, kronisk med blødning |
| K254B | Non-traumatic bleedings | Bleedings in the GI tract | Ulcus cardiae uden specifikation med blødning |
| K254C | Non-traumatic bleedings | Bleedings in the GI tract | Ulcus pylori, kronisk med blødning |
| K254D | Non-traumatic bleedings | Bleedings in the GI tract | Ulcus pylori uden specifikation, med blødning |
| K254E | Non-traumatic bleedings | Bleedings in the GI tract | Ulcus corporis ventriculi, kronisk med blødning |
| K254F | Non-traumatic bleedings | Bleedings in the GI tract | Ulcus corporis ventriculi, u specifikation med blødning |
| K254G | Non-traumatic bleedings | Bleedings in the GI tract | Ulcus juxtapyloricum, kronisk med blødning |
| K254H | Non-traumatic bleedings | Bleedings in the GI tract | Ulcus juxtapyloricum, uden specifikation med blødning |
| K254I | Non-traumatic bleedings | Bleedings in the GI tract | Ulcus praepyloricum, kronisk med blødning |
| K254J | Non-traumatic bleedings | Bleedings in the GI tract | Ulcus praepyloricum, uden specifikation med blødning |
| K255 | Bacterial infections | Remaining infections | Ulcus ventriculi chronicum eller u spec m perforation |
| K255A | Bacterial infections | Remaining infections | Ulcus cardiae, kronisk med perforation |
| K255B | Bacterial infections | Remaining infections | Ulcus cardiae uden specifikation med perforation |
| K255C | Bacterial infections | Remaining infections | Ulcus pylori, kronisk med perforation |
| K255D | Bacterial infections | Remaining infections | Ulcus pylori uden specifikation med perforation |
| K255E | Bacterial infections | Remaining infections | Ulcus corporis ventriculi, kronisk med perforation |
| K255F | Bacterial infections | Remaining infections | Ulcus corporis ventriculi u specifikation med perforation |
| K255G | Bacterial infections | Remaining infections | Ulcus juxtapyloricum, kronisk med perforation |
| K255H | Bacterial infections | Remaining infections | Ulcus juxtapyloricum uden specifikation med perforation |
| K255I | Bacterial infections | Remaining infections | Ulcus praepyloricum, kronisk med perforation |
| K255J | Bacterial infections | Remaining infections | Ulcus praepyloricum uden specifikation med perforation |
| K256 | Non-traumatic bleedings | Bleedings in the GI tract | Ulcus ventr chr eller u spec m både blødning og perforation |
| K256A | Non-traumatic bleedings | Bleedings in the GI tract | Ulcus cardiae, kronisk med blødning og perforation |
| K256B | Non-traumatic bleedings | Bleedings in the GI tract | Ulcus cardiae, u specifikation, med blødning og perforation |
| K256C | Non-traumatic bleedings | Bleedings in the GI tract | Ulcus pylori, kronisk med blødning og perforation |
| K256D | Non-traumatic bleedings | Bleedings in the GI tract | Ulcus pylori, uden specifikation med blødning og perforatio |
| K256E | Non-traumatic bleedings | Bleedings in the GI tract | Ulcus corporis ventriculi, kronisk m blødning og perforatio |
| K256F | Non-traumatic bleedings | Bleedings in the GI tract | Ulcus corporis ventriculi u spec med blødning og perforatio |
| K256G | Non-traumatic bleedings | Bleedings in the GI tract | Ulcus juxtapyloricum, kronisk med blødning og perforation |
| K256H | Non-traumatic bleedings | Bleedings in the GI tract | Ulcus juxtapyloricum, u specif med blødning og perforation |
| K256I | Non-traumatic bleedings | Bleedings in the GI tract | Ulcus praepyloricum, kronisk med blødning og perforation |
| K256J | Non-traumatic bleedings | Bleedings in the GI tract | Ulcus praepyloricum, u specif med blødning og perforation |
| K257 | Bacterial infections | Remaining infections | Ulcus ventriculi chronicum u blødning eller perforation |
| K257A | Bacterial infections | Remaining infections | Ulcus cardiae, kronisk uden blødning eller perforation |
| K257B | Bacterial infections | Remaining infections | Ulcus pylori, kronisk uden blødning eller perforation |
| K257C | Bacterial infections | Remaining infections | Ulcus corporis ventriculi, kronisk u blødn el perforation |
| K257D | Bacterial infections | Remaining infections | Ulcus juxtapyloricum, kronisk uden blødning el perforation |
| K257E | Bacterial infections | Remaining infections | Ulcus praepyloricum, kronisk uden blødning el perforation |
| K259 | Bacterial infections | Remaining infections | Mavesår u spec u blødning eller perforation |
| K259A | Bacterial infections | Remaining infections | Ulcus corporis ventriculi uden specifikation |
| K26 | Bacterial infections | Remaining infections | Sår på tolvfingertarm |
| K260 | Non-traumatic bleedings | Bleedings in the GI tract | Ulcus duodeni acutum m blødning |
| K260A | Non-traumatic bleedings | Bleedings in the GI tract | Ulcus postpyloricum, akut med blødning |
| K261 | Bacterial infections | Remaining infections | Ulcus duodeni acutum m perforation |
| K261A | Bacterial infections | Remaining infections | Ulcus postpyloricum, akut med perforation |
| K262 | Non-traumatic bleedings | Bleedings in the GI tract | Ulcus duodeni acutum m både blødning og perforation |
| K262A | Non-traumatic bleedings | Bleedings in the GI tract | Ulcus postpyloricum, akut med både blødning og perforation |
| K263 | Bacterial infections | Remaining infections | Ulcus duodeni acutum u blødning eller perforation |
| K263A | Bacterial infections | Remaining infections | Ulcus postpyloricum, akut uden blødning eller perforation |
| K264 | Non-traumatic bleedings | Bleedings in the GI tract | Ulcus duodeni chronicum eller u spec, m blødning |
| K264A | Non-traumatic bleedings | Bleedings in the GI tract | Ulcus duodeni, kronisk med blødning |
| K264B | Non-traumatic bleedings | Bleedings in the GI tract | Ulcus duodeni uden specifikation med blødning |
| K264C | Non-traumatic bleedings | Bleedings in the GI tract | Ulcus postpyloricum, kronisk med blødning |
| K264D | Non-traumatic bleedings | Bleedings in the GI tract | Ulcus postpyloricum uden specifikation med blødning |
| K265 | Bacterial infections | Remaining infections | Ulcus duodeni chronicum eller u spec, m perforation |
| K265A | Bacterial infections | Remaining infections | Ulcus duodeni, kronisk spec med perforation |
| K265B | Bacterial infections | Remaining infections | Ulcus duodeni uden specifikation med perforation |
| K265C | Bacterial infections | Remaining infections | Ulcus postpyloricum, kronisk med perforation |
| K265D | Bacterial infections | Remaining infections | Ulcus postpyloricum uden specifikation med perforation |
| K266 | Non-traumatic bleedings | Bleedings in the GI tract | Ulcus duodeni chr eller u spec, m både blødning og perf |
| K266A | Non-traumatic bleedings | Bleedings in the GI tract | Ulcus duodeni, kronisk med blødning og perforation |
| K266B | Non-traumatic bleedings | Bleedings in the GI tract | Ulcus duodeni u specifikation med blødning og perforation |
| K266C | Non-traumatic bleedings | Bleedings in the GI tract | Ulcus postpyloricum, kronisk med blødning og perforation |
| K266D | Non-traumatic bleedings | Bleedings in the GI tract | Ulcus postpyloricum u spec med blødning og perforation |
| K267 | Bacterial infections | Remaining infections | Ulcus duodeni chronicum u blødning eller perforation |
| K267A | Bacterial infections | Remaining infections | Ulcus postpyloricum, kronisk uden blødning el perforation |
| K267B | Bacterial infections | Remaining infections | Ulcus duodeni, kronisk uden blødning eller perforation |
| K269 | Bacterial infections | Remaining infections | Ulcus duodeni chronicum u spec u blødning eller perforation |
| K27 | Bacterial infections | Remaining infections | Mavesår eller sår på tolvfingertarm |
| K270 | Non-traumatic bleedings | Bleedings in the GI tract | Ulcus gastroduodenale acutum u angivet lokalis m blødning |
| K271 | Bacterial infections | Remaining infections | Ulcus gastroduodenale acutum u angivet lokalis m perforatio |
| K272 | Non-traumatic bleedings | Bleedings in the GI tract | Ulcus gastroduod acutum u ang lokalis m blødning og perfor |
| K273 | Bacterial infections | Remaining infections | Ulcus gastroduodenale ac u ang lokalis u blødning ell perf |
| K274 | Non-traumatic bleedings | Bleedings in the GI tract | Ulcus Gastroduodenale u ang lok. kronisk el u spec m blødn |
| K275 | Bacterial infections | Remaining infections | Ulcus gastroduodenale u ang lok. kronisk el u spec m perf |
| K276 | Non-traumatic bleedings | Bleedings in the GI tract | Ulcus gastroduod u ang lok. kron ell u spec m blød og perf |
| K277 | Bacterial infections | Remaining infections | Ulcus gastroduod u ang lokalis kron u blødning eller perf |
| K279 | Bacterial infections | Remaining infections | Ulcus gastroduod u spec u ang af blødning eller perforation |
| K28 | Bacterial infections | Remaining infections | Anastomosesår |
| K280 | Non-traumatic bleedings | Bleedings in the GI tract | Ulcus gastrojejunale pepticum acutum m blødning |
| K280A | Non-traumatic bleedings | Bleedings in the GI tract | Ulcus gastrointestinale pepticum, akut med blødning |
| K280B | Non-traumatic bleedings | Bleedings in the GI tract | Ulcus gastrocolicum pepticum, akut med blødning |
| K281 | Bacterial infections | Remaining infections | Ulcus gastrojejunale pepticum acutum m perforation |
| K281A | Bacterial infections | Remaining infections | Ulcus jejunale pepticum, akut med perforation |
| K281B | Bacterial infections | Remaining infections | Ulcus gastrointestinale pepticum, akut med perforation |
| K281C | Bacterial infections | Remaining infections | Ulcus gastrocolicum pepticum, akut med perforation |
| K282 | Non-traumatic bleedings | Bleedings in the GI tract | Ulcus gastrojejunale pepticum acutum m både blødning og per |
| K282A | Non-traumatic bleedings | Bleedings in the GI tract | Ulcus jejunale pepticum, akut med blødning og perforation |
| K282B | Non-traumatic bleedings | Bleedings in the GI tract | Ulcus gastrointestinale pept, akut med blødn og perforation |
| K282C | Non-traumatic bleedings | Bleedings in the GI tract | Ulcus gastrocolicum pept, akut med blødning og perforation |
| K283 | Bacterial infections | Remaining infections | Ulcus gastrojejunale pepticum acutum u blødning eller perf |
| K283A | Bacterial infections | Remaining infections | Ulcus jejunale pepticum, akut uden blødning el perforation |
| K283B | Bacterial infections | Remaining infections | Ulcus gastrointest pept, akut uden blødning el perforation |
| K283C | Bacterial infections | Remaining infections | Ulcus gastrocolicum pept, akut uden blødning el perforation |
| K284 | Non-traumatic bleedings | Bleedings in the GI tract | Ulcus gastrojejunale pepticum chr eller u spec m blødning |
| K284A | Non-traumatic bleedings | Bleedings in the GI tract | Ulcus gastrointestinale pepticum, kronisk med blødning |
| K284B | Non-traumatic bleedings | Bleedings in the GI tract | Ulcus gastrointestinale pepticum, u specifikation m blødnin |
| K284C | Non-traumatic bleedings | Bleedings in the GI tract | Ulcus gastrocolicum pepticum, kronisk med blødning |
| K284D | Non-traumatic bleedings | Bleedings in the GI tract | Ulcus gastrocolicum pepticum u specifikation, med blødning |
| K284E | Non-traumatic bleedings | Bleedings in the GI tract | Ulcus jejunale pepticum, kronisk med blødning |
| K284F | Non-traumatic bleedings | Bleedings in the GI tract | Ulcus jejunale pepticum uden specifikation med blødning |
| K285 | Bacterial infections | Remaining infections | Ulcus gastrojejunale pepticum chr eller u spec m perforatio |
| K285A | Bacterial infections | Remaining infections | Ulcus gastrointestinale pepticum, kronisk med perforation |
| K285B | Bacterial infections | Remaining infections | Ulcus gastrointestinale pepticum u spec, med perforation |
| K285C | Bacterial infections | Remaining infections | Ulcus gastrocolicum pepticum, kronisk med perforation |
| K285D | Bacterial infections | Remaining infections | Ulcus gastrocolicum pepticum, u specifikation m perforation |
| K285E | Bacterial infections | Remaining infections | Ulcus jejunale pepticum, kronisk med perforation |
| K285F | Bacterial infections | Remaining infections | Ulcus jejunale pepticum u spec med perforation |
| K286 | Non-traumatic bleedings | Bleedings in the GI tract | Ulcus gastrojejunale pept chr el u spec m blødning og perf |
| K286A | Non-traumatic bleedings | Bleedings in the GI tract | Ulcus gastrointest pept, kronisk m blødning og perforation |
| K286B | Non-traumatic bleedings | Bleedings in the GI tract | Ulcus gastrointest pept u spec, m blødning og perforation |
| K286C | Non-traumatic bleedings | Bleedings in the GI tract | Ulcus gastrocolicum pept, kronisk m blødning og perforation |
| K286D | Non-traumatic bleedings | Bleedings in the GI tract | Ulcus gastrocolicum pept u spec, m blødning og perforation |
| K286E | Non-traumatic bleedings | Bleedings in the GI tract | Ulcus jejunale pepticum, kronisk m blødning og perforation |
| K286F | Non-traumatic bleedings | Bleedings in the GI tract | Ulcus jejunale pepticum u spec m blødning og perforation |
| K287 | Bacterial infections | Remaining infections | Ulcus gastrojejunale pepticum chr u blødning eller perf |
| K287A | Bacterial infections | Remaining infections | Ulcus gastrointest pept, kronisk u blødning el perforation |
| K287B | Bacterial infections | Remaining infections | Ulcus gastrocolicum pept, kronisk u blødning el perforation |
| K287C | Bacterial infections | Remaining infections | Ulcus jejunale pepticum, kronisk u blødning el perforation |
| K289 | Bacterial infections | Remaining infections | Ulcus gastrojejun pept u spec u ang af blødning ell perfor |
| K290 | Non-traumatic bleedings | Bleedings in the GI tract | Gastritis acuta haemorrhagica |
| K298 | Bacterial infections | Remaining infections | Duodenitis |
| K298A | Non-traumatic bleedings | Bleedings in the GI tract | Duodenitis acuta haemorrhagica |
| K299 | Bacterial infections | Remaining infections | Gastroduodenitis uden specifikation |
| K35 | Bacterial infections | Remaining infections | Akut blindtarmsbetændelse |
| K350 | Bacterial infections | Remaining infections | Appendicitis acuta m diffus peritonitit |
| K350A | Bacterial infections | Remaining infections | Appendicitis acuta perforans |
| K351 | Bacterial infections | Remaining infections | Appendicitis acuta m peritoneal absces |
| K351A | Bacterial infections | Remaining infections | Abscessus periappendicularis |
| K352 | Bacterial infections | Remaining infections | Akut appendicitis med generaliseret peritonitis |
| K353 | Bacterial infections | Remaining infections | Akut appendicitis med lokaliseret peritonitis |
| K353A | Bacterial infections | Remaining infections | Akut appendicitis med peritoneal absces |
| K353B | Bacterial infections | Remaining infections | Akut appendicitis med periappendikulær absces |
| K358 | Bacterial infections | Remaining infections | Anden og ikke spec. akut appendicitis |
| K358A | Bacterial infections | Remaining infections | Akut appendicitis UNS |
| K358B | Bacterial infections | Remaining infections | Akut gangrænøs appendicitis |
| K358C | Bacterial infections | Remaining infections | Akut flegmonøs appendicitis |
| K359 | Bacterial infections | Remaining infections | Akut blindtarmsbetændelse uden specifikation |
| K359A | Bacterial infections | Remaining infections | Appendicitis acuta gangraenosa |
| K359B | Bacterial infections | Remaining infections | Appendicitis acuta phlegmonosa |
| K36 | Bacterial infections | Remaining infections | Blindtarmsbetændelse, anden form |
| K369 | Bacterial infections | Remaining infections | Appendicitis chronica, recidivans |
| K37 | Bacterial infections | Remaining infections | Blindtarmsbetændelse uden specifikation |
| K379 | Bacterial infections | Remaining infections | Blindtarmsbetændelse uden specifikation |
| K550A | Other vascular conditions | Remaining other vascular conditions | Colitis acuta ischaemica fulminans |
| K550B | Other vascular conditions | Remaining other vascular conditions | Colitis ischaemica subacuta |
| K550C | Thromboembolic conditions | Remaining thromboembolisms | Embolia mesenterica |
| K550D | Thromboembolic conditions | Remaining thromboembolisms | Infarctio intestinalis acuta |
| K550E | Thromboembolic conditions | Remaining thromboembolisms | Infarctio mesenterialis |
| K550F | Thromboembolic conditions | Remaining thromboembolisms | Ischaemia acuta intestini tenuis |
| K550G | Other vascular conditions | Remaining other vascular conditions | Karsygdom i tarm, akut |
| K550H | Thromboembolic conditions | Remaining thromboembolisms | Thrombosis mesenterica |
| K551 | Other vascular conditions | Remaining other vascular conditions | Kroniske karsygdom i tarm |
| K551A | Other vascular conditions | Remaining other vascular conditions | Atherosclerosis mesenterica |
| K551B | Other vascular conditions | Remaining other vascular conditions | Colitis chronica ischaemica |
| K551C | Other vascular conditions | Remaining other vascular conditions | Enteritis chronica ischaemica |
| K551D | Other vascular conditions | Remaining other vascular conditions | Enterocolitis chronica ischaemica |
| K551E | Other vascular conditions | Remaining other vascular conditions | Insufficientia vasorum mesenterialium |
| K551F | Other vascular conditions | Remaining other vascular conditions | Strictura ischaemica intestini |
| K57 | Bacterial infections | Remaining infections | Udposning og betændelse af udposning på tarm |
| K570 | Bacterial infections | Remaining infections | Diverticulosis diverticulit intest tenuis m perf og absces |
| K570A | Bacterial infections | Remaining infections | Diverticulitis intestini tenuis med absces |
| K570B | Bacterial infections | Remaining infections | Diverticulitis intestini tenuis med perforation |
| K570C | Bacterial infections | Remaining infections | Diverticulitis intestini tenuis med peritonitis |
| K571 | Bacterial infections | Remaining infections | Diverticulosis diverticulit intest tenuis u perf ell absces |
| K571A | Bacterial infections | Remaining infections | Diverticulitis intestini tenuis uden perforation |
| K571B | Bacterial infections | Remaining infections | Diverticulitis intestini tenuis uden specifikation |
| K572 | Bacterial infections | Remaining infections | Diverticulosis diverticulit coli m perf og absces |
| K572A | Bacterial infections | Remaining infections | Diverticulitis coli med absces |
| K572B | Bacterial infections | Remaining infections | Diverticulitis coli med perforation |
| K572C | Bacterial infections | Remaining infections | Diverticulitis coli med peritonitis |
| K573 | Bacterial infections | Remaining infections | Diverticulosis diverticulit coli u perf eller absces |
| K573A | Bacterial infections | Remaining infections | Diverticulitis coli uden perforation |
| K573B | Bacterial infections | Remaining infections | Diverticulitis coli uden specifikation |
| K573C | Bacterial infections | Remaining infections | Diverticulum coli uden perforation |
| K573D | Bacterial infections | Remaining infections | Diverticulum coli uden specifikation |
| K573E | Bacterial infections | Remaining infections | Pericolitis |
| K573F | Bacterial infections | Remaining infections | Perisigmoiditis |
| K574 | Bacterial infections | Remaining infections | Diverticulosis i tynd- og tyktarm m perforation og absces |
| K578 | Bacterial infections | Remaining infections | Diverticulosis i tarm m perforation og absces, lokal.uspec. |
| K579A | Bacterial infections | Remaining infections | Peridiverticulitis |
| K61 | Bacterial infections | Remaining infections | Byld ved og omkring endetarm |
| K610 | Bacterial infections | Remaining infections | Abscessus ani |
| K610A | Bacterial infections | Remaining infections | Abscessus perianalis |
| K610B | Bacterial infections | Remaining infections | Phlegmone ani |
| K611 | Bacterial infections | Remaining infections | Abscessus recti |
| K611A | Bacterial infections | Remaining infections | Abscessus perirectalis |
| K612 | Bacterial infections | Remaining infections | Abscessus anorectalis |
| K613 | Bacterial infections | Remaining infections | Abscessus ischiorectalis |
| K614 | Bacterial infections | Remaining infections | Abscessus intrasphinctericus ani |
| K62 | Bacterial infections | Remaining infections | Sygdom i anus og rektum, andre |
| K625 | Non-traumatic bleedings | Bleedings in the GI tract | Haemorrhagia ani et recti |
| K625A | Non-traumatic bleedings | Bleedings in the GI tract | Haemorrhagia ani |
| K625B | Non-traumatic bleedings | Bleedings in the GI tract | Haemorrhagia recti |
| K628N | Bacterial infections | Remaining infections | Streptococcosis, anal |
| K630 | Bacterial infections | Remaining infections | Abscessus intestini |
| K638B | Non-traumatic bleedings | Bleedings in the GI tract | Haemorrhagia intestinalis |
| K638C | Non-traumatic bleedings | Bleedings in the GI tract | Haemorrhagia tractus gastrointestinalis |
| K65 | Bacterial infections | Remaining infections | Bughindebetændelse |
| K650 | Bacterial infections | Remaining infections | Peritonitis acuta |
| K650A | Bacterial infections | Remaining infections | Abscessus abdominis |
| K650B | Bacterial infections | Remaining infections | Abscessus bursae omentalis |
| K650C | Bacterial infections | Remaining infections | Abscessus fossae rectovesicalis |
| K650D | Bacterial infections | Remaining infections | Abscessus intraperitonealis |
| K650E | Bacterial infections | Remaining infections | Abscessus mesenterialis |
| K650F | Bacterial infections | Remaining infections | Abscessus omenti |
| K650G | Bacterial infections | Remaining infections | Abscessus pelvis |
| K650H | Bacterial infections | Remaining infections | Abscessus rectovaginalis |
| K650I | Bacterial infections | Remaining infections | Abscessus retrocoecalis |
| K650J | Bacterial infections | Remaining infections | Abscessus retroperitonealis |
| K650K | Bacterial infections | Remaining infections | Abscessus subdiaphragmaticus |
| K650L | Bacterial infections | Remaining infections | Abscessus subhepaticus |
| K650M | Bacterial infections | Remaining infections | Peritonitis (acuta) diffusa |
| K650N | Bacterial infections | Remaining infections | Peritonitis (acuta) purulenta |
| K650O | Bacterial infections | Remaining infections | Peritonitis (acuta) subphrenica |
| K650P | Bacterial infections | Remaining infections | Peritonitis localisata |
| K658 | Bacterial infections | Remaining infections | Peritonit, andre former |
| K658D | Bacterial infections | Remaining infections | Peritonitis, galde |
| K658E | Bacterial infections | Remaining infections | Peritonitis proliferativa chronica |
| K658F | Bacterial infections | Remaining infections | Peritonitis, urin |
| K658I | Bacterial infections | Remaining infections | Peritonitis, spontan bakteriel |
| K659 | Bacterial infections | Remaining infections | Peritonit uden specifikation |
| K661 | Non-traumatic bleedings | Remaining bleedings | Haemoperitoneum |
| K67 | Bacterial infections | Remaining infections | Sygdom i bughinde ved infektiøs sygdom klassific andetsteds |
| K673 | Bacterial infections | Remaining infections | Peritonitis tuberculosa |
| K75 | Bacterial infections | Remaining infections | Betændelse i lever, andre former |
| K750 | Bacterial infections | Remaining infections | Abscessus hepatis |
| K750A | Bacterial infections | Remaining infections | Abscessus hepatis haematogenes |
| K750B | Bacterial infections | Remaining infections | Abscessus hepatis lymphogenes |
| K750C | Bacterial infections | Remaining infections | Abscessus hepatis med cholangitis |
| K750D | Bacterial infections | Remaining infections | Abscessus hepatis med pylephlebitis |
| K751 | Bacterial infections | Remaining infections | Pylephlebitis |
| K751A | Bacterial infections | Remaining infections | Phlebitis venae portae |
| K763 | Thromboembolic conditions | Remaining thromboembolisms | Infarctus hepatis |
| K766B | Non-traumatic bleedings | Bleedings in the GI tract | Portal hypertensiv gastropati med blødning |
| K800 | Bacterial infections | Remaining infections | Cholecystolithiasis med cholecystitis acuta |
| K800A | Bacterial infections | Remaining infections | Calculus ductus cystici med cholecystitis acuta |
| K800B | Bacterial infections | Remaining infections | Calculus vesicae felleae med cholecystitis acuta |
| K800C | Bacterial infections | Remaining infections | Cholecystolithiasis m cholecystitis acuta |
| K800D | Bacterial infections | Remaining infections | Colica vesicae felleae med cholecystitis acuta |
| K801 | Bacterial infections | Remaining infections | Cholecystolithiasis med cholecystitis chronica |
| K801A | Bacterial infections | Remaining infections | Calculus ductus cystici med cholecystitis chronica |
| K801B | Bacterial infections | Remaining infections | Calculus vesicae felleae med cholecystitis chronica |
| K801D | Bacterial infections | Remaining infections | Cholecystolithiasis med cholecystitis chronica |
| K803 | Bacterial infections | Remaining infections | Cholelithiasis m cholangit |
| K803A | Bacterial infections | Remaining infections | Calculus ductus choledochi med cholangitis |
| K803B | Bacterial infections | Remaining infections | Calculus ductus hepatici med cholangitis |
| K803C | Bacterial infections | Remaining infections | Colica hepatis med cholangitis |
| K804 | Bacterial infections | Remaining infections | Cholelithiasis med cholecystit (med cholangit) |
| K804A | Bacterial infections | Remaining infections | Calculus ductus choledochi med cholangitis og cholecystitis |
| K804B | Bacterial infections | Remaining infections | Calculus ductus choledochi med cholecystitis |
| K804C | Bacterial infections | Remaining infections | Calculus ductus hepatici med cholangitis og cholecystitis |
| K804D | Bacterial infections | Remaining infections | Calculus ductus hepatici med cholecystitis |
| K804E | Bacterial infections | Remaining infections | Colica hepatis med både cholangitis og cholecystitis |
| K81 | Bacterial infections | Remaining infections | Galdeblærebetændelse |
| K810 | Bacterial infections | Remaining infections | Cholecystitis acuta |
| K810A | Bacterial infections | Remaining infections | Abscessus vesicae felleae (calculus non indicatus) |
| K810B | Bacterial infections | Remaining infections | Cholecystitis gangrenosa (calculus non indicatus) |
| K810C | Bacterial infections | Remaining infections | Cholecystitis purulenta (calculus non indicatus) |
| K811 | Bacterial infections | Remaining infections | Cholecystitis chronica |
| K818 | Bacterial infections | Remaining infections | Cholecystit, anden form |
| K819 | Bacterial infections | Remaining infections | Cholecystit uden specifikation |
| K830 | Bacterial infections | Remaining infections | Cholangitis |
| K830A | Bacterial infections | Remaining infections | Cholangitis ascendens |
| K830B | Bacterial infections | Remaining infections | Cholangitis primaria |
| K830C | Bacterial infections | Remaining infections | Cholangitis purulenta |
| K830D | Bacterial infections | Remaining infections | Cholangitis recurrens |
| K830E | Bacterial infections | Remaining infections | Cholangitis secundaria |
| K830F | Bacterial infections | Remaining infections | Cholangitis, primær scleroserende |
| K830G | Bacterial infections | Remaining infections | Cholangitis, sekundær scleroserende |
| K838F | Non-traumatic bleedings | Remaining bleedings | Blødning i galdegang |
| K85 | Bacterial infections | Remaining infections | Akut betændelse i bugspytkirtel |
| K850 | Bacterial infections | Remaining infections | Idiopatisk akut pankreatitis |
| K851 | Bacterial infections | Remaining infections | Akut pankreatitis forårsaget af galdevejslidelse |
| K851A | Bacterial infections | Remaining infections | Galdestenspankreatitis |
| K858 | Bacterial infections | Remaining infections | Anden form for akut pankreatitis |
| K858A | Bacterial infections | Remaining infections | Pankreasabsces |
| K858B | Bacterial infections | Remaining infections | Akut hæmoragisk pankreatitis |
| K858C | Bacterial infections | Remaining infections | Akut (adipøs) pankreasnekrose |
| K858D | Bacterial infections | Remaining infections | Subakut pankreatitis |
| K858E | Bacterial infections | Remaining infections | Infektiøs pankreasnekrose |
| K858F | Bacterial infections | Remaining infections | Recidiverende akut pankreatitis |
| K859 | Bacterial infections | Remaining infections | Pancreatitis acuta |
| K859A | Bacterial infections | Remaining infections | Abscessus pancreatis |
| K859B | Bacterial infections | Remaining infections | Pancreatitis acuta haemorrhagica |
| K859D | Bacterial infections | Remaining infections | Pancreatitis subacuta |
| K859E | Bacterial infections | Remaining infections | Necrosis pancreatis (adiposa) infectiosa |
| K859F | Bacterial infections | Remaining infections | Pancreatitis acuta recidivans |
| K861A | Bacterial infections | Remaining infections | Pancreatitis chronica infectiosa |
| K861D | Bacterial infections | Remaining infections | Pancreatitis recidivans |
| K868G | Non-traumatic bleedings | Remaining bleedings | Haemorrhagia pancreatis |
| K920 | Non-traumatic bleedings | Bleedings in the GI tract | Haematemesis |
| K921 | Non-traumatic bleedings | Bleedings in the GI tract | Melaena |
| K922 | Non-traumatic bleedings | Bleedings in the GI tract | Haemorrhagia gastrointestinalis uden specifikation |
| K930 | Bacterial infections | Remaining infections | Tuberculosis intestini et mesenterii |
| K930A | Bacterial infections | Remaining infections | Tuberculosis intestini |
| K930B | Bacterial infections | Remaining infections | Tuberculosis mesenterii |
| L00 | Bacterial infections | Remaining infections | Stafylokokbetinget exfoliativ dermatit |
| L009 | Bacterial infections | Remaining infections | Dermatitis exfoliativa staphylococcica |
| L009A | Bacterial infections | Remaining infections | Ritter's sygdom |
| L009C | Bacterial infections | Infections in wounds and skin | Staphylococcal scalded skin syndrome |
| L01 | Bacterial infections | Infections in wounds and skin | Børnesår |
| L010 | Bacterial infections | Infections in wounds and skin | Impetigo (alle organismer)(alle lokalisationer) |
| L010A | Bacterial infections | Remaining infections | Dactylitis, blistering distal (hæmol. strept. gr. A) |
| L010B | Bacterial infections | Infections in wounds and skin | Impetigo bullosa |
| L010C | Bacterial infections | Infections in wounds and skin | Impetigo palpebrae |
| L011 | Bacterial infections | Infections in wounds and skin | Dermatose m impetiginisering |
| L02 | Bacterial infections | Infections in wounds and skin | Bylder i huden |
| L020 | Bacterial infections | Infections in wounds and skin | Abscessus cutis furunculus et carbunculus faciei |
| L020A | Bacterial infections | Infections in wounds and skin | Furunculus faciei |
| L020B | Bacterial infections | Infections in wounds and skin | Carbunculus faciei |
| L020C | Bacterial infections | Infections in wounds and skin | Abscessus cutis faciei |
| L021 | Bacterial infections | Infections in wounds and skin | Abscessus cutis furunculus et carbunculus colli |
| L021A | Bacterial infections | Infections in wounds and skin | Furunculus colli |
| L021B | Bacterial infections | Infections in wounds and skin | Carbunculus colli |
| L021C | Bacterial infections | Infections in wounds and skin | Abscessus cutis colli |
| L022 | Bacterial infections | Infections in wounds and skin | Abscessus cutis furunculus et carbunculus trunci |
| L022A | Bacterial infections | Infections in wounds and skin | Furunculus parietis thoracis |
| L022B | Bacterial infections | Infections in wounds and skin | Furunculus perinei |
| L022C | Bacterial infections | Infections in wounds and skin | Furunculus parietis abdominis |
| L022D | Bacterial infections | Infections in wounds and skin | Furunculus inguinalis |
| L022E | Bacterial infections | Infections in wounds and skin | Furunculus umbilicalis |
| L022F | Bacterial infections | Infections in wounds and skin | Furunculus dorsi |
| L022G | Bacterial infections | Infections in wounds and skin | Carbunculus dorsi |
| L022H | Bacterial infections | Infections in wounds and skin | Carbunculus inguinalis |
| L022I | Bacterial infections | Infections in wounds and skin | Carbunculus parietis abdominis |
| L022J | Bacterial infections | Infections in wounds and skin | Carbunculus parietis thoracis |
| L022K | Bacterial infections | Infections in wounds and skin | Carbunculus perinei |
| L022L | Bacterial infections | Infections in wounds and skin | Carbunculus trunci |
| L022M | Bacterial infections | Infections in wounds and skin | Carbunculus umbilicalis |
| L022N | Bacterial infections | Infections in wounds and skin | Abscessus cutis dorsi |
| L022O | Bacterial infections | Infections in wounds and skin | Abscessus cutis umbilicalis |
| L022P | Bacterial infections | Infections in wounds and skin | Abscessus cutis trunci |
| L022Q | Bacterial infections | Infections in wounds and skin | Abscessus cutis perinei |
| L022R | Bacterial infections | Infections in wounds and skin | Abscessus cutis parietis abdominis |
| L022S | Bacterial infections | Infections in wounds and skin | Abscessus cutis parietis thoracis |
| L022T | Bacterial infections | Infections in wounds and skin | Abscessus cutis inguinalis |
| L023 | Bacterial infections | Infections in wounds and skin | Abscessus cutis furunculus et carbunculus reg glutaealis |
| L023A | Bacterial infections | Infections in wounds and skin | Furunculus glutaealis |
| L023B | Bacterial infections | Infections in wounds and skin | Carbunculus glutaealis |
| L023C | Bacterial infections | Infections in wounds and skin | Abscessus cutis glutaealis |
| L023D | Bacterial infections | Infections in wounds and skin | Abscessus cutis regionis glutaealis |
| L024 | Bacterial infections | Infections in wounds and skin | Abscessus cutis furunculus et carbunculus extremitatis |
| L024A | Bacterial infections | Infections in wounds and skin | Abscessus cutis hallucis |
| L024B | Bacterial infections | Infections in wounds and skin | Abscessus cutis femoris |
| L024C | Bacterial infections | Infections in wounds and skin | Abscessus cutis brachii |
| L024D | Bacterial infections | Infections in wounds and skin | Abscessus cutis axillae |
| L024E | Bacterial infections | Infections in wounds and skin | Abscessus cutis manus |
| L024F | Bacterial infections | Infections in wounds and skin | Abscessus cutis cruris |
| L024G | Bacterial infections | Infections in wounds and skin | Abscessus cutis digiti manus |
| L024H | Bacterial infections | Infections in wounds and skin | Abscessus cutis extremitatis superioris |
| L024I | Bacterial infections | Infections in wounds and skin | Abscessus cutis extremitatis inferioris |
| L024J | Bacterial infections | Infections in wounds and skin | Abscessus cutis digiti pedis |
| L024K | Bacterial infections | Infections in wounds and skin | Abscessus cutis antebrachii |
| L024L | Bacterial infections | Infections in wounds and skin | Abscessus cutis pollicis |
| L024M | Bacterial infections | Infections in wounds and skin | Abscessus cutis pedis |
| L028 | Bacterial infections | Infections in wounds and skin | Abscessus cutis furunculus et carbunculus capitis |
| L028A | Bacterial infections | Infections in wounds and skin | Furunculus capitis (undtagen ansigt) |
| L028B | Bacterial infections | Infections in wounds and skin | Carbunculus capitis (undtagen ansigt) |
| L028C | Bacterial infections | Infections in wounds and skin | Abscessus cutis capitis (undtagen ansigt) |
| L029 | Bacterial infections | Infections in wounds and skin | Abscessus cutis, furunculus et carbunculus u specifikation |
| L029A | Bacterial infections | Infections in wounds and skin | Furunculus uden specifikation |
| L029B | Bacterial infections | Infections in wounds and skin | Carbunculus uden specifikation |
| L029C | Bacterial infections | Infections in wounds and skin | Abscessus cutis uden specifikation |
| L03 | Bacterial infections | Infections in wounds and skin | Flegmone |
| L030 | Bacterial infections | Remaining infections | Phlegmone digiti manus et pedis |
| L030A | Bacterial infections | Remaining infections | Panaritium tendinosum |
| L030B | Bacterial infections | Remaining infections | Panaritium subunguale |
| L030C | Bacterial infections | Remaining infections | Paronychion |
| L030D | Bacterial infections | Remaining infections | Panaritium periunguale |
| L030E | Bacterial infections | Remaining infections | Onychia |
| L030F | Bacterial infections | Remaining infections | Panaritium articulare |
| L030G | Bacterial infections | Remaining infections | Panaritium digiti |
| L030H | Bacterial infections | Infections in wounds and skin | Phlegmone digiti pedis |
| L030I | Bacterial infections | Infections in wounds and skin | Phlegmone pollicis |
| L030J | Bacterial infections | Infections in wounds and skin | Phlegmone hallucis |
| L030K | Bacterial infections | Infections in wounds and skin | Phlegmone digiti manus |
| L031 | Bacterial infections | Infections in wounds and skin | Phlegmone extremitatis m anden lokalisation |
| L031A | Bacterial infections | Infections in wounds and skin | Phlegmone femoris |
| L031B | Bacterial infections | Infections in wounds and skin | Phlegmone manus, undtagen fingre |
| L031C | Bacterial infections | Infections in wounds and skin | Phlegmone extremitatis superioris, undtagen fingre |
| L031D | Bacterial infections | Infections in wounds and skin | Phlegmone pedis, undtagen tæer |
| L031E | Bacterial infections | Infections in wounds and skin | Phlegmone cruris |
| L031F | Bacterial infections | Infections in wounds and skin | Phlegmone axillae |
| L031G | Bacterial infections | Infections in wounds and skin | Phlegmone antebrachii |
| L031H | Bacterial infections | Infections in wounds and skin | Phlegmone brachii |
| L031I | Bacterial infections | Infections in wounds and skin | Phlegmone extremitatis inferioris, undtagen tæer |
| L032 | Bacterial infections | Infections in wounds and skin | Erysipelas faciei |
| L033 | Bacterial infections | Infections in wounds and skin | Phlegmone trunci |
| L033A | Bacterial infections | Infections in wounds and skin | Phlegmone dorsi |
| L033B | Bacterial infections | Infections in wounds and skin | Phlegmone inguinalis |
| L033C | Bacterial infections | Infections in wounds and skin | Phlegmone umbilicalis |
| L033D | Bacterial infections | Infections in wounds and skin | Phlegmone perinei |
| L033E | Bacterial infections | Infections in wounds and skin | Phlegmone parietis thoracis |
| L033F | Bacterial infections | Infections in wounds and skin | Phlegmone parietis abdominis |
| L038 | Bacterial infections | Infections in wounds and skin | Phlegmone m anden lokalisation |
| L038A | Bacterial infections | Infections in wounds and skin | Phlegmone capitis |
| L038B | Bacterial infections | Infections in wounds and skin | Erysipelas colli |
| L039 | Bacterial infections | Infections in wounds and skin | Phlegmone uden specifikation |
| L039A | Bacterial infections | Remaining infections | Cellulitis non spec. |
| L04 | Bacterial infections | Remaining infections | Akut betændelse i lymfeknude |
| L040 | Bacterial infections | Remaining infections | Lymphadenitis acuta faciei, capitis et colli |
| L040A | Bacterial infections | Remaining infections | Lymphadenitis acuta capitis |
| L040B | Bacterial infections | Remaining infections | Lymphadenitis acuta colli |
| L040C | Bacterial infections | Remaining infections | Lymphadenitis acuta faciei |
| L041 | Bacterial infections | Remaining infections | Lymphadenitis acuta trunci |
| L042 | Bacterial infections | Remaining infections | Lymphadenitis acuta extremitatis superioris |
| L042A | Bacterial infections | Remaining infections | Lymphadenitis acuta axillae |
| L043 | Bacterial infections | Remaining infections | Lymphadenitis acuta extremitatis inferioris |
| L048 | Bacterial infections | Remaining infections | Lymphadenitis acuta m anden lokalisation |
| L049 | Bacterial infections | Remaining infections | Lymphadenitis acuta uden specifikation |
| L050 | Bacterial infections | Remaining infections | Cystis pilonidalis m absces |
| L08 | Bacterial infections | Infections in wounds and skin | Infektioner i hud og underhud, andre lokale |
| L080 | Bacterial infections | Infections in wounds and skin | Pyodermia |
| L080a | Bacterial infections | Infections in wounds and skin | Pyodermia staphylococcica |
| L080B | Bacterial infections | Infections in wounds and skin | Ecthyma |
| L080C | Bacterial infections | Infections in wounds and skin | Ecthyma gangraenosum |
| L081 | Bacterial infections | Infections in wounds and skin | Erythrasma |
| L088 | Bacterial infections | Infections in wounds and skin | Infektioner i hud og underhud, andre specificerede lokale |
| L088A | Bacterial infections | Infections in wounds and skin | Ecthyma |
| L088B | Bacterial infections | Infections in wounds and skin | Ecthyma gangraenosum |
| L088C | Bacterial infections | Infections in wounds and skin | Pyoderma vegetans (Hallopeau) |
| L088D | Bacterial infections | Infections in wounds and skin | Perianal streptokokinfektion |
| L089 | Bacterial infections | Infections in wounds and skin | Infektioner i hud og underhud, lokale uden specifikation |
| L303 | Bacterial infections | Remaining infections | Dermatitis infectiosa |
| L738H | Bacterial infections | Infections in wounds and skin | Folliculitis (bakteriel) |
| M00 | Bacterial infections | Infections in joints and bones | Purulent ledbetændelse |
| M000 | Bacterial infections | Infections in joints and bones | Arthritis et polyarthritis staphylococcica |
| M000A | Bacterial infections | Infections in joints and bones | Arthritis staphylococcica |
| M000B | Bacterial infections | Infections in joints and bones | Polyarthritis staphylococcica |
| M001 | Bacterial infections | Infections in joints and bones | Arthritis et polyarthritis pneumococcica |
| M001A | Bacterial infections | Infections in joints and bones | Arthritis pneumococcica |
| M001B | Bacterial infections | Infections in joints and bones | Polyarthritis pneumococcica |
| M002 | Bacterial infections | Infections in joints and bones | Arthritis et polyarthritis streptococcica |
| M002A | Bacterial infections | Infections in joints and bones | Arthritis streptococcica |
| M002B | Bacterial infections | Infections in joints and bones | Polyarthritis streptococcica (excl pneumococcica) |
| M008 | Bacterial infections | Infections in joints and bones | Purulent artrit og polyartrit forårsaget af andre spec bakt |
| M009 | Bacterial infections | Infections in joints and bones | Purulent artrit uden specifikation |
| M01 | Bacterial infections | Infections in joints and bones | Infektion af led ved infektiøs/parasitær sygdom klass anste |
| M010 | Bacterial infections | Infections in joints and bones | Arthritis meningococcica |
| M011 | Bacterial infections | Infections in joints and bones | Arthritis tuberculosa |
| M012 | Bacterial infections | Infections in joints and bones | Artrit ved Borrelia infektion |
| M012A | Bacterial infections | Infections in joints and bones | Arthritis, Lyme sygdom |
| M013 | Bacterial infections | Infections in joints and bones | Artrit ved andre bakterielle sygdomme klass andetsteds |
| M013A | Bacterial infections | Infections in joints and bones | Arthritis typhoidea |
| M013B | Bacterial infections | Infections in joints and bones | Arthritis paratyphoidea |
| M013C | Bacterial infections | Infections in joints and bones | Arthritis, lokaliseret salmonella infektion |
| M013D | Bacterial infections | Infections in joints and bones | Arthritis, lepra |
| M014 | Bacterial infections | Remaining infections | Artrit ved Rubella infektion |
| M018 | Bacterial infections | Remaining infections | Artrit ved infektiøs og parasitær sygdom klass andetsteds |
| M021 | Bacterial infections | Remaining infections | Arthritis postdysenterica |
| M03 | Bacterial infections | Remaining infections | Postinfektiøs ledsygdom |
| M030 | Bacterial infections | Remaining infections | Arthritis postmeningococcica |
| M032 | Bacterial infections | Remaining infections | Postinfektiøse artritter, andre ved sygdom klass andetsteds |
| M032A | Bacterial infections | Remaining infections | Arthritis postinfectiosa hepatitica |
| M032B | Bacterial infections | Remaining infections | Arthritis postinfectiosa enteritica, Yersinia enterocolitic |
| M250 | Non-traumatic bleedings | Remaining bleedings | Haemarthrosis |
| M462 | Bacterial infections | Infections in joints and bones | Osteomyelitis vertebrae |
| M463 | Bacterial infections | Infections in joints and bones | Discitis intervertebralis (purulenta) |
| M463A | Bacterial infections | Infections in joints and bones | Discitis intervertebralis postoperativa |
| M464 | Bacterial infections | Infections in joints and bones | Diskit uden specifikation |
| M465 | Bacterial infections | Infections in joints and bones | Spondylitis infectiosa, anden form |
| M465A | Bacterial infections | Infections in joints and bones | Spondylitis infectiosa |
| M490 | Bacterial infections | Infections in joints and bones | Spondylitis tuberculosa |
| M491 | Bacterial infections | Infections in joints and bones | Spondylitis brucellosa |
| M492 | Bacterial infections | Infections in joints and bones | Spondylitis enterobacterialis |
| M493 | Bacterial infections | Infections in joints and bones | Spondylit ved infektiøs og parasitær sygdom klass andetsted |
| M493A | Bacterial infections | Infections in joints and bones | Spondylitis ved bakteriel sygdom klassificeret andetsteds |
| M600 | Bacterial infections | Remaining infections | Myositis infectiosa |
| M608A | Bacterial infections | Remaining infections | Absces i muskulatur |
| M608A1 | Bacterial infections | Remaining infections | Psoasabsces |
| M622 | Bacterial infections | Remaining infections | Infarctus musculi ischaemicus |
| M630 | Bacterial infections | Remaining infections | Myosit ved bakterielle sygdomme klassificeret andetsteds |
| M630A | Bacterial infections | Remaining infections | Myositis ved lepra |
| M632 | Bacterial infections | Remaining infections | Myosit ved andre infektiøse sygdomme klassific andetsteds |
| M650 | Bacterial infections | Remaining infections | Tenosynovitis purulenta |
| M651 | Bacterial infections | Remaining infections | Tenosynovitis infectiosa, anden |
| M680E | Bacterial infections | Remaining infections | Tenosynovitis tuberculosa |
| M680G | Bacterial infections | Remaining infections | Betændelse i ledkapselhinde ved bakteriel sygdom KA |
| M680H | Bacterial infections | Remaining infections | Betændelse i seneskedehinde ved bakteriel sygdom KA |
| M702 | Bacterial infections | Remaining infections | Bursitis olecrani |
| M710 | Bacterial infections | Remaining infections | Bursitis purulenta |
| M711 | Bacterial infections | Remaining infections | Bursitis infectiosa, anden |
| M86 | Bacterial infections | Infections in joints and bones | Knoglemarvsbetændelse |
| M860 | Bacterial infections | Infections in joints and bones | Osteomyelitis acuta haematogenes |
| M861 | Bacterial infections | Infections in joints and bones | Akut osteomyelit, anden |
| M862 | Bacterial infections | Infections in joints and bones | Osteomyelitis subacuta |
| M863 | Bacterial infections | Infections in joints and bones | Osteomyelitis chronica multifocalis |
| M864 | Bacterial infections | Infections in joints and bones | Osteomyelitis chronica med fistel |
| M865 | Bacterial infections | Infections in joints and bones | Kronisk osteomyelitis haematogenosa, anden |
| M865A | Bacterial infections | Infections in joints and bones | Osteomyelitis chronica haematogenosa uden specifikation |
| M866 | Bacterial infections | Infections in joints and bones | Kronisk osteomyelit, anden |
| M868 | Bacterial infections | Infections in joints and bones | Osteomyelit, anden |
| M868A | Bacterial infections | Infections in joints and bones | Abscessus Brodie |
| M869 | Bacterial infections | Infections in joints and bones | Osteomyelit uden specifikation |
| M869A | Bacterial infections | Infections in joints and bones | Abscessus periostalis uden osteomyelitis |
| M869B | Bacterial infections | Infections in joints and bones | Periostitis uden osteomyelitis |
| M869C | Bacterial infections | Infections in joints and bones | Osteitis uden osteomyelitis |
| M900 | Bacterial infections | Infections in joints and bones | Osteomyelitis tuberculosa |
| M901 | Bacterial infections | Infections in joints and bones | Periostit ved andre infektiøse sygdomme klass andetsteds |
| M902 | Bacterial infections | Remaining infections | Osteopati ved andre infektiøse sygdomme klass andetsteds |
| M902C | Bacterial infections | Infections in joints and bones | Osteomyelitis salmonellosa |
| M902D | Bacterial infections | Infections in joints and bones | Osteomyelitis echinococcica |
| N029 | Non-traumatic bleedings | Remaining bleedings | Haematuria recid et persist uden specifikation |
| N029A | Non-traumatic bleedings | Remaining bleedings | Haematuria persistens uden specifikation |
| N029B | Non-traumatic bleedings | Remaining bleedings | Haematuria recidivans uden specifikation |
| N080 | Bacterial infections | Remaining infections | Glomerulonefropati ved infek og parasit sygd klass ansted |
| N10 | Bacterial infections | Remaining infections | Akut nyrebækkenbetændelse |
| N109 | Bacterial infections | Remaining infections | Pyelonephritis acuta |
| N109A | Bacterial infections | Remaining infections | Akut infektiøs interstitiel nefritis |
| N109B | Bacterial infections | Remaining infections | Akut pyelitis |
| N109C | Bacterial infections | Remaining infections | Akut pyelonefritis |
| N11 | Bacterial infections | Remaining infections | Kronisk nyrebækkenbetændelse |
| N110 | Bacterial infections | Remaining infections | Pyelonephritis chronica non obstruct m vesicoureteral reflu |
| N110A | Bacterial infections | Remaining infections | Reflux vesicoureteralis med pyelonefrit |
| N111 | Bacterial infections | Remaining infections | Pyelonephritis chronica obstructiva |
| N111A | Bacterial infections | Remaining infections | Pyelonephritis chronica med stenosis ureteris juxtavesicali |
| N111B | Bacterial infections | Remaining infections | Pyelonephritis chronica med stenosis ureteris juxtapelvina |
| N112 | Bacterial infections | Remaining infections | Pyelonephritis recidivans |
| N118 | Bacterial infections | Remaining infections | Kronisk pyelonefrit, anden form |
| N118A | Bacterial infections | Remaining infections | Ikke-obstruktiv kronisk pyelonefritis UNS |
| N118B | Bacterial infections | Remaining infections | Recidiverende pyelonefritis |
| N118C | Bacterial infections | Remaining infections | Kronisk interstitiel nefritis |
| N118D | Bacterial infections | Remaining infections | Kronisk pyelitis |
| N119 | Bacterial infections | Remaining infections | Kronisk pyelonefrit uden specifikation |
| N12 | Bacterial infections | Remaining infections | Nyrebækkenbetændelse uden specifikation |
| N129 | Bacterial infections | Remaining infections | Pyelonephritis uden specifikation |
| N136 | Bacterial infections | Remaining infections | Pyonephrosis |
| N136A | Bacterial infections | Remaining infections | Hydronephrosis infectiosa |
| N136B | Bacterial infections | Remaining infections | Hydroureter infectiosa |
| N136C | Bacterial infections | Remaining infections | Stenosis ureteris infectiosa |
| N136D | Bacterial infections | Remaining infections | Strictura ureteris infectiosa |
| N136E | Bacterial infections | Remaining infections | Uropathia obstructiva infectiosa |
| N151 | Bacterial infections | Remaining infections | Abscessus renalis et perirenalis |
| N151A | Bacterial infections | Remaining infections | Abscessus perirenalis |
| N151B | Bacterial infections | Remaining infections | Abscessus renis |
| N160 | Bacterial infections | Remaining infections | Nephropathia ved infektiøs og parasitær sygd klass ansted |
| N200I | Bacterial infections | Remaining infections | Nefrolithiasis, infektiøs |
| N201I | Bacterial infections | Remaining infections | Ureterolithiasis, infektiøs |
| N202I | Bacterial infections | Remaining infections | Nefrolithiasis med ureterolithiasis, infektiøs |
| N280 | Thromboembolic conditions | Remaining thromboembolisms | Ischaemia et infarctus renis |
| N280A | Thromboembolic conditions | Remaining thromboembolisms | Embolia arteriae renalis |
| N280B | Thromboembolic conditions | Remaining thromboembolisms | Infarctus renis |
| N280C | Thromboembolic conditions | Remaining thromboembolisms | Ischaemia renis |
| N280D | Thromboembolic conditions | Remaining thromboembolisms | Thrombosis arteriae renalis |
| N30 | Bacterial infections | Remaining infections | Blærebetændelse |
| N300 | Bacterial infections | Remaining infections | Cystitis acuta |
| N301 | Bacterial infections | Remaining infections | Cystitis interstitialis chronica |
| N302 | Bacterial infections | Remaining infections | Kronisk cystit, anden |
| N303 | Bacterial infections | Remaining infections | Trigonitis |
| N303A | Bacterial infections | Remaining infections | Urethrotrigonitis |
| N308 | Bacterial infections | Remaining infections | Cystit, anden |
| N308A | Bacterial infections | Remaining infections | Abscessus vesicae urinariae |
| N308B | Bacterial infections | Remaining infections | Hæmorrhagisk cystit |
| N308C | Bacterial infections | Remaining infections | Cystitis recidivans |
| N308E | Bacterial infections | Remaining infections | Cystitis cystica |
| N308F | Bacterial infections | Remaining infections | Cystitis glandularis |
| N308G | Bacterial infections | Remaining infections | Cystitis planocellularis |
| N309 | Bacterial infections | Remaining infections | Cystit uden specifikation |
| N330 | Bacterial infections | Remaining infections | Cystitis tuberculosa |
| N34 | Bacterial infections | Remaining infections | Urinrørsbetændelse og urinrørssyndrom |
| N340 | Bacterial infections | Remaining infections | Abscessus urethrae |
| N340A | Bacterial infections | Remaining infections | Abscessus glandulae urethrae |
| N340B | Bacterial infections | Remaining infections | Abscessus periurethralis |
| N340C | Bacterial infections | Remaining infections | Carbunculus urethrae |
| N341 | Bacterial infections | Remaining infections | Urethritis non specificata |
| N342 | Bacterial infections | Remaining infections | Uretrit, anden form |
| N342A | Bacterial infections | Remaining infections | Meatitis urethrae |
| N342B | Bacterial infections | Remaining infections | Periurethritis |
| N342E | Bacterial infections | Remaining infections | Urethritis postmenopausalis |
| N342F | Bacterial infections | Remaining infections | Urethritis recidivans (non venerea) |
| N342G | Bacterial infections | Remaining infections | Urethritis uden specifikation |
| N351 | Bacterial infections | Remaining infections | Strictura urethrae postinfectiosa ikke klass andetsteds |
| N370 | Bacterial infections | Remaining infections | Uretrit ved sygd klassificeret andetsteds |
| N370A | Bacterial infections | Remaining infections | Urethritis, candidiasis |
| N390 | Bacterial infections | Remaining infections | Urinvejsinfektion u lokalisation |
| N390B | Bacterial infections | Remaining infections | Pyuria |
| N41 | Bacterial infections | Remaining infections | Betændelse i blærehalskirtel |
| N410 | Bacterial infections | Remaining infections | Prostatitis acuta |
| N411 | Bacterial infections | Remaining infections | Prostatitis chronica |
| N412 | Bacterial infections | Remaining infections | Abscessus prostatae |
| N412A | Bacterial infections | Remaining infections | Abscessus periprostaticus |
| N418 | Bacterial infections | Remaining infections | Prostatit, andre former |
| N419 | Bacterial infections | Remaining infections | Prostatit uden specifikation |
| N421 | Non-traumatic bleedings | Remaining bleedings | Haemorrhagia prostatae |
| N431 | Bacterial infections | Remaining infections | Hydrocele infectiosa |
| N45 | Bacterial infections | Remaining infections | Betændelse i sædkirtel og bitestikel |
| N450 | Bacterial infections | Remaining infections | Orchitis, epididymitis et epididymo-orchitis med absces |
| N450A | Bacterial infections | Remaining infections | Abscessus epididymidis |
| N450B | Bacterial infections | Remaining infections | Abscessus testis |
| N450C | Bacterial infections | Remaining infections | Epididymo-orchitis med absces |
| N450D | Bacterial infections | Remaining infections | Orchitis med absces |
| N459 | Bacterial infections | Remaining infections | Orchitis, epididymitis et epididymo-orchitis u absces |
| N459A | Bacterial infections | Remaining infections | Epididymitis acuta |
| N459B | Bacterial infections | Remaining infections | Epididymitis chronica |
| N459C | Bacterial infections | Remaining infections | Epididymo-orchitis |
| N459D | Bacterial infections | Remaining infections | Orchitis acuta |
| N459E | Bacterial infections | Remaining infections | Orchitis recidivans |
| N481 | Bacterial infections | Remaining infections | Balanoposthitis |
| N481A | Bacterial infections | Remaining infections | Balanitis |
| N481B | Bacterial infections | Remaining infections | Balanitis erosiva circinata |
| N481C | Bacterial infections | Remaining infections | Gangrænøs balanitis |
| N481D | Bacterial infections | Remaining infections | Plasmacellebalanitis |
| N482 | Bacterial infections | Remaining infections | Inflammatoriske sygd i penis, andre |
| N482A | Bacterial infections | Remaining infections | Abscessus corporis cavernosi penis |
| N482B | Bacterial infections | Remaining infections | Abscessus pelvis viri |
| N482C | Bacterial infections | Remaining infections | Abscessus penis |
| N482D | Bacterial infections | Remaining infections | Carbunculus corporis cavernosi penis |
| N482E | Bacterial infections | Remaining infections | Carbunculus penis |
| N482F | Bacterial infections | Remaining infections | Cavernitis penis |
| N482G | Bacterial infections | Remaining infections | Furunculus penis |
| N482H | Bacterial infections | Remaining infections | Phlegmone corporis cavernosi penis |
| N482I | Bacterial infections | Remaining infections | Phlegmone penis |
| N488G | Bacterial infections | Remaining infections | Thrombosis corporis cavernosi penis |
| N49 | Bacterial infections | Remaining infections | Betændelse i mandlige kønsorganer ikke klass ansted |
| N490 | Bacterial infections | Remaining infections | Vesiculitis seminalis |
| N491A | Bacterial infections | Remaining infections | Infectio ductus deferentis |
| N491B | Bacterial infections | Remaining infections | Infectio tunicae vaginalis |
| N492A | Bacterial infections | Remaining infections | Abscessus scroti |
| N492B | Bacterial infections | Remaining infections | Carbunculus scroti |
| N492C | Bacterial infections | Remaining infections | Furunculus scroti |
| N492D | Bacterial infections | Remaining infections | Infectio scroti |
| N498 | Bacterial infections | Remaining infections | Betændelse i andre specificerede dele af mandlige kønsorg |
| N498A | Bacterial infections | Remaining infections | Infectio multiplex genitalium viri |
| N498C | Bacterial infections | Remaining infections | Fournier gangræn |
| N501F | Thromboembolic conditions | Remaining thromboembolisms | Thrombosis genitalium viri |
| N508G | Bacterial infections | Remaining infections | Degeneratio testis postinfectiosa |
| N508X | Non-traumatic bleedings | Remaining bleedings | Hæmospermi |
| N510B | Bacterial infections | Remaining infections | Prostatitis trichomonalis |
| N510C | Bacterial infections | Remaining infections | Prostatitis tuberculosa |
| N511 | Bacterial infections | Remaining infections | Morbi testis et epididymidis ved sygd klass ansted |
| N511J | Bacterial infections | Remaining infections | Orchitis tuberculosa |
| N511K | Bacterial infections | Remaining infections | Epididymitis ved sygdom klassificeret andetsteds |
| N511L | Bacterial infections | Remaining infections | Orkitis ved sygdom klassificeret andetsteds |
| N512A | Bacterial infections | Remaining infections | Balanitis amoebica |
| N512B | Bacterial infections | Remaining infections | Balanitis, candidiasis |
| N61 | Bacterial infections | Remaining infections | Ikke pueperal betændelse i brystkirtel |
| N619A | Bacterial infections | Remaining infections | Abscessus areolae mammae non puerperalis |
| N619B | Bacterial infections | Remaining infections | Abscessus mammae non puerperalis |
| N619C | Bacterial infections | Remaining infections | Carbunculus mammae non puerperalis |
| N619D | Bacterial infections | Remaining infections | Inflammatio mammae non puerperalis |
| N619E | Bacterial infections | Remaining infections | Mastitis infectiosa non puerperalis |
| N619F | Bacterial infections | Remaining infections | Phlegmone mammae non puerperalis |
| N70 | Bacterial infections | Remaining infections | Betændelse i æggeleder og æggestok |
| N700 | Bacterial infections | Remaining infections | Salpingitis et oophoritis acuta |
| N700A | Bacterial infections | Remaining infections | Abscessus ovarii acutus |
| N700B | Bacterial infections | Remaining infections | Abscessus salpingis acutus |
| N700C | Bacterial infections | Remaining infections | Oophoritis acuta |
| N700D | Bacterial infections | Remaining infections | Pyosalpinx acuta |
| N700E | Bacterial infections | Remaining infections | Pyovarium acutum |
| N700F | Bacterial infections | Remaining infections | Salpingitis acuta |
| N700G | Bacterial infections | Remaining infections | Salpingo-oophoritis acuta |
| N701 | Bacterial infections | Remaining infections | Salpingitis et oophoritis chronica |
| N701A | Bacterial infections | Remaining infections | Abscessus ovarii chronicus |
| N701B | Bacterial infections | Remaining infections | Abscessus salpingis chronicus |
| N701D | Bacterial infections | Remaining infections | Oophoritis chronica |
| N701E | Bacterial infections | Remaining infections | Pyosalpinx chronica |
| N701F | Bacterial infections | Remaining infections | Pyovarium chronicum |
| N701G | Bacterial infections | Remaining infections | Salpingitis chronica |
| N701H | Bacterial infections | Remaining infections | Salpingo-oophoritis chronica |
| N709 | Bacterial infections | Remaining infections | Salpingitis et oophoritis uden specifikation |
| N709A | Bacterial infections | Remaining infections | Oophoritis uden specifikation |
| N709B | Bacterial infections | Remaining infections | Salpingitis uden specifikation |
| N709C | Bacterial infections | Remaining infections | Salpingo-oophoritis uden specifikation |
| N71 | Bacterial infections | Remaining infections | Betændelsestilstande i livmoder undtagen livmoderhals |
| N710 | Bacterial infections | Remaining infections | Endometritis acuta |
| N710A | Bacterial infections | Remaining infections | Abscessus uteri acutus |
| N710B | Bacterial infections | Remaining infections | Endometritis acuta corporis uteri |
| N710C | Bacterial infections | Remaining infections | Endometritis acuta purulenta |
| N710D | Bacterial infections | Remaining infections | Endomyometritis acuta |
| N710E | Bacterial infections | Remaining infections | Myometritis acuta |
| N710F | Bacterial infections | Remaining infections | Pyometra acuta |
| N711 | Bacterial infections | Remaining infections | Endometritis chronica |
| N711A | Bacterial infections | Remaining infections | Abscessus uteri chronicus |
| N711B | Bacterial infections | Remaining infections | Endometritis chronica purulenta |
| N711C | Bacterial infections | Remaining infections | Endometritis chronica corporis uteri |
| N711D | Bacterial infections | Remaining infections | Endometritis chronica senilis (atrophica) |
| N711E | Bacterial infections | Remaining infections | Endomyometritis chronica |
| N711F | Bacterial infections | Remaining infections | Pyometra chronica |
| N719 | Bacterial infections | Remaining infections | Endometritis uden specifikation |
| N72 | Bacterial infections | Remaining infections | Betændelsestilstande i livmoderhals |
| N729 | Bacterial infections | Remaining infections | Cervicitis uteri |
| N729A | Bacterial infections | Remaining infections | Cervicitis uteri acuta |
| N729B | Bacterial infections | Remaining infections | Cervicitis uteri chronica |
| N729C | Bacterial infections | Remaining infections | Endocervicitis |
| N729D | Bacterial infections | Remaining infections | Endocervicitis acuta |
| N729E | Bacterial infections | Remaining infections | Endocervicitis chronica |
| N729F | Bacterial infections | Remaining infections | Endometritis cervicis uteri |
| N73 | Bacterial infections | Remaining infections | Underlivsbetændelse hos kvinder, andre former |
| N730 | Bacterial infections | Remaining infections | Parametritis et phlegmone acuta pelvis feminae |
| N730A | Bacterial infections | Remaining infections | Abscessus acutus ligamenti lati |
| N730B | Bacterial infections | Remaining infections | Abscessus acutus parametrii |
| N730C | Bacterial infections | Remaining infections | Parametritis acuta |
| N730D | Bacterial infections | Remaining infections | Phlegmone acuta ligamenti lati |
| N730E | Bacterial infections | Remaining infections | Phlegmone acuta pelvis feminae |
| N731 | Bacterial infections | Remaining infections | Parametritis et phlegmone chronica pelvis feminae |
| N731A | Bacterial infections | Remaining infections | Abscessus chronicus ligamenti lati |
| N731B | Bacterial infections | Remaining infections | Abscessus chronicus parametrii |
| N731C | Bacterial infections | Remaining infections | Parametritis chronica |
| N731D | Bacterial infections | Remaining infections | Phlegmone chronica ligamenti lati |
| N731E | Bacterial infections | Remaining infections | Phlegmone chronica pelvis feminae |
| N732 | Bacterial infections | Remaining infections | Parametritis et phlegmone pelvis feminae uden specifikation |
| N732A | Bacterial infections | Remaining infections | Abscessus ligamenti lati uden specifikation |
| N732B | Bacterial infections | Remaining infections | Abscessus parametrii uden specifikation |
| N732C | Bacterial infections | Remaining infections | Parametritis uden specifikation |
| N732D | Bacterial infections | Remaining infections | Phlegmone ligamenti lati uden specifikation |
| N732E | Bacterial infections | Remaining infections | Phlegmone pelvis feminae uden specifikation |
| N733 | Bacterial infections | Remaining infections | Pelveoperitonitis acuta feminae |
| N733A | Bacterial infections | Remaining infections | Abscessus acutus fossae Douglasi |
| N734 | Bacterial infections | Remaining infections | Pelveoperitonitis chronica feminae |
| N734A | Bacterial infections | Remaining infections | Abscessus chronicus fossae Douglasi |
| N735 | Bacterial infections | Remaining infections | Pelveoperitonitis feminae uden specifikation |
| N738 | Bacterial infections | Remaining infections | Infektion i kvindelige bækken, anden specificeret |
| N738A | Bacterial infections | Remaining infections | Abscessus acutus pelvis feminae |
| N738B | Bacterial infections | Remaining infections | Abscessus chronicus pelvis feminae |
| N738C | Bacterial infections | Remaining infections | Abscessus pelvis feminae uden specifikation |
| N739 | Bacterial infections | Remaining infections | Pelveoperitonitis feminae uden specifikation |
| N74 | Bacterial infections | Remaining infections | Underlivsbetændelse hos kvinder ved sygd klass ansted |
| N740 | Bacterial infections | Remaining infections | Cervicitis uteri tuberculosa |
| N741 | Bacterial infections | Remaining infections | Pelveoperitonitis feminae tuberculosa |
| N741A | Bacterial infections | Remaining infections | Endometritis tuberculosa |
| N748 | Bacterial infections | Remaining infections | Pelveoperitonitis feminae ved andre sygd klass andetsteds |
| N751 | Bacterial infections | Remaining infections | Abscessus glandulae vestibularis majoris Bartholini |
| N758A | Bacterial infections | Remaining infections | Bartholinitis acuta |
| N758B | Bacterial infections | Remaining infections | Bartholinitis chronica |
| N758C | Bacterial infections | Remaining infections | Bartholinitis uden specifikation |
| N76 | Bacterial infections | Remaining infections | Betændelsessygd i skede og ydre kvindelige kønsdele, andre |
| N760 | Bacterial infections | Remaining infections | Vaginitis acuta |
| N760A | Bacterial infections | Remaining infections | Phlegmone genitalium externorum feminae |
| N760B | Bacterial infections | Remaining infections | Pyokolpos |
| N760C | Bacterial infections | Remaining infections | Vaginitis uden specifikation |
| N760D | Bacterial infections | Remaining infections | Vulvovaginitis acuta |
| N760E | Bacterial infections | Remaining infections | Vulvovaginitis uden specifikation |
| N761 | Bacterial infections | Remaining infections | Vaginitis subacuta et chronica |
| N761A | Bacterial infections | Remaining infections | Vaginitis chronica |
| N761B | Bacterial infections | Remaining infections | Vaginitis subacuta |
| N761C | Bacterial infections | Remaining infections | Vulvovaginitis chronica |
| N761D | Bacterial infections | Remaining infections | Vulvovaginitis subacuta |
| N761E | Bacterial infections | Remaining infections | Plasmacellevulvit |
| N762 | Bacterial infections | Remaining infections | Vulvitis acuta |
| N762A | Bacterial infections | Remaining infections | Vulvitis uden specifikation |
| N763 | Bacterial infections | Remaining infections | Vulvitis subacuta et chronica |
| N763A | Bacterial infections | Remaining infections | Vulvitis chronica |
| N763B | Bacterial infections | Remaining infections | Vulvitis subacuta |
| N764 | Bacterial infections | Remaining infections | Abscessus vulvae |
| N764A | Bacterial infections | Remaining infections | Abscessus labii majoris |
| N764B | Bacterial infections | Remaining infections | Abscessus labii minoris |
| N764C | Bacterial infections | Remaining infections | Furunculus labii majoris |
| N764D | Bacterial infections | Remaining infections | Furunculus labii minoris |
| N764E | Bacterial infections | Remaining infections | Furunculus vulvae |
| N768 | Bacterial infections | Remaining infections | Betændelsessygd i vagina og vulva, andre specificerede |
| N768A | Bacterial infections | Remaining infections | Abscessus vaginae |
| N768B | Bacterial infections | Remaining infections | Vaginosis bacterialis |
| N770 | Bacterial infections | Remaining infections | Ulcus vulvae ved infektiøse og parasitære sygd klass ansted |
| N770A | Bacterial infections | Remaining infections | Ulcus vulvae ved infektiøs sygdom klassificeret andetsteds |
| N770B | Bacterial infections | Remaining infections | Ulcus vulvae tuberculosum |
| N770C | Bacterial infections | Remaining infections | Ulcus vulvae ved parasitær sygdom klassificeret andetsteds |
| N770D | Bacterial infections | Remaining infections | Ulcus vulvae, herpesvirus |
| N771 | Bacterial infections | Remaining infections | Vaginitis og vulvitis ved infek og parasit sygd klass anste |
| N771A | Bacterial infections | Remaining infections | Vaginitis, candidiasis |
| N771B | Bacterial infections | Remaining infections | Vaginitis, herpesvirus |
| N771C | Bacterial infections | Remaining infections | Vaginitis, nematodes |
| N771D | Bacterial infections | Remaining infections | Vaginitis ved infektiøs sygdom klassificeret andetsteds |
| N771E | Bacterial infections | Remaining infections | Vaginitis ved parasitær sygdom klassificeret andetsteds |
| N771F | Bacterial infections | Remaining infections | Vulvitis, candidiasis |
| N771G | Bacterial infections | Remaining infections | Vulvitis, herpesvirus |
| N771H | Bacterial infections | Remaining infections | Vulvitis, nematodes |
| N771I | Bacterial infections | Remaining infections | Vulvitis ved infektiøs sygdom klassificeret andetsteds |
| N771J | Bacterial infections | Remaining infections | Vulvitis ved parasitær sygdom klassificeret andetsteds |
| N771K | Bacterial infections | Remaining infections | Vulvovaginitis, candidiasis |
| N771L | Bacterial infections | Remaining infections | Vulvovaginitis, herpesvirus |
| N771M | Bacterial infections | Remaining infections | Vulvovaginitis, nematodes |
| N771N | Bacterial infections | Remaining infections | Vulvovaginitis ved infektiøs sygdom klassificeret andetsted |
| N771O | Bacterial infections | Remaining infections | Vulvovaginitis ved parasitær sygdom klassificeret andetsted |
| N771P | Bacterial infections | Remaining infections | Vaginitis ved infektiøs eller parasitær sygdom KA |
| N771Q | Bacterial infections | Remaining infections | Vulvitis ved infektiøs eller parasitær sygdom KA |
| N771R | Bacterial infections | Remaining infections | Vulvovaginitis ved infektiøs eller parasitær sygdom KA |
| N858H | Bacterial infections | Remaining infections | Endometritis hyperplastica |
| N952 | Bacterial infections | Remaining infections | Vaginitis atrophica senilis |
| N952A | Bacterial infections | Remaining infections | Vaginitis atrophica postmenopausalis |
| N980 | Bacterial infections | Remaining infections | Infectiones inseminationis artificialis |
| R04 | Non-traumatic bleedings | Remaining bleedings | Blødning fra luftveje |
| R040 | Non-traumatic bleedings | Epistaxis | Epistaxis |
| R040A | Non-traumatic bleedings | Epistaxis | Epistaxis anterior |
| R040B | Non-traumatic bleedings | Epistaxis | Epistaxis posterior |
| R040C | Non-traumatic bleedings | Epistaxis | Epistaxis recidivans |
| R041 | Non-traumatic bleedings | Remaining bleedings | Haemorrhagia pharyngis |
| R042 | Non-traumatic bleedings | Remaining bleedings | Haemoptysis |
| R048 | Non-traumatic bleedings | Remaining bleedings | Blødning fra anden lokalisation i luftveje |
| R048A | Non-traumatic bleedings | Remaining bleedings | Haemorrhagia pulmonis |
| R049 | Non-traumatic bleedings | Remaining bleedings | Blødning fra luftveje uden specifikation |
| R091 | Bacterial infections | Infections in lower airways | Pleuritis |
| R093B | Bacterial infections | Infections in lower airways | Purulent opspyt |
| R31 | Non-traumatic bleedings | Remaining bleedings | Blod i urinen |
| R319 | Non-traumatic bleedings | Remaining bleedings | Haematuria uden specifikation |
| R319A | Non-traumatic bleedings | Remaining bleedings | Makroskopisk hæmaturi |
| R319B | Non-traumatic bleedings | Remaining bleedings | Mikroskopisk hæmaturi |
| R572 | Bacterial infections | Sepsis | Septisk shock |
| R58 | Non-traumatic bleedings | Remaining bleedings | Blødning ikke klassificeret andetsteds |
| R589 | Non-traumatic bleedings | Remaining bleedings | Haemorrhagia uden specifikation |
| R868C | Non-traumatic bleedings | Remaining bleedings | Haematospermia |
| T143C | Non-traumatic bleedings | Remaining bleedings | Haemarthrosis uden specifikation |
| T793 | Bacterial infections | Infections in wounds and skin | Posttraumatisk sårinfektion ikke klassificeret andetsteds |
| T802 | Bacterial infections | Infections in wounds and skin | Infektion efter infusion, transfusion og injektion |
| T802A | Bacterial infections | Infections in wounds and skin | Infectio post infusionem |
| T802B | Bacterial infections | Infections in wounds and skin | Infectio post injectionem |
| T802C | Bacterial infections | Infections in wounds and skin | Infectio post transfusionem |
| T802D | Bacterial infections | Sepsis | Sepsis post infusionem |
| T802D1 | Bacterial infections | Sepsis | CVK-relateret sepsis |
| T802E | Bacterial infections | Sepsis | Sepsis post injectionem |
| T802F | Bacterial infections | Sepsis | Sepsis post transfusionem |
| T802G | Bacterial infections | Remaining infections | CVK-relateret infektion |
| T814 | Bacterial infections | Remaining infections | Infektion efter indgreb ikke klassificeret andetsteds |
| T814A | Bacterial infections | Infections in wounds and skin | Abscessus in cicatrice |
| T814B | Bacterial infections | Infections in wounds and skin | Abscessus intraabdominalis postoperativus |
| T814C | Bacterial infections | Infections in wounds and skin | Abscessus subphrenicus postoperativus |
| T814D | Bacterial infections | Sepsis | Sepsis postoperativa |
| T814F | Bacterial infections | Infections in wounds and skin | Infectio vulneris postoperativa |
| T814G | Bacterial infections | Infections in wounds and skin | Postoperativ overfladisk sårinfektion |
| T814H | Bacterial infections | Infections in wounds and skin | Postoperativ dyb sårinfektion |
| T814I | Bacterial infections | Infections in wounds and skin | Postoperativ intraabdominal infektion |
| T814J | Bacterial infections | Infections in wounds and skin | Postoperativ retroperitoneal infektion |
| T814P | Bacterial infections | Infections in lower airways | Postoperativ pneumoni |
| T814U | Bacterial infections | Remaining infections | Postoperativ urinvejsinfektion |
| T814X | Bacterial infections | Remaining infections | Anden postoperativ infektion |
| T826 | Bacterial infections | Infections in wounds and skin | Infectio et reactio inflammatorica prostheseos valv cordis |
| T826A | Bacterial infections | Infections in wounds and skin | Infectio prostheseos valvae cordis |
| T827 | Bacterial infections | Infections in wounds and skin | Infectio et reactio inflam ved andre hjertekarprot/transpl |
| T827A | Bacterial infections | Infections in wounds and skin | Infektion ved hjertekarproteser |
| T827B | Bacterial infections | Infections in wounds and skin | Infektion ved hjertekartransplantater |
| T827I | Bacterial infections | Infections in wounds and skin | Infektion ved implanteret ICD-enhed |
| T827P | Bacterial infections | Infections in wounds and skin | Infektion ved implanteret pacemaker |
| T828A | Thromboembolic conditions | Remaining thromboembolisms | Trombose af a-v fistel |
| T835 | Bacterial infections | Infections in wounds and skin | Infect et react inflamm ved prot og transpl i urinveje |
| T835A | Bacterial infections | Infections in wounds and skin | Infektion ved implantater i urinveje |
| T835B | Bacterial infections | Infections in wounds and skin | Infektion ved proteser i urinveje |
| T835C | Bacterial infections | Infections in wounds and skin | Infektion ved transplantater i urinveje |
| T836 | Bacterial infections | Infections in wounds and skin | Infect et react inflamm ved prot og transpl i kønsorg |
| T836A | Bacterial infections | Infections in wounds and skin | Infektion ved implantater i kønsorganer |
| T836B | Bacterial infections | Infections in wounds and skin | Infektion ved proteser i kønsorganer |
| T836C | Bacterial infections | Infections in wounds and skin | Infektion ved transplantater i kønsorganer |
| T845 | Bacterial infections | Infections in wounds and skin | Infectio et reactio inflamm prostheseos internae articuli |
| T845A | Bacterial infections | Infections in wounds and skin | Infectio prostheseos internae articuli |
| T846 | Bacterial infections | Infections in wounds and skin | Infectio et reactio inflamm ved intern fiksation |
| T846A | Bacterial infections | Infections in wounds and skin | Infektion ved intern fiksation |
| T847 | Bacterial infections | Infections in wounds and skin | Infect et react inflamm ved andre interne prot og transpl |
| T857 | Bacterial infections | Infections in wounds and skin | Infect et react inflamm ved andre int proteser og transpl |
| T874 | Bacterial infections | Infections in wounds and skin | Infectio post amputationem |
| T880 | Bacterial infections | Infections in wounds and skin | Infectio post immunisationem |
| T880A | Bacterial infections | Sepsis | Sepsis post immunisationem |
| T89 | Bacterial infections | Remaining infections | Infektioner opstået i forbindelse med sygehusbehandling |
| T899 | Bacterial infections | Remaining infections | Nosokomiel infektion |
